# Pilot-scale inoculum-free valorization of raw chicken feathers: ammonium recovery, keratinase production and community dynamics

**DOI:** 10.1007/s11274-026-04976-0

**Published:** 2026-05-25

**Authors:** Fabio de-Santos-Casado, Carlos de la Fuente, Diego Martín-González, Sergio Bordel, Raúl Muñoz, Fernando Santos-Beneit

**Affiliations:** 1 Department of Chemical Engineering and Environmental Technology, School of Industrial Engineering, University of Valladolid, Dr. Mergelina, s/n, Valladolid, 47011 Spain; 2 Department of Microbiology and Genetics, University of Salamanca, Departmental Building, 2nd floor, Campus Miguel de Unamuno, Salamanca, 37007 Spain

**Keywords:** Chicken feathers, Ammonium, Keratinase, Inoculum-free, Waste valorization

## Abstract

Chicken feathers constitute a major keratin-rich agro-industrial residue whose sustainable valorization remains challenging due to the recalcitrant structure of keratin and the energy-intensive nature of conventional treatments. In this study, we investigated the feasibility of converting raw, mechanically untreated chicken feathers into soluble nitrogen compounds and extracellular keratinases through an inoculum-free microbial process operated at pilot scale. A 50 L horizontal bioreactor was run using only tap water and the indigenous feather-associated microbiota, without sterilization, nutrient supplementation or pretreatment, under alternating aerated and oxygen-limited regimes. Substantial feather degradation was achieved, accompanied by the accumulation of ammonium in the liquid phase up to 5 g L⁻¹ and keratinase activities exceeding 31,000 U mL⁻¹, particularly under oxygen-limited conditions. High-throughput 16 S rRNA gene sequencing revealed a compact but resilient core microbiota whose temporal succession correlated with process performance. Dominance shifts among *Comamonas*, *Wolinella*, *Tissierella* and *Pseudoxanthomonas* suggested functional complementarity between taxa involved in keratin hydrolysis, peptide fermentation and nitrogen mineralization. The progressive enrichment of anaerobic or facultative anaerobic members during oxygen limitation was associated with intensified ammonium release and sustained enzymatic activity. Overall, this work demonstrates that native feather-associated microbial consortia can self-organize into a functionally stable community capable of driving keratin depolymerization and nitrogen recovery in the absence of an external inoculum. These findings highlight the biotechnological potential of starter-free microbial systems for low-input valorization of keratinous wastes and contribute to a deeper understanding of community-level processes underpinning industrially relevant bioconversions.

**Graphical abstract:**

Conceptual scheme illustrating the proposed sustainable microbial biorefinery strategy for raw chicken feather valorization, highlighting inoculum-free operation, keratin hydrolysis, ammonium recovery and keratinase production in comparison with conventional feather disposal routes such as landfilling, incineration and high-temperature rendering

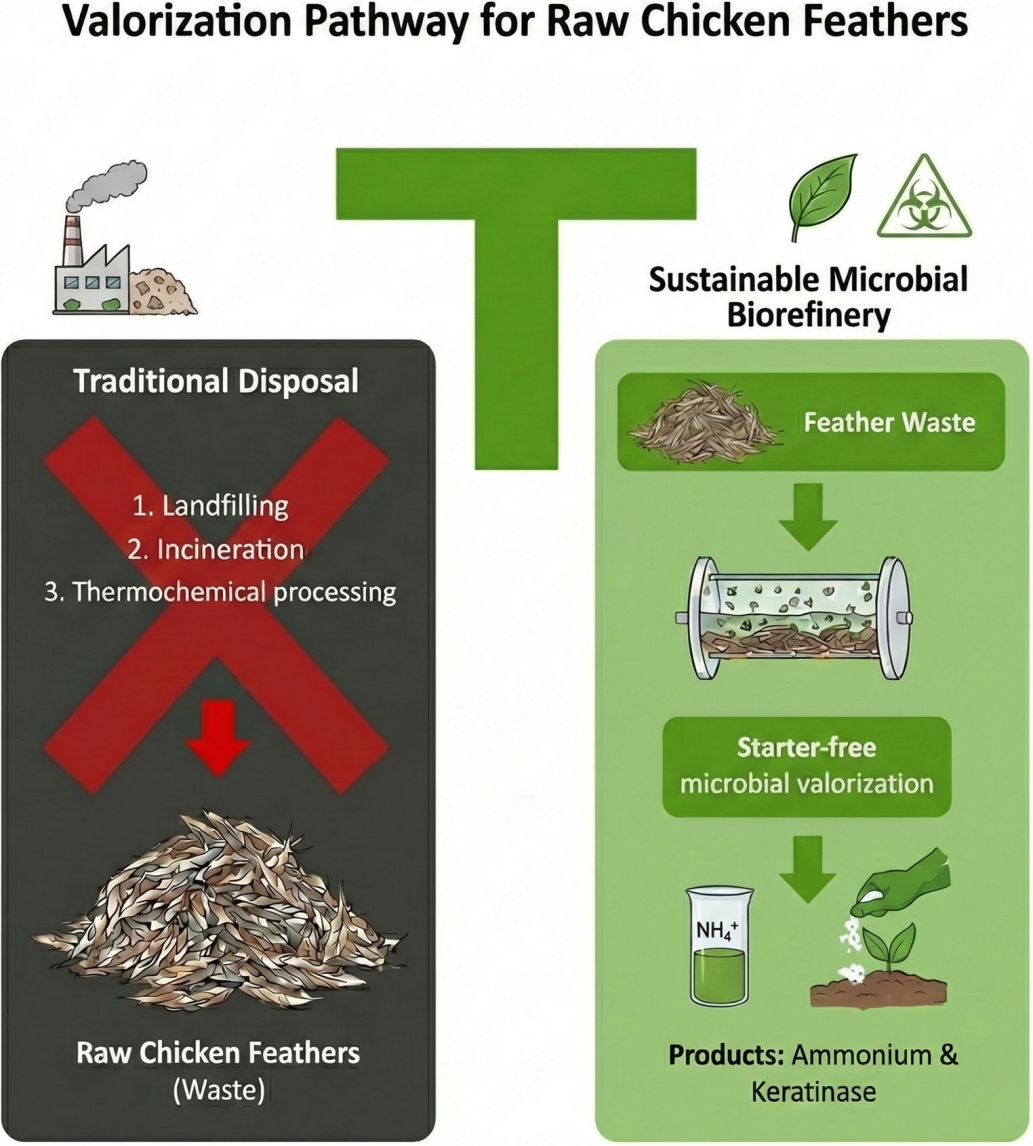

**Supplementary Information:**

The online version contains supplementary material available at 10.1007/s11274-026-04976-0.

## Highlights

Indigenous feather microbiota enabled pilot-scale keratin valorization without inoculation or pretreatment.

Oxygen limitation promoted ammonium accumulation and sustained keratinase production.

A resilient core microbiota governed process stability and functional succession.

A strong functional enrichment of feather-degrading microorganisms was observed throughout the valorization process, which supports the scalability and broad applicability of the proposed bioprocess.

**Supplementary Information:**

The online version contains supplementary material available at 10.1007/s11274-026-04976-0.

## Introduction

The poultry industry generates millions of tonnes of chicken feathers annually, making them one of the most abundant keratin-rich waste streams worldwide (Hemavarshini et al. 2025). Despite their high protein content—composed of up to 90% keratin and approximately 15% nitrogen—chicken feathers remain largely underutilized and are commonly disposed of through incineration, landfilling or low-value rendering processes (Bhari et al. 2021). These conventional practices are associated with significant environmental and economic drawbacks, including greenhouse gas emissions, odor generation, pathogen dissemination and high energy consumption (Yang et al. 2026; Wilkes et al. 2026). Although industrial rendering converts feathers into feather meal, it requires harsh thermal and pressure conditions that degrade essential amino acids and yield products of limited added value (de Souza et al. 2023).

Keratin, the main structural component of feathers, is a highly recalcitrant fibrous protein stabilized by extensive disulfide bonding, hydrogen bonding and hydrophobic interactions (Bansal and Singh 2016). While this structural complexity limits its natural biodegradation, it also presents an opportunity for biotechnological valorization under controlled conditions (Das et al. 2024). Microbial keratin degradation has therefore attracted considerable attention, as certain bacteria and fungi produce extracellular keratinases capable of hydrolyzing keratin into soluble peptides, amino acids and inorganic nitrogen (Vikash et al. 2025). These products are of high industrial relevance, particularly ammonium, which can be directly used as a nitrogen source in agriculture and biotechnological processes, and keratinolytic enzymes, which have applications in detergents, leather processing, cosmetics and waste treatment (de Menezes et al. 2021; Lyu et al. 2024). Indeed, numerous studies have reported the isolation and characterization of novel feather-degrading bacterial strains, as well as highlighted the biofertilizing potential of the resulting hydrolysates for sustainable agricultural production (Sahoo et al. 2012; Tamreihao et al. 2019; Możejko and Bohacz 2022; Martín-González et al. 2023a; Riaz et al. 2024; Otuyelu et al. 2025; Chu et al. 2026; Gupta and Sharma 2026).

To date, most studies on feather biodegradation have relied on pure microbial cultures—typically *Bacillus*, *Streptomyces* or related genera—grown under optimized laboratory conditions using defined or supplemented media (Ichida et al. 2001; Sahoo et al. 2012; Bordel et al. 2023; Martín-González et al. 2023b). In many cases, these systems also incorporate mechanical, thermal or chemical pretreatments to increase keratin accessibility, including alkaline hydrolysis, boiling, autoclaving or milling (Cheong et al. 2018). While such approaches can enhance degradation rates and enzyme production, they significantly increase process complexity, cost and energy demand, thereby limiting their scalability and industrial feasibility.

In contrast, raw chicken feathers naturally harbor diverse microbial communities adapted to keratin-rich environments, including bacteria with intrinsic keratinolytic capabilities (Sivakumar and Raveendran 2015; Bhari et al. 2021). The potential of these indigenous microbiota to drive feather degradation without external inoculation has been explored primarily in composting systems (Ichida et al. 2001; Tiquia et al. 2005; Franke-Whittle and Insam 2013; Choińska-Pulit et al. 2019). However, the role of inoculation in such processes remains controversial. Some studies have reported that the addition of keratinolytic strains (e.g., *Bacillus licheniformis* or *Streptomyces* spp.) can accelerate feather degradation (Ichida et al. 2001). In contrast, other studies have demonstrated that inoculation does not significantly affect composting performance, as native microbial communities are already sufficient to sustain efficient degradation (Tiquia et al. 2005). These findings suggest that the effectiveness of inoculation depends strongly on the initial microbial load and ecological dynamics of the system.

Despite this evidence, most experimental approaches continue to rely on controlled inoculation strategies, and truly inoculum-free (starter-free) systems remain poorly characterized. When reported, they are typically limited to low-controlled environments such as composting or anaerobic digestion, where feather degradation occurs as part of broader organic matter turnover rather than as a targeted valorization process (Schwede et al. 2017; El Salamony et al. 2024). Furthermore, these systems often focus on solid-state transformation rather than the generation of soluble, high-value products such as ammonium or enzymes. (Bhari et al. 2021).

From a circular economy perspective, there is therefore a clear need for minimalistic, robust and scalable processes that enable the direct conversion of keratin-rich waste into valuable biochemical products under realistic, non-sterile conditions. In this context, harnessing the native microbiota associated with raw feathers represents a promising alternative to conventional inoculum-based strategies, potentially eliminating the need for sterilization, nutrient supplementation and strain selection.

In this study, we investigate the microbial valorization of raw, mechanically untreated chicken feathers using only water and the native feather-associated microbiota in a 50 L pilot-scale horizontal bioreactor. The process was operated under both intermittently aerated and oxygen-limited conditions to assess their impact on keratin degradation, ammonium production, keratinase activity and microbial community dynamics. Particular emphasis was placed on the generation of soluble nitrogen compounds and enzymatic activity as indicators of process performance.

Although this study is based on a single pilot-scale experimental run, it was not conceived as a statistically replicated laboratory experiment aimed at defining a reproducible microbial consortium. Instead, the experimental framework was deliberately designed to emulate realistic industrial scenarios in which sterilization, mechanical pretreatments, defined media and external inocula are impractical or economically prohibitive. Under such conditions, the microbial community that develops is expected to depend strongly on the indigenous microbiota associated with the incoming waste stream and may therefore vary across locations and feedstocks. Consequently, this work does not aim to prescribe a universal microbial composition, but rather to demonstrate the functional feasibility, robustness and scalability of a starter-free keratin valorization strategy under real-world conditions.

## Materials and methods

### Chicken feather collection

Raw chicken feathers were collected at a private poultry slaughterhouse located in Valladolid, Spain, and immediately transported to the laboratory in a new, unused container (a visual summary of the collection procedure is provided in Supplementary Figure S1). Chicken feathers were not subjected to long-term storage at the slaughterhouse, as they are generated continuously during daily processing operations. Specifically, feathers are produced in real time as birds are mechanically defeathered along the processing line. Following mechanical defeathering, feathers are commonly transported via water-based conveying systems, after which excess water is partially removed through drainage or mechanical dewatering, and the material is subsequently directed to collection units for temporary storage prior to further processing or disposal. For the purposes of this study, feathers were collected directly on-site immediately after their generation, thereby preserving the native microbiota associated with the fresh material. Collection was performed manually using appropriate personal protective equipment (gloves, lab coat and face mask) to ensure both operator safety and sample integrity. The feathers were placed into a clean, unused container and transported without delay to the laboratory for immediate use. In the laboratory, feathers were used as received on a wet-weight basis, preserving the native microbiota naturally associated with the material. No washing, chemical treatment, mechanical grinding or sterilization was applied prior to reactor loading.

### Bioreactor design and operation

For this work, a novel pilot-scale horizontal bioreactor was specifically designed for the microbial valorization of raw chicken feathers and constructed by Mundo Metal (Valladolid, Spain) following detailed specifications provided by the authors. The horizontal configuration was selected to ensure effective and homogeneous mixing of a fibrous and high-solids-content substrate, minimizing dead zones and improving solid–liquid contact under conditions of limited flowability. The reactor consisted of a transparent horizontal cylindrical vessel with an approximate length of 1500 mm and an external diameter of 254 mm, allowing visual monitoring of feather degradation and biomass evolution throughout the experiment. The reactor chamber was equipped with two 4-cm-diameter ports installed along the length of the vessel, one at each end and positioned 180° apart. One port served as an inlet for the addition of water and feathers, while the opposite port functioned as an outlet for withdrawal of the generated hydrolysate. The reactor was mounted on adjustable support legs, enabling fine control of its inclination to facilitate mixing, drainage and operational stability.

Mixing was provided by a custom-designed horizontal agitator spanning the entire length of the reactor. The agitator shaft was fitted with four semi-elliptical blades distributed along its length to ensure homogeneous mixing of the feather–water matrix while minimizing mechanical stress. Special end seals were incorporated to guarantee proper operation at low rotational speeds. The agitator was driven by a gear motor equipped with a frequency inverter, allowing precise adjustment of stirring speed according to experimental requirements.

The reactor had a total nominal capacity of 50 L and was operated at a working volume corresponding to 10 L of tap water and 2 kg (wet weight) of raw, unprocessed chicken feathers (20% w/v). No mechanical pretreatment, washing or sterilization of the feathers was applied prior to loading. The feeding point at the top of the reactor allowed the input of feather wastes by gravity into the digester. The reactor was installed in a heated chamber operated in the mesophilic temperature range (35 °C ± 1 °C) and controlled by a temperature indicator. The reactor headspace was not purged prior to operation and initially reflected ambient atmospheric conditions. Agitation was set to 50 Hz throughout operation. The pH was not adjusted during the experiment.

During the first 50 days, the reactor was intermittently aerated by opening the inlet valve three times per week for 1 h to renew the headspace atmosphere. Water losses due to evaporation during aeration were experimentally determined to be negligible. Throughout the aerated phase, the reactor was operated in semi-continuous mode, with the volume of liquid withdrawn during weekly sampling replaced by an equivalent amount of tap water, while the initial feather load remained constant throughout the experiment. After day 50, aeration was completely stopped and the reactor was maintained under oxygen-limited conditions until day 100; no periodic sampling, as performed during the first 50 days, was carried out during this phase to avoid oxygen intrusion, and only a final sample was collected at day 100.

### Physicochemical analyses

Bulk culture samples were directly collected from the reactor outlet at defined time points (0, 1, 8, 15, 22, 29, 36, 43, 50 and 100 days). Samples consisted of heterogeneous mixtures of liquid and suspended solids (including feather fragments) directly withdrawn from the reactor. The liquid fraction was recovered by pipetting from the bulk sample, minimizing the inclusion of large solid particles, and subsequently centrifuged to remove residual suspended material. The resulting clarified supernatant was transferred to sterile tubes and used for subsequent physicochemical and enzymatic analyses. Therefore, all analytical results correspond exclusively to the soluble fraction of the reactor contents. This approach ensures that the measured parameters reflect the extent of keratin solubilization and nutrient release into the liquid phase, rather than the composition of the solid residual material.

Chromatographic quantification of free amino acids, ammonium (NH₄⁺), nitrate (NO₃⁻) and nitrite (NO₂⁻) in the supernatant was performed by chromatographic techniques following established analytical protocols for aqueous biological matrices. The analyses were conducted by the Instrumental Techniques Laboratory, Chemical Analysis Unit, of the University of Valladolid (Spain), which constitutes a centralized analytical service equipped with high-performance chromatographic systems that routinely supports research groups by providing high-resolution analyses of ionic species and organic compounds in liquid matrices using validated, accredited instrumental methods based on chromatographic separation and appropriate detection systems.

Dissolved inorganic carbon (DIC) and dissolved organic carbon (DOC) were quantified in filtered supernatant samples (0.45 μm) using a total organic carbon analyzer (Shimadzu TOC-VCSH, Japan) operated according to established in home protocols for water and wastewater carbon fraction analysis (García-Depraect et al. 2022). In these analyses, DIC is distinguished from DOC based on acidification and purge steps that release inorganic CO₂ prior to combustion and non-dispersive infrared detection, enabling accurate quantification of carbon species in liquid samples over a broad concentration range.

### Bacterial isolation and screening for keratinolytic activity

Throughout reactor operation, serial dilutions of freshly collected samples, at the following defined time points (1, 8, 15, 22, 29, 36, 43 and 100 days), were prepared in sterile saline solution and plated onto Luria–Bertani (LB) agar and Tryptic Soy Agar (TSA) to recover cultivable heterotrophic bacteria under non-selective growth conditions. Plates were incubated aerobically at 35 °C for 24–72 h.

Morphologically distinct colonies were repeatedly streaked to obtain pure cultures and subsequently preserved at − 20 °C in glycerol stocks for long-term storage. A total of 132 bacterial isolates were obtained over the course of the experiment (Supplementary Table S1). All isolates were subsequently screened for growth and keratinolytic activity using the enzymatic assays described in Sect.  2.5.

### Keratinase activity assay

Keratinase activity in reactor supernatant and pure bacterial culture extracts was measured using a well-established keratin substrate assay adapted from published protocols with proven suitability for environmental keratinolytic systems (e.g., Lin et al. 1992; Kembhavi et al. 1993). Briefly, filtered culture supernatant (0.5 mL) was mixed with 0.5 mL of 100 mM glycine–NaOH buffer (pH 10) containing 1% (w/v) casein as the substrate and incubated at 37 °C for 20 min. The reaction was terminated by adding 0.5 mL of 20% (w/v) trichloroacetic acid, and the mixture was allowed to stand at room temperature for 15 min before centrifugation at 13,000 rpm for 15 min to remove precipitate. The absorbance of the acid-soluble fraction was measured spectrophotometrically at 280 nm, and keratinase activity was quantified against a standard curve prepared with 0–700 mg L⁻¹ tyrosine equivalents.

One unit (U) of keratinase activity was defined as the amount of enzyme causing an increase of 0.01 absorbance units at 280 nm per minute under the assay conditions. This assay format provides a robust and reproducible quantitative assessment of extracellular keratinolytic activity in complex culture supernatants, consistent with standard enzymological practices in environmental biotechnology.

### DNA extraction and 16 S rRNA sequencing

Bacterial community composition throughout the experiment was investigated by 16 S rRNA gene amplicon sequencing. DNA extraction and sequencing were outsourced to the Genomics Unit of Fundación Parque Científico de Madrid (Spain).

In summary, total DNA was extracted using standard commercial protocols from the soluble fraction of a 20 mL bulk culture sample directly collected from the reactor and further quantified using fluorometrically methods to ensure sufficient yield and purity prior to downstream processing. The bacterial 16 S rRNA gene hypervariable V3–V4 region was amplified by PCR using universal primers widely adopted for microbial community profiling (Klindworth et al. 2013), followed by a second PCR step for Illumina-compatible indexing and sample barcoding. Amplicons were individually quantified, normalized and pooled equimolarly based on quantitative PCR measurements targeting the 16 S rRNA gene region, as described in previous studies (Kozich et al. 2013). The pooled library was purified to remove primers, nucleotides and short fragments and subsequently analyzed using an Agilent TapeStation system to confirm amplicon size distribution and library quality and to estimate final DNA concentration.

Sequencing was performed on an Illumina NextSeq 550 platform using paired-end chemistry (2 × 250–300 bp), allocating approximately 5% of a NextSeq vP1-600 run and targeting ca. 100,000 reads per sample and following standard high-throughput sequencing protocols for environmental microbiome analysis (Caporaso et al. 2012). Demultiplexing and primary quality filtering were carried out by the sequencing facility, which delivered paired FASTQ files (R1 and R2) for each sample. After processing, more than 250,000 high-quality reads per sample were obtained.

### Taxonomical analysis

Bioinformatic processing and taxonomic analyses of the sequencing data were performed by the Fundación Centro de Supercomputación de Castilla y León (FCSCL, León, Spain) using in-house pipelines implemented in R (v4.3.3), following previously described approaches for environmental microbiome datasets (Esteban-Blanco et al. 2020).

Paired-end reads were quality-filtered, merged and processed into amplicon sequence variants (ASVs) using established workflows for high-resolution microbial profiling (Callahan et al. 2016a). Taxonomic assignment was conducted against a curated reference database for prokaryotic 16 S rRNA genes (Quast et al. 2013). Downstream analyses were carried out using the phyloseq, vegan and microbiome packages (McMurdie and Holmes 2013; Callahan et al. 2016b).

Alpha diversity indices, including Shannon diversity, were calculated for each sample, while beta diversity patterns were explored using Bray–Curtis dissimilarity metrics and ordination approaches, as commonly applied in microbial ecology studies (Lozupone and Knight 2005). Relative abundance profiles were generated at phylum, genus and species levels, and dominant taxa were visualized across sampling times.

To identify persistent community members, the core microbiota was determined using prevalence and detection thresholds (taxa present in at least 80% of samples and with a minimum relative abundance of 0.1%), following standard definitions in microbiome research (Shade and Handelsman 2012). Core taxa were examined at both genus and species resolution, and their temporal abundance trajectories were analyzed to assess succession patterns and functional stability during reactor operation.

## Results and discussion

### Process performance and feather degradation

**Fig. 1:**
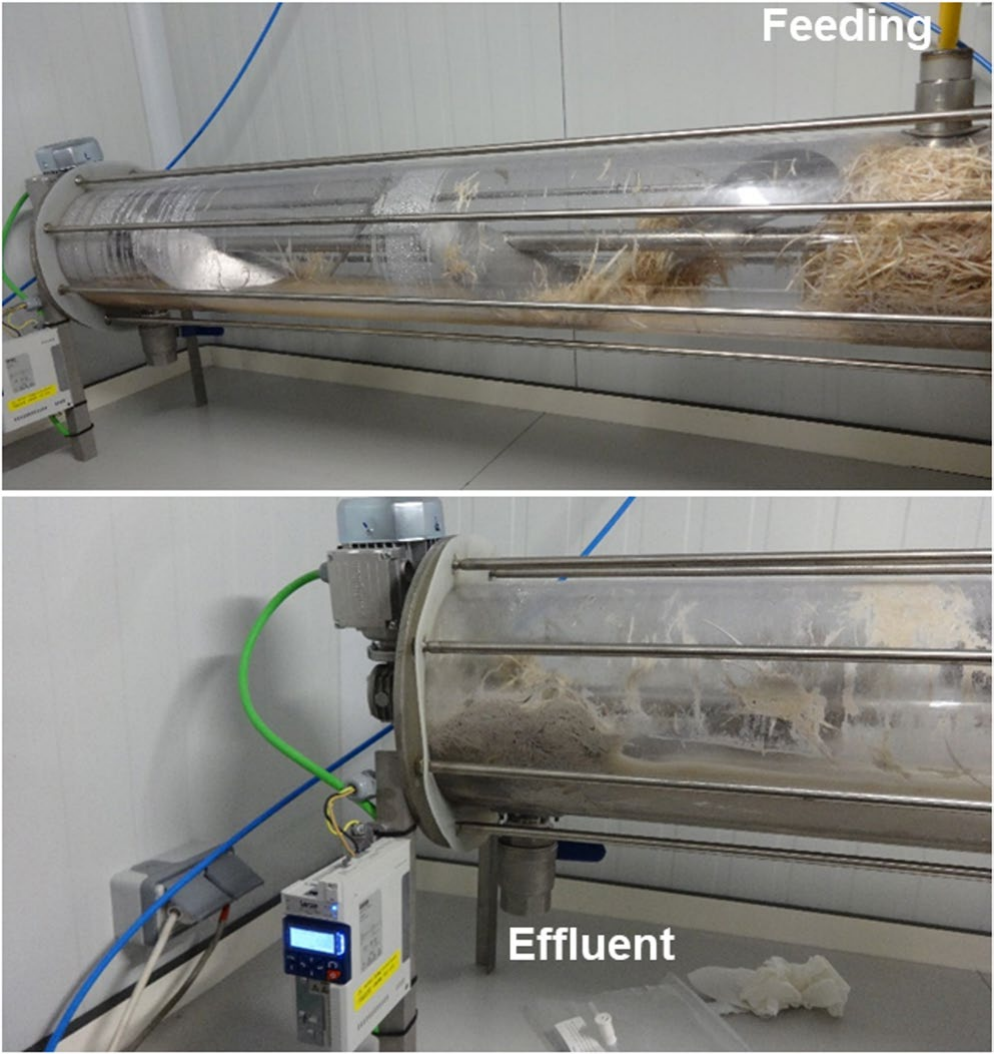
Pilot-scale horizontal bioreactor designed for the inoculum-free valorization of raw chicken feathers. The upper photograph shows the feeding port used for the addition of water and feathers, whereas the lower photograph depicts the effluent outlet used for hydrolysate withdrawal during semi-continuous operation

A horizontal pilot-scale bioreactor (see Fig. 1) was designed and constructed for the valorization of raw chicken feathers as described in Sect.  2.2. The system consisted of a transparent cylindrical vessel equipped with a full-length internal agitator bearing four semi-elliptical blades coupled to a variable-speed motor (see Video S1 in e-supplementary material).

The reactor was loaded with 10 L of tap water and 2 kg (wet weight) of untreated chicken feathers, corresponding to a solids content of 20% (w/v). Tap water was intentionally used in this study to better replicate realistic industrial operating conditions and to enhance the practical applicability of the process. In large-scale waste valorization systems, the use of distilled or deionized water is economically and logistically unfeasible due to the high volumes required and the associated energy and infrastructure costs. Moreover, tap water contains naturally occurring minerals and trace elements that may support microbial activity, thereby eliminating the need for additional nutrient supplementation. No external nutrients, buffering agents or microbial inocula were supplied at any stage of the experiment and pH was not externally controlled. The use of tap water is therefore consistent with the overall philosophy of the study, which aims to minimize external inputs while maximizing process feasibility at scale.

Since most of the keratinolytic microbes described to date are mesophiles; i.e. showing keratin degradation at 25–37 °C (Bhari et al. 2021), the temperature of the whole process was maintained at 35 °C and mixing was applied continuously at a constant rotation frequency of 50 Hz. During the first 50 days of operation, the reactor was subjected to intermittent aeration by opening the inlet valve three times per week for 1 h to renew the headspace atmosphere. This strategy was implemented to avoid the rapid establishment of strictly anaerobic conditions and to promote the development of a functionally diverse microbial community, including aerobic, microaerophilic and facultative anaerobic microorganisms involved in early-stage keratin hydrolysis. The intermittent nature of aeration allowed limited oxygen supply without shifting the system toward fully aerobic conditions, thereby maintaining a mixed redox environment representative of non-controlled industrial processes. This operational approach was intentionally selected to mimic realistic conditions in large-scale waste management processes, where oxygen transfer is typically limited and poorly controlled.

After day 50, aeration was completely stopped and the reactor was maintained under oxygen-limited conditions until day 100; with sampling restricted to the end of this period to avoid unintended oxygen introduction. This operational strategy enabled the assessment of prolonged oxygen limitation on feather degradation while minimizing external perturbations associated with sampling.

The pilot-scale horizontal bioreactor supported rapid and sustained degradation of raw, mechanically untreated chicken feathers without the addition of inocula, nutrients or pH control. Progressive disintegration of intact feathers was visually evident throughout the experiment, confirming that the indigenous microbiota alone was sufficient to colonize and depolymerize keratin-rich substrates at scale, underscoring the robustness of the process for direct valorization of poultry waste. The complete omission of mechanical processing, sterilization, chemical conditioning and nutrient supplementation represents a substantial advantage over previously reported feather treatment strategies and highlights the feasibility of operating simple and low-energy systems for keratin-rich waste valorization under mild mesophilic conditions.

### Nitrogen and carbon transformation during feather degradation

Because chicken feathers consist primarily of keratin, a structural protein rich in carbon and nitrogen, monitoring soluble carbon- and nitrogen-containing compounds in the liquid phase provides a direct proxy for keratin depolymerization and substrate valorization. All analyzed compounds are presented in Table 1 and plotted in Supplementary Figure S2.

**Table 1:** Temporal evolution of nitrogen- and carbon-related compounds during inoculum-free microbial valorization of raw chicken feathers in the pilot-scale bioreactor. Values correspond to concentrations measured in the reactor supernatant at the indicated sampling times. Nitrogen species include ammonium (NH₄⁺), nitrate (NO₃⁻) and nitrite (NO₂⁻). Carbon fractions include dissolved organic carbon (DOC) and dissolved inorganic carbon (DIC). Proline, the only free amino acid consistently detected throughout the cultivation period, is also included in the table

g.L^− 1^	T0	T1	T8	T15	T22	T29	T36	T43	T50	T100
Ammonium	0,039	0,187	0,687	1,327	1,854	2,030	1,804	2,080	2,085	4,966
Nitrate	0,000	0,001	0,001	0,001	0,001	0,001	0,001	0,001	0,002	0,003
Nitrite	0,000	0,002	0,003	0,004	0,005	0,004	0,003	0,003	0,002	0,003
DIC	0,081	0,119	0,219	0,324	0,422	0,402	0,372	0,415	0,440	0,803
DOC	0,498	0,699	1,018	1,165	1,231	1,254	1,249	1,247	1,246	1,666
Proline	0,000	0,010	0,000	0,010	0,010	0,030	0,030	0,030	0,020	0,060

**Fig. 2:**
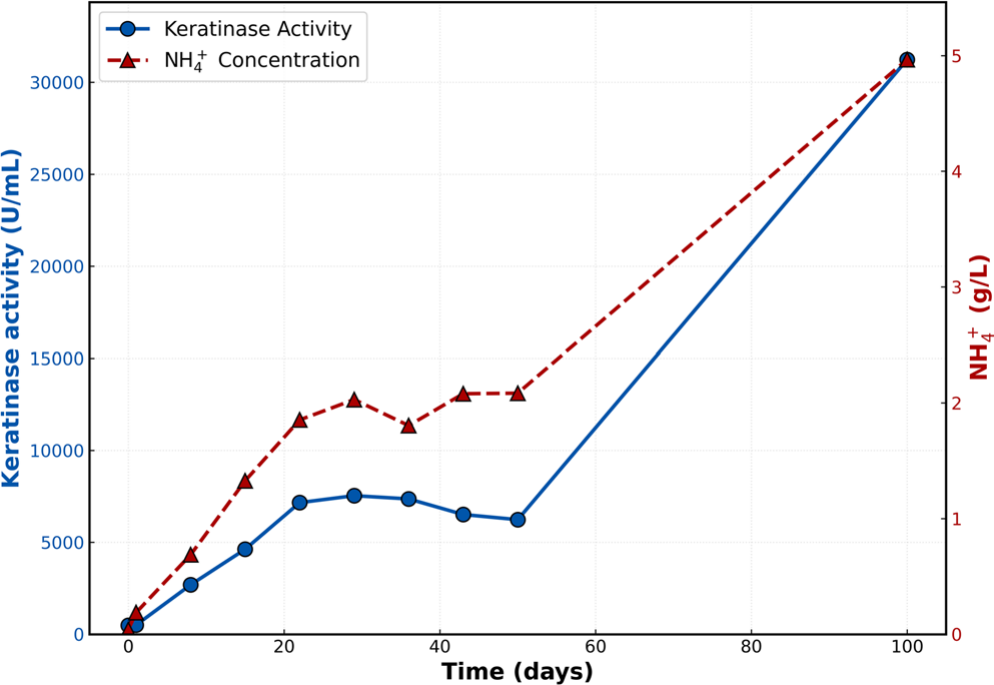
Temporal evolution of ammonium concentration (g L⁻¹) and keratinase activity (U mL⁻¹) in the reactor supernatant during the 100-day pilot-scale feather valorization process. The x-axis represents the incubation time, including the intermittently aerated phase (days 0–50) and the oxygen-limited phase (days 50–100)

Among all monitored parameters, ammonium accumulation emerged as the most sensitive and informative indicator of overall process performance. Ammonium concentration increased rapidly during the first month of operation, rising from 0.039 g L⁻¹ at inoculation to 2.03 g L⁻¹ by day 29. Between days 29 and 50, ammonium levels remained remarkably stable (1.80–2.09 g L⁻¹), suggesting the establishment of a dynamic steady state in which ammonium released from keratin degradation was largely balanced by microbial uptake under intermittently aerated conditions. Following the cessation of aeration, ammonium concentrations increased sharply, reaching 4.97 g L⁻¹ by day 100 (Fig. 2). This trend suggests that oxygen limitation favored fermentative pathways, enhancing amino acid deamination while reducing nitrogen assimilation into biomass. These findings are consistent with previous studies in anaerobic systems, where protein degradation leads to ammonia accumulation and inhibits microbial processes such as methanogenesis (Resch et al. 2011).

Oxidized nitrogen species remained negligible throughout the experiment: nitrate and nitrite concentrations never exceeded 0.005 g L⁻¹ (see Table 1), demonstrating that nitrification was minimal even during the aerated phase and that nitrogen was retained almost exclusively in reduced form.

Dissolved organic carbon increased from 0.50 g L⁻¹ initially to 1.25 g L⁻¹ by day 29 and subsequently stabilized until day 50, mirroring the ammonium plateau observed during aeration. Under oxygen-limited conditions, organic carbon increased further to 1.67 g L⁻¹ by day 100, reflecting continued solubilization of keratin-derived compounds. In parallel, dissolved inorganic carbon rose from 0.08 g L⁻¹ at the start to 0.42 g L⁻¹ by day 22, remained relatively constant until day 50, and then increased markedly to 0.80 g L⁻¹ at day 100, consistent with intensified fermentative metabolism and CO₂ accumulation in the closed system.

Free amino acids accumulated only transiently in the supernatant and at low concentrations. Proline was the sole amino acid detected throughout the experiment (< 0.06 g L⁻¹), while cysteine was observed only at day 22 (0.01 g L⁻¹) and valine appeared briefly at days 36 and 50 (0.04 and 0.03 g L⁻¹, respectively). The limited accumulation of soluble amino acids, despite high keratinase activity and substantial ammonium release, suggests rapid microbial uptake and subsequent metabolism. This pattern highlights a tight coupling between extracellular keratin depolymerization and intracellular nitrogen mineralization.

Phosphorus and sulfur species were not comprehensively quantified in this study. Inorganic phosphate (PO₄³⁻) was assessed semi-quantitatively using colorimetric methods (malachite green assay), consistently confirming its presence in the liquid phase throughout the process, although the complexity of the reactor matrix prevented reliable quantitative interpretation. From a biochemical perspective, phosphorus in feather-based systems might be present at much lower levels than carbon and nitrogen and might be rapidly assimilated into microbial biomass or incorporated into intracellular metabolic processes, thereby limiting its accumulation in the dissolved phase. This behavior is consistent with the well-established role of phosphorus as a frequently limiting nutrient in microbial ecosystems, where microorganisms tightly regulate its uptake, storage and recycling to sustain growth and metabolic activity (Ordóñez-Robles et al. 2017). Consequently, microbial biomass tends to maintain relatively constrained C: N:P stoichiometric ratios, promoting efficient phosphorus scavenging and minimizing its accumulation in the aqueous phase compared to more mobile nitrogen species such as ammonium (Griffiths et al. 2012).

Similarly, sulfur dynamics were not directly monitored; however, under the predominantly oxygen-limited conditions applied, sulfur released from keratin is expected to be mainly converted into reduced forms such as hydrogen sulfide via cysteine desulfhydration pathways, with potential volatilization or incorporation into biomass rather than accumulation as sulfate (Tran et al. 2023). Notably, the consistent perception of a characteristic malodor during reactor aeration events indicates the likely formation and release of hydrogen sulfide. Overall, these considerations suggest that both phosphorus and sulfur were actively cycled within the microbial community but did not accumulate as major soluble products under the operational conditions tested. Future studies incorporating a complete sulfur mass balance, including sulfate, sulfide and volatile sulfur compounds, would provide a more comprehensive understanding of sulfur transformations during keratin valorization.

### Keratinase activity

Keratinase activity was detected in the reactor supernatant from the earliest stages of operation and increased markedly over time, confirming sustained enzymatic attack on native chicken feathers by the indigenous microbial community. Initial activity at time zero was low (488 U mL⁻¹), reflecting basal extracellular enzyme levels associated with the native microbiota present on raw feathers. A modest increase was observed after 24 h (502 U mL⁻¹), followed by a rapid intensification during the first two weeks of operation. Between days 8 and 22, keratinase activity increased from 2,689 to 7,160 U mL⁻¹, coinciding with the period of accelerated ammonium release and organic carbon solubilization.

Maximum keratinase activity under intermittently aerated conditions was reached between days 29 and 36 (7,539–7,361 U mL⁻¹), after which a gradual decline was observed toward day 50 (6,234 U mL⁻¹). This trajectory parallels the stabilization of ammonium concentrations during the aerated phase, suggesting the establishment of a metabolic equilibrium between keratin hydrolysis, microbial growth and nitrogen assimilation (Fig. 2).

Following the transition to oxygen-limited operation, keratinase activity increased dramatically, reaching 31,238 U mL⁻¹ by day 100—approximately five-fold higher than the maximum values measured during aeration. This pronounced enhancement indicates that oxygen limitation strongly favored either the expression, secretion or persistence of extracellular keratinases in the reactor, and directly supports the intensified ammonium release observed during the final stage of the process.

### Microbiata analysis

Alpha-diversity dynamics throughout the experiment were assessed using the Shannon index (Supplementary Figure S3). Microbial diversity varied markedly over time, with the highest values observed at T22 (Shannon index = 4.7), coinciding with the most active phase of feather degradation, and the lowest at T50 (2.8). Notably, following the transition from intermittent aeration to oxygen-limited operation, alpha diversity increased again to 3.7 at T100, indicating substantial community restructuring and adaptation to the new redox regime.

**Fig. 3:**
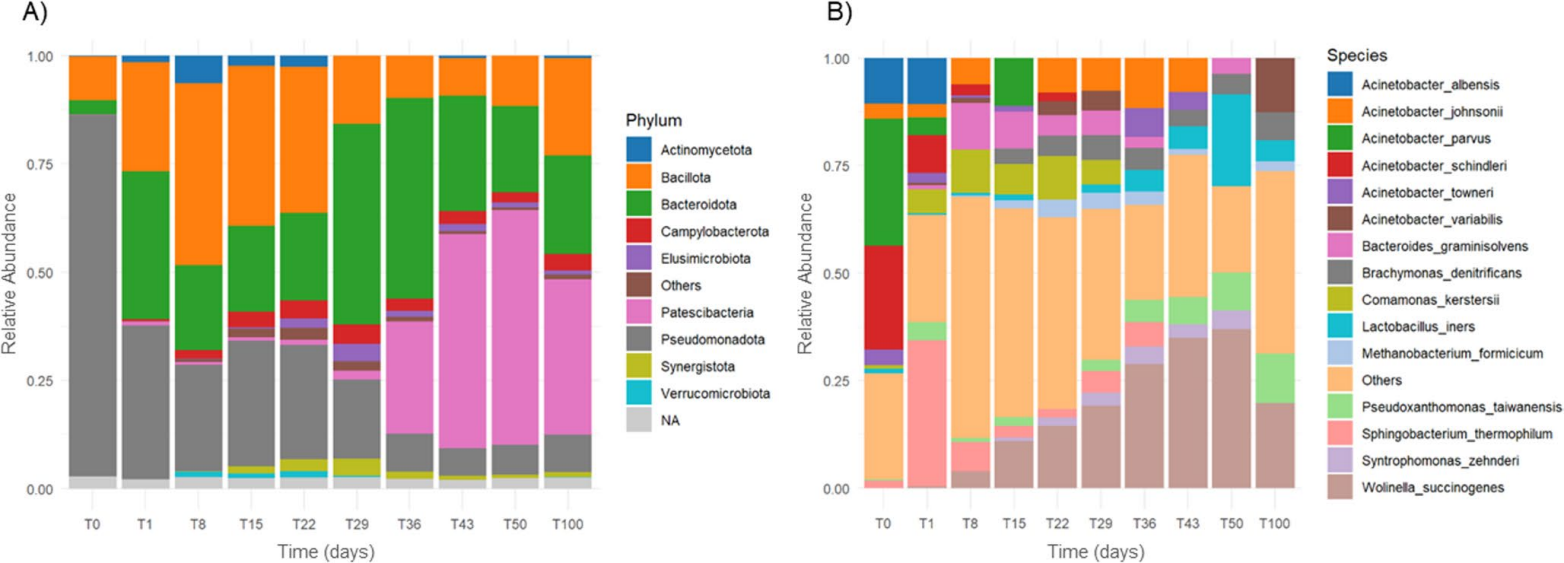
Barplot representation of the overall bacterial community dynamics during the pilot-scale inoculum-free feather valorization process. (**A**) Relative abundance at the phylum level. (**B**) Relative abundance at the species level. These profiles represent the total detected microbiota across all sampling points, capturing the full diversity and temporal shifts of the microbial community. The most abundant taxa at each taxonomic rank are displayed to the right of the corresponding panels

At the start of the experiment (T0), the microbial community was dominated by the phylum Pseudomonadota, representing approximately 85% of the total relative abundance (Fig. 3A). This highly diverse group of Gram-negative bacteria includes both free-living and opportunistic taxa commonly associated with environmental and slaughterhouse microbiota, consistent with the indigenous microbial load present on raw feathers (Nasipuri et al. 2020). Bacillota (10%) and Bacteroidota (3%) were also detected as relevant phyla at T0.

As the process progressed, the relative abundance of Pseudomonadota gradually declined, while Bacillota and Bacteroidota became predominant across most sampling points, reflecting a functional transition toward microbial groups typically associated with protein depolymerization and fermentative or facultatively anaerobic metabolisms (Nakasaki et al. 2019). Members of Bacillota are widely recognized for their capacity to degrade complex organic substrates and secrete extracellular proteases and keratinases (Bordel et al. 2023; Martín-González et al. 2023a), whereas Bacteroidota frequently participate in the breakdown of high-molecular-weight organic matter and peptides during prolonged fermentation processes (McKee et al. 2021).

From T43 onward, the phylum Patescibacteria—also referred to as the Candidate Phyla Radiation (CPR)—became dominant. This highly diverse and largely uncultivated bacterial radiation is characterized by reduced genomes and limited metabolic repertoires, and cultivated representatives are frequently obligate epibionts that rely on close metabolic coupling with host bacteria (Ji et al. 2022). In the present system, CPR members increased markedly between T29 and T36, coinciding with a pronounced decline in Pseudomonadota, and subsequently dominated the community after T43, suggesting strong dependence on metabolites released by primary degraders. Rather than reflecting direct competition, this inverse relationship likely represents a process of ecological succession. During the early stages of operation, metabolically versatile and fast-growing taxa affiliated with Pseudomonadota dominated the system, actively contributing to keratin hydrolysis and the release of soluble organic compounds and nitrogenous products. As the process progressed and readily degradable substrates became depleted, the relative abundance of these primary degraders declined, leading to the establishment of more specialized ecological niches. Under these conditions, CPR members—well adapted to resource-limited environments and reliant on syntrophic interactions—proliferated by scavenging low-molecular-weight metabolites such as organic acids, amino acids and other fermentation intermediates.

Additional phyla detected throughout the experiment included Actinomycetota, Campylobacterota, Synergistota, Verrucomicrobiota and Elusimicrobiota. Actinomycetota were mainly detected at T1, T8, T15 and T22 and comprise Gram-positive bacteria renowned for their great capacity not only to synthetize a wide range of secondary metabolites (Ordóñez-Robles et al. 2018) but also to degrade complex polymers such as cellulose, lignin and keratin, supporting their likely contribution during the early stages of feather depolymerization (Martín-González et al. 2023b). Campylobacterota encompass microaerophilic taxa frequently associated with low-oxygen niches and organic acid turnover (van der and Wösten 2019), while Synergistota are anaerobic bacteria typically involved in amino acid fermentation (McSweeney et al. 2025). Verrucomicrobiota consist largely of free-living organisms found in aquatic and terrestrial habitats (Naziębło et al. 2025), whereas Elusimicrobiota include fermentative taxa associated with animal hosts as well as free-living representatives in soils and sediments (Mies et al. 2025).

At the species level, *Acinetobacter albensis*, *Acinetobacter parvus* and *Acinetobacter schindleri* dominated the community at T0 (Fig. 3B), reflecting the indigenous microbiota associated with raw, unprocessed feathers. As the process progressed, *Wolinella succinogenes* emerged as the most abundant species overall, although its relative abundance declined markedly by day 100. This acid-utilizing, succinate-producing bacterium is well adapted to low-oxygen environments and complex organic substrates and likely contributed to metabolic stabilization during feather degradation by consuming organic acids and preventing excessive acidification (Kröger et al. 2002). In line with this interpretation, pH remained stable between 7.5 and 7.8 throughout the entire experiment, indicating effective buffering by the microbial consortium and maintenance of conditions favorable for sustained enzymatic activity.

Other species detected intermittently included *Bacteroides graminisolvens*, *Brachymonas denitrificans*,* Comamonas kerstersii*,* Lactobacillus iners*,* Methanobacterium formicicum*,* Pseudoxanthomonas taiwanensis*,* Sphingobacterium thermophilum* and *Syntrophomonas zehnderi*. Among these, only *W. succinogenes*, *P. taiwanensis* and *L. iners* were consistently detected across all sampling points and were therefore defined as core species (see Sect.  3.5).

### Core microbiota and functional stability

**Fig. 4:**
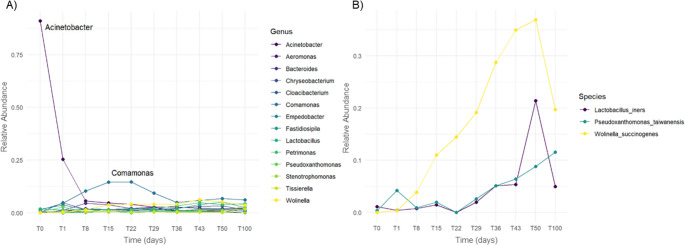
Temporal dynamics of the core microbiota during the valorization process. The core microbiota was defined as taxa consistently detected across all sampling points, representing a stable and functionally relevant microbial consortium. (**A**) Genus-level relative abundance profiles of the 14 core genera, showing pronounced succession patterns over time. *Acinetobacter* dominated the initial community associated with raw feathers (T0) but rapidly declined during the first week, concomitant with the emergence of *Comamonas* as the prevailing genus from T8 to T29. (**B**) Species-level relative abundance patterns of the three core species, highlighting differential responses to operational conditions and their contribution to long-term process stability. *Wolinella succinogenes* and *Lactobacillus iners* decreased sharply following cessation of aeration, whereas *Pseudoxanthomonas taiwanensis* increased progressively under both aerated and oxygen-limited regimes

Analysis of the core microbiota identified 14 genera and 3 species that persisted throughout the entire experiment (Supplementary Figures S4 and S5, respectively), highlighting the presence of a relatively small but stable and resilient microbial consortium. Genus-level metataxonomic profiles revealed pronounced temporal succession patterns (Fig. 4A). At T0, the community was dominated by *Acinetobacter* spp., accounting for more than 75% of the relative abundance of the core microbiota and reflecting the microbial load associated with raw feathers. During the first week of cultivation, *Acinetobacter* abundance declined sharply, coinciding with a rapid increase in *Comamonas* spp., which became the dominant genus at T8, T15, T22 and T29.

*Comamonas* spp. are frequently encountered in nutrient-rich environments and are known to participate in protein and nitrogen compound metabolism. Several members of this genus display broad proteolytic capacities and have been implicated in the degradation of complex and recalcitrant substrates, including chicken feathers (Wang et al. 2019). In the present system, the temporal increase in *Comamonas* abundance closely paralleled the progressive rise in ammonium concentrations and keratinase activity (Figs. 2 and 4A), both of which increased steadily until approximately day 22 before stabilizing. This strong temporal correspondence supports a substantial contribution of *Comamonas* spp. to early-stage keratin depolymerization and nitrogen release.

A comparative analysis of microbial abundance before and after the transition to oxygen-limited conditions revealed a marked functional shift within the core microbiota following the transition to oxygen-limited conditions, with *Tissierella* emerging as the main genus significantly enriched during the final phase of operation (Supplementary Figure S4). Members of this genus are strict or facultative anaerobes capable of amino acid fermentation and nitrogen mineralization, consistent with their involvement in ammonium production from keratin-derived peptides (Bonatti et al. 2025). In support of this interpretation, *Tissierella* has recently been reported to become selectively enriched during alkaline sludge fermentation under mixing regimes that enhanced protein hydrolysis and volatile fatty acid production, where it was identified as a key functional taxon involved in organic nitrogen conversion (Ma et al. 2023).

In contrast, core species such as *W. succinogenes* and *L. iners* declined sharply once aeration ceased (Fig. 4B), suggesting a dependence on aerobic and microaerobic conditions. Conversely, the core species *P. taiwanensis* increased steadily throughout both aerated and oxygen-limited phases (Fig. 4B), in line with its reported tolerance to low-oxygen environments (Neves et al. 2024). This pattern indicates that *P. taiwanensis* likely contributed to sustained keratin utilization not only during the intermittently aerated phase but also during the prolonged oxygen-limited stage, thereby supporting the long-term functional stability of the process.

Overall, the core microbiota seems to constitute a compact yet metabolically versatile consortium whose coordinated succession and functional complementarity—particularly among *Comamonas*, *Tissierella*, *Wolinella* and *Pseudoxanthomonas*—may underpin efficient keratin degradation, nitrogen mineralization and operational stability. Therefore, these findings seem to demonstrate that feather valorization is not driven by isolated keratinolytic specialists alone, but by a self-organized native community capable of adapting to shifting redox conditions and sustaining performance over extended timeframes, which reinforces the suitability of this low-input strategy for non-sterile, industrial-scale implementation.

### Isolation of keratinolytic bacteria

To assess whether feather degradation in the bioreactor was driven by a highly diverse microbial community or by a limited number of specialized strains, a total of 132 bacterial isolates were recovered over the fourteen-week experimental period and screened for keratinolytic activity (Supplementary Table S1). With only two exceptions, all isolates exhibited measurable and significant keratinase activity, indicating widespread keratinolytic potential within the cultivated microbiota. Notably, 35% of the isolates displayed keratinase activities exceeding 5000 U mL⁻¹ in the in vitro assays, reflecting a strong functional enrichment of highly active feather-degrading microorganisms throughout the valorization process (Supplementary Table S1 and Supplementary Figure S6). This threshold (i.e. 5000 U mL⁻¹) was selected as a conservative benchmark based on previous studies from our group, as well as on other reports using identical assay conditions, in which activities in the range of 3000–5000 U mL⁻¹ were already considered indicative of high keratinolytic potential (Lin et al. 1992; Hmidet et al. 2010; Bordel et al. 2023; Martín-González et al. 2023a, b).

These findings demonstrate that the native microbiota associated with raw feathers is sufficient to sustain efficient keratin degradation without the need for external inoculum preparation. Moreover, the high proportion of highly keratinolytic strains isolated in this study suggests that this starter-free strategy may be robust across different geographical locations and poultry waste streams, thereby supporting the scalability and broad applicability of the proposed bioprocess.

Nevertheless, the overall results obtained in this study seem to indicate that effective feather hydrolysis cannot be attributed solely to the presence of individual keratinolytic microorganisms, but rather to the establishment of a diverse and dynamic microbial consortium capable of maintaining process performance over extended operational periods. Although keratinase production represents a central functional trait, long-term stability appears to depend on metabolic complementarity among community members, including taxa involved in amino acid assimilation, nitrogen mineralization, acid consumption, and redox balance. The observed microbial succession and persistence of a stable core microbiota suggest that the indigenous feather-associated community self-organized into a functionally integrated consortium, adapted to shifting oxygen regimes and capable of sustaining continuous feather valorization without external intervention.

## Conclusions

A 100-day experiment was conducted in a custom-built reactor specifically engineered for demonstrating that raw chicken feathers can be efficiently valorized through a simple microbial process relying exclusively on native microbiota, water, and mild operating conditions. Instead of manipulating individual operational variables, the process was intentionally operated under minimally controlled conditions, with only temperature and mixing regulated, in order to evaluate whether effective feather depolymerization, nitrogen mineralization and enzyme production could emerge spontaneously from native microbial communities in the absence of sterilization or external inputs.

**Fig. 5:**
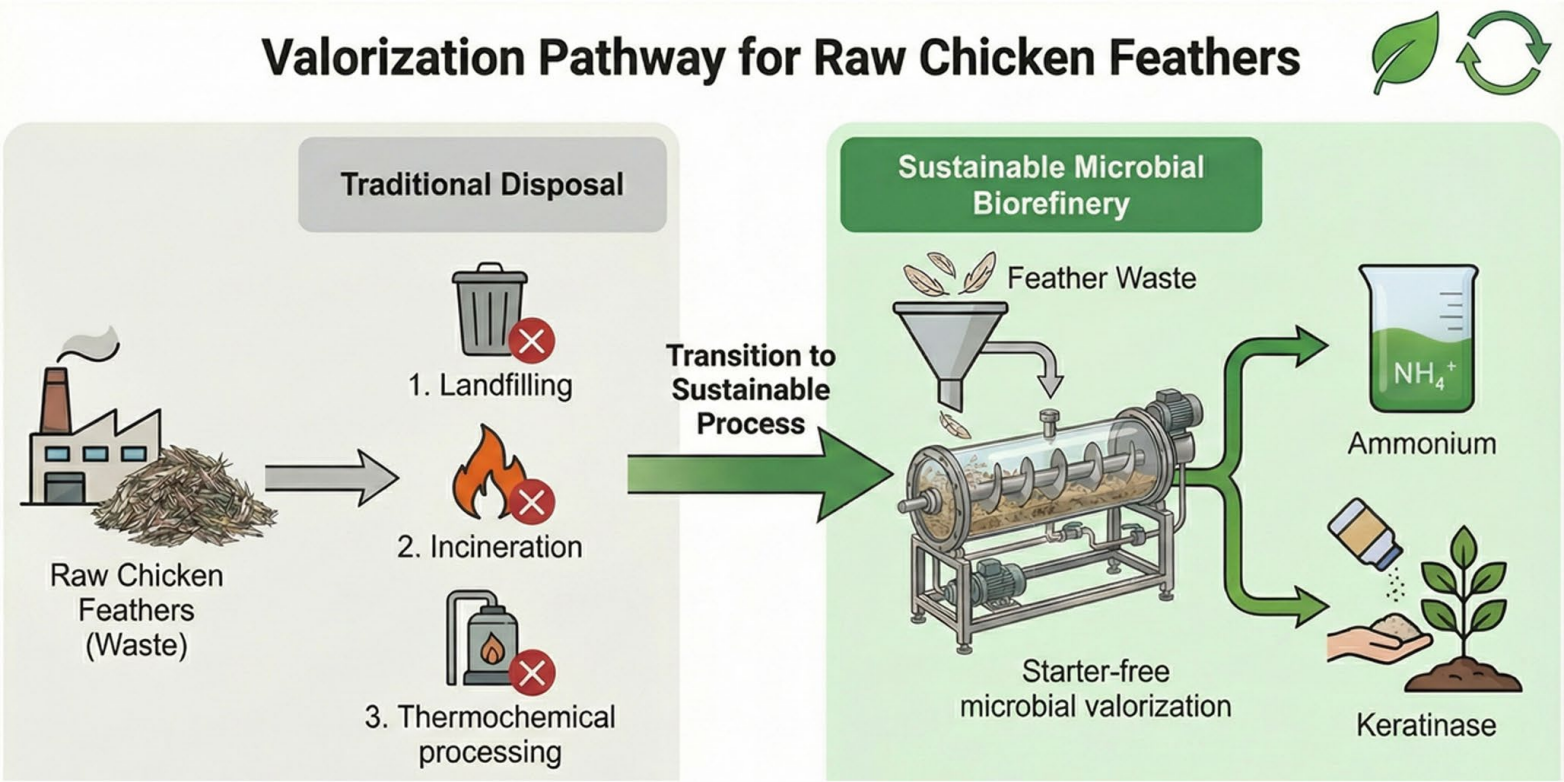
Conceptual representation of the proposed inoculum-free process for converting raw chicken feathers into ammonium and keratinases in a pilot-scale bioreactor. Mechanically untreated feathers are directly fed with tap water, without sterilization, inoculation or nutrient supplementation. Native feather-associated microbiota colonize the keratin substrate and secrete extracellular keratinases, promoting keratin hydrolysis into soluble peptides and amino acids. Subsequent microbial metabolism leads to the accumulation of free ammonium and dissolved carbon species, particularly under oxygen-limited conditions. The process generates an ammonium-rich liquid stream and keratinolytic enzymes with potential applications in agriculture, biotechnology and waste management, offering a scalable and low-energy alternative to conventional feather treatment technologies for sustainable nitrogen and enzyme recovery

Without mechanical pretreatment, external inoculation, nutrient supplementation, or pH control, effective feather degradation was achieved at pilot scale (50 L), yielding high ammonium concentrations and elevated keratinolytic activity (Fig. 5). Ammonium production stabilized at approximately 2 g L⁻¹ under intermittently aerated conditions and increased to nearly 5 g L⁻¹ following the transition to oxygen-limited operation, while keratinolytic activity reached values exceeding 31,000 U mL⁻¹.

Microbial analyses revealed a resilient and self-organized consortium dominated by indigenous microorganisms, characterized by a stable core microbiota and pronounced functional redundancy, as confirmed by the recovery of numerous keratinolytic isolates. The ability of this native community to sustain long-term process performance without external inoculation strongly supports the scalability and robustness of the approach.

In summary, these results highlight the potential of this process for generating ammonium-rich streams and industrially relevant keratinases from poultry waste. It is important to note that the study should be viewed as a systems-level demonstration of industrially relevant performance rather than as a statistically driven optimization exercise. The dataset generated provides a necessary intermediate step between laboratory-scale screening studies and future replication-based pilot campaigns, offering mechanistic insights into community succession and process robustness that can guide subsequent, statistically powered investigations and technology transfer efforts.

## Supplementary Information

Below is the link to the electronic supplementary material.

Supplementary figure 1 Supplementary Figure S1: Photographs illustrating the collection of raw chicken feathers at the poultry slaughterhouse and their immediate transport to the laboratory in a new, unused container to preserve the native microbiota. (PNG 2.47 mb)

Supplementary Material 1

Supplementary figure 2 Supplementary Figure S2: Temporal evolution of ammonium (NH_₄_^⁺^), nitrate (NO_₃_^⁻^), nitrite (NO_₂_^⁻^), dissolved organic carbon (DOC), dissolved inorganic carbon (DIC) and proline concentrations (g L^⁻¹^) in the reactor supernatant during the 100-day pilot-scale feather valorization process. The x-axis represents the incubation time, including the intermittently aerated phase (days 0–50) and the oxygen-limited phase (days 50–100). (PNG 161 kb)

Supplementary Material 2

Supplementary figure 3 Supplementary Figure S3: Temporal evolution of bacterial alpha diversity throughout the experiment assessed using the Shannon diversity index. (PNG 71.0 kb)

Supplementary Material 3

Supplementary figure 4 Supplementary Figure S4: Temporal dynamics of the 14 core microbiota genera identified during the pilot-scale feather valorization process. (PNG 506 kb)

Supplementary Material 4

Supplementary figure 5 Supplementary Figure S5: Temporal dynamics of the three core microbiota species identified during the pilot-scale feather valorization process. (PNG 225 kb)

Supplementary Material 5

Supplementary Material 6. Supplementary Figure S6: Growth and keratinase activity of the 132 bacterial isolates recovered during the fourteen-week experimental period. Error bars represent standard deviations calculated from three independent biological replicates.

Supplementary Material 7. Supplementary Table S1: Comprehensive list of the 132 bacterial isolates recovered over the fourteen-week operational period, including sampling time, selective agar medium, dilution factor, strain code and maximum keratinase activity measured in vitro.

Supplementary Material 8. Supplementary Video S1: Video footage showing the pilot-scale horizontal bioreactor during start-up, including the internal agitation and the distribution of feathers within the liquid phase.

## Data Availability

The sequencing data generated in this study are not publicly deposited in a repository but are available from the corresponding author upon reasonable request.
