# Supplementary figures and images for: Pilot-scale inoculum-free valorization of raw chicken feathers: ammonium recovery, keratinase production and community dynamics
Source: World J Microbiol Biotechnol. 2026 May 25;42(6):295. doi: 10.1007/s11274-026-04976-0 (PMC13201326; doi:10.1007/s11274-026-04976-0)

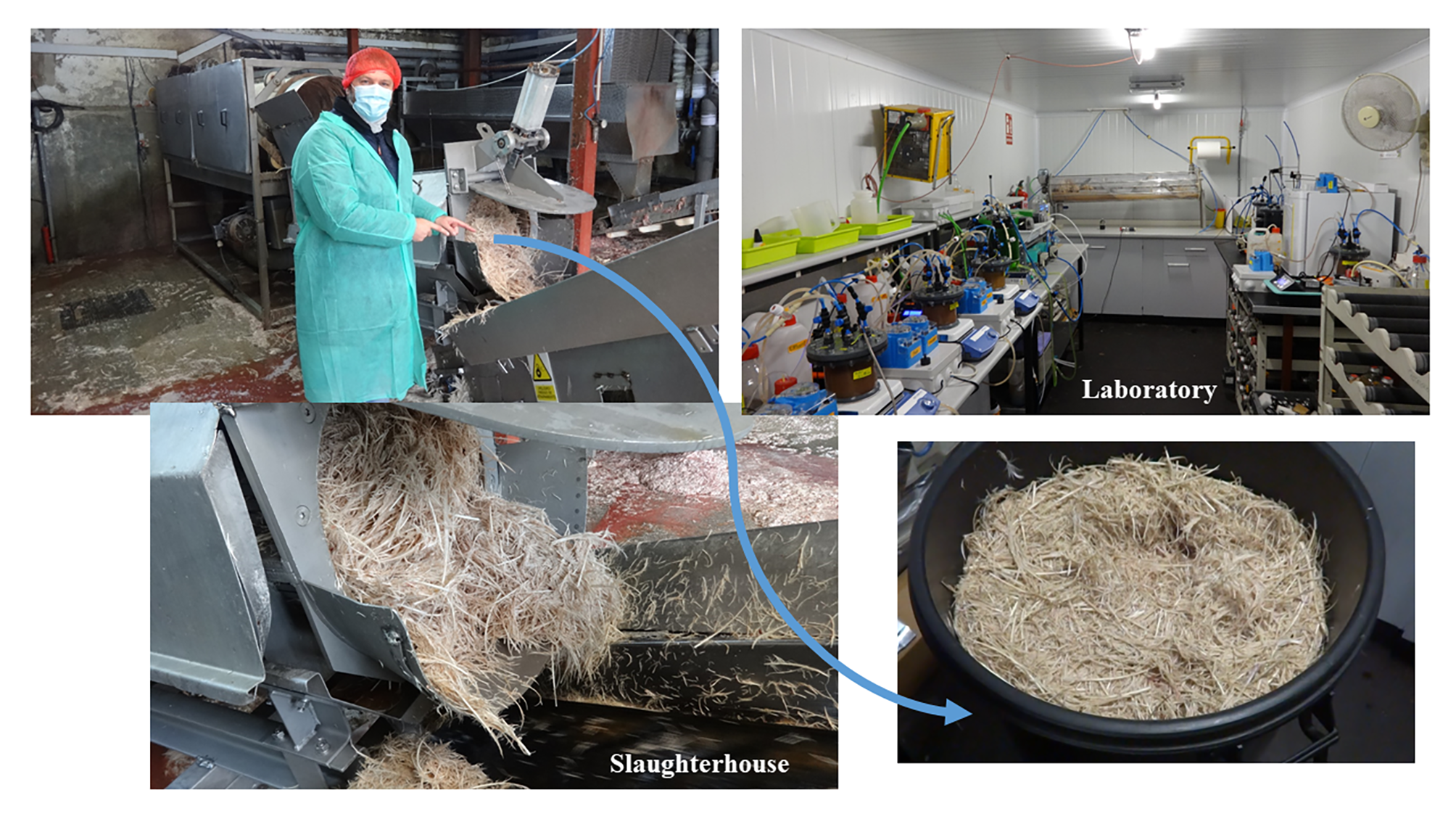

Supplement: Supplementary file 1 — Supplementary Figure S1: Photographs illustrating the collection of raw chicken feathers at the poultry slaughterhouse and their immediate transport to the laboratory in a new, unused container to preserve the native microbiota. (PNG 2.47 mb) [file 11274_2026_4976_Fig6_ESM.png]

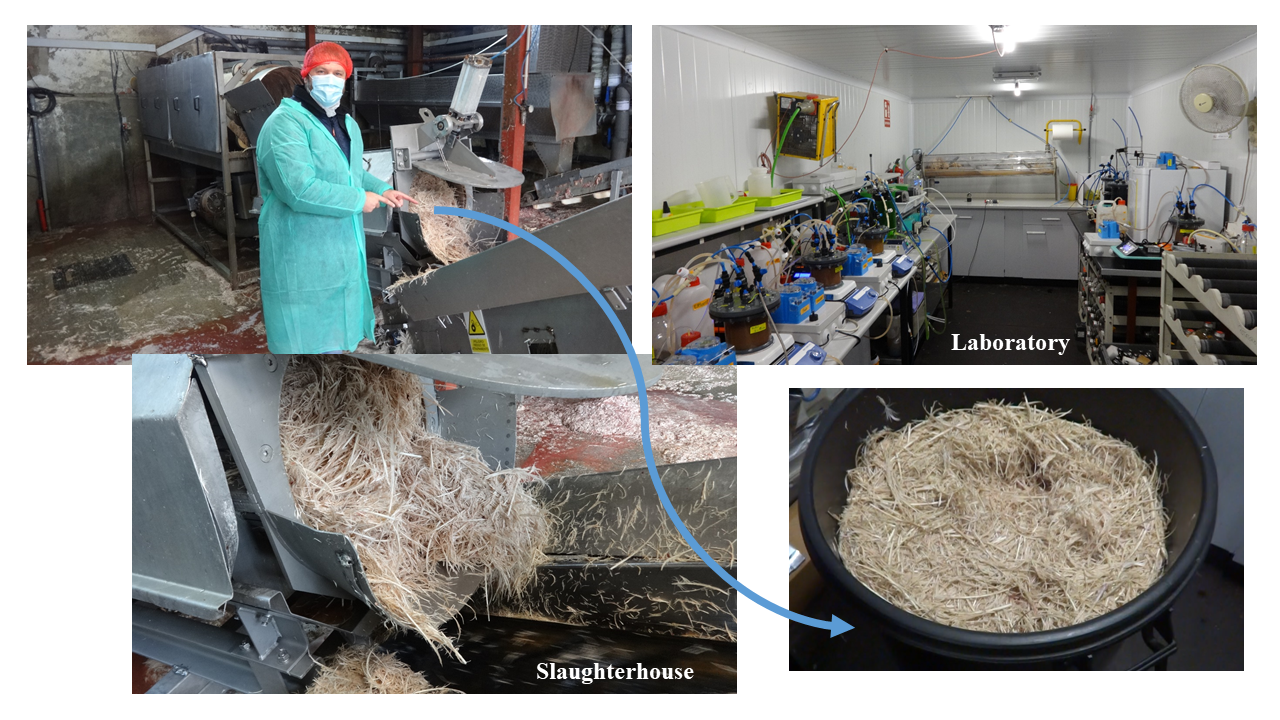

Supplement: Supplementary file 2 — Supplementary Material 1 [file 11274_2026_4976_MOESM1_ESM.tif]

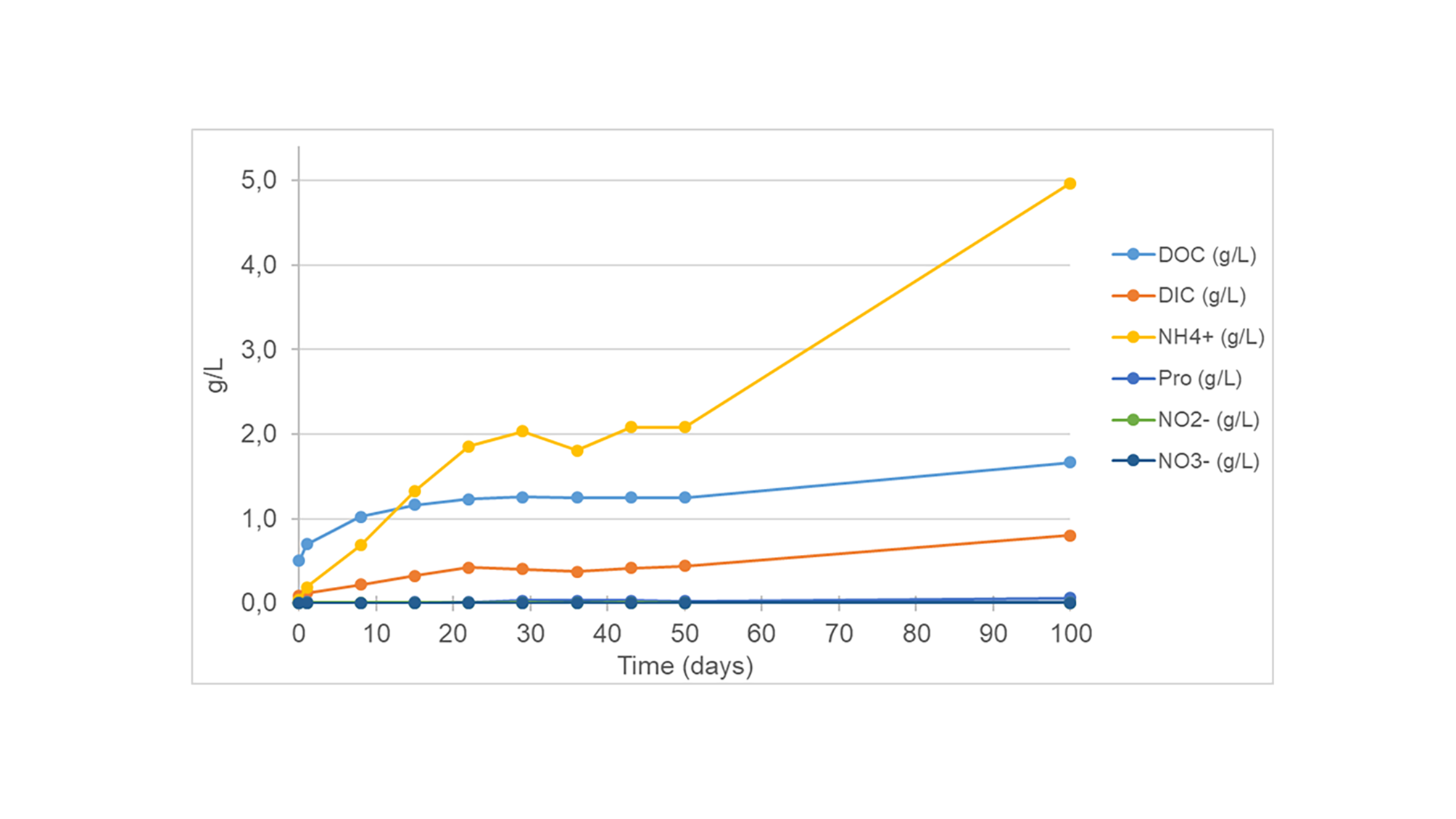

Supplement: Supplementary file 3 — Supplementary Figure S2: Temporal evolution of ammonium (NH₄⁺), nitrate (NO₃⁻), nitrite (NO₂⁻), dissolved organic carbon (DOC), dissolved inorganic carbon (DIC) and proline concentrations (g L⁻¹) in the reactor supernatant during the 100-day pilot-scale feather valorization process. The x-axis represents the incubation time, including the intermittently aerated phase (days 0–50) and the oxygen-limited phase (days 50–100). (PNG 161 kb) [file 11274_2026_4976_Fig7_ESM.png]

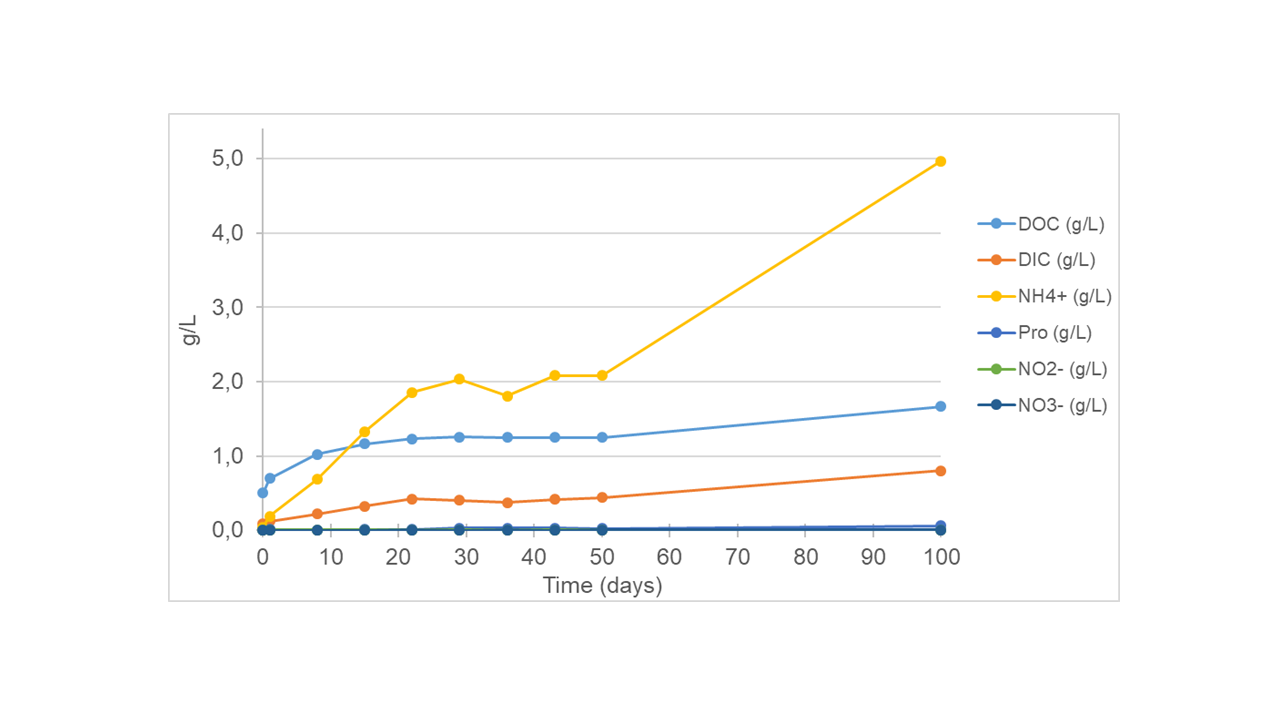

Supplement: Supplementary file 4 — Supplementary Material 2 [file 11274_2026_4976_MOESM2_ESM.tif]

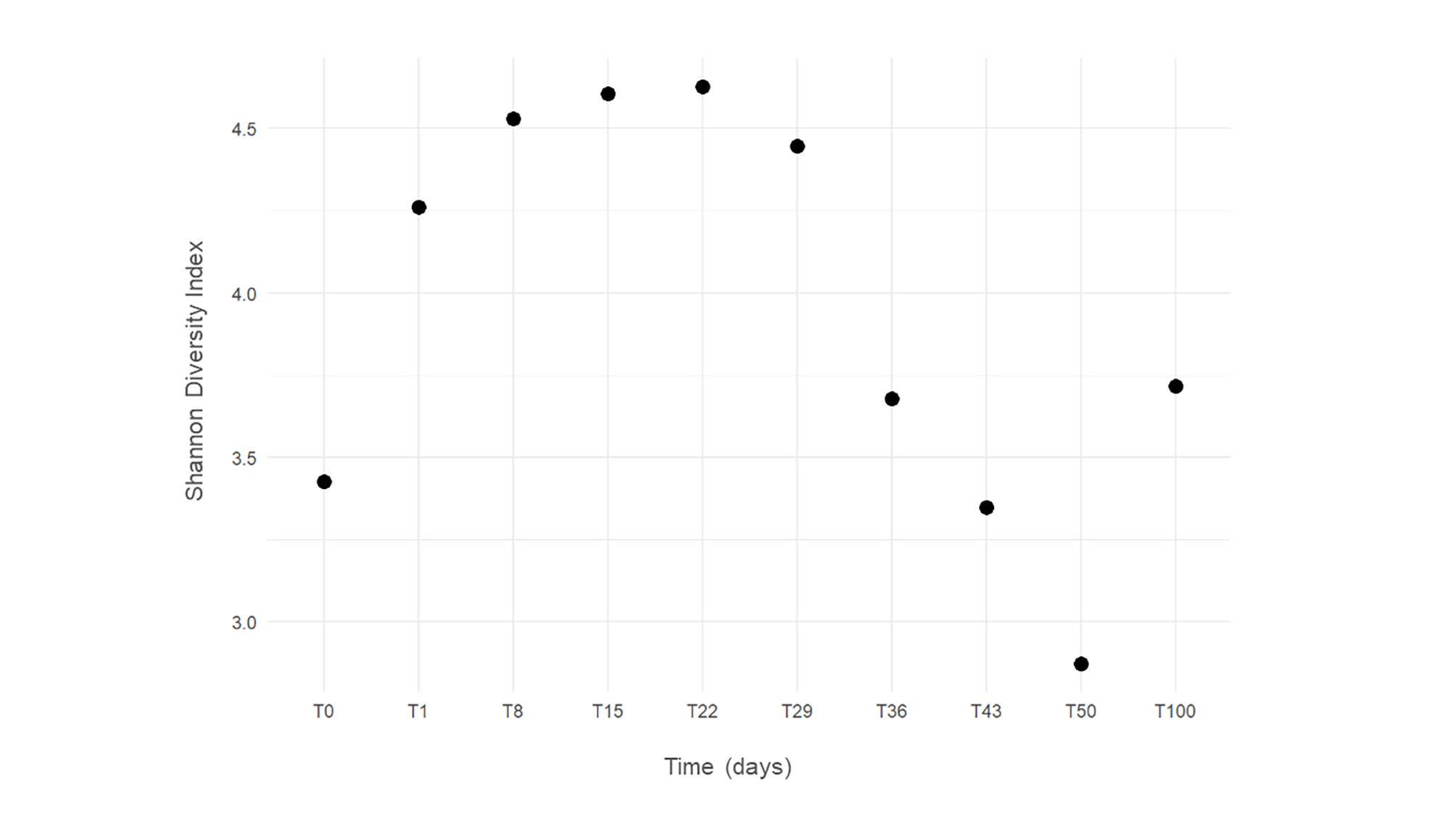

Supplement: Supplementary file 5 — Supplementary Figure S3: Temporal evolution of bacterial alpha diversity throughout the experiment assessed using the Shannon diversity index. (PNG 71.0 kb) [file 11274_2026_4976_Fig8_ESM.png]

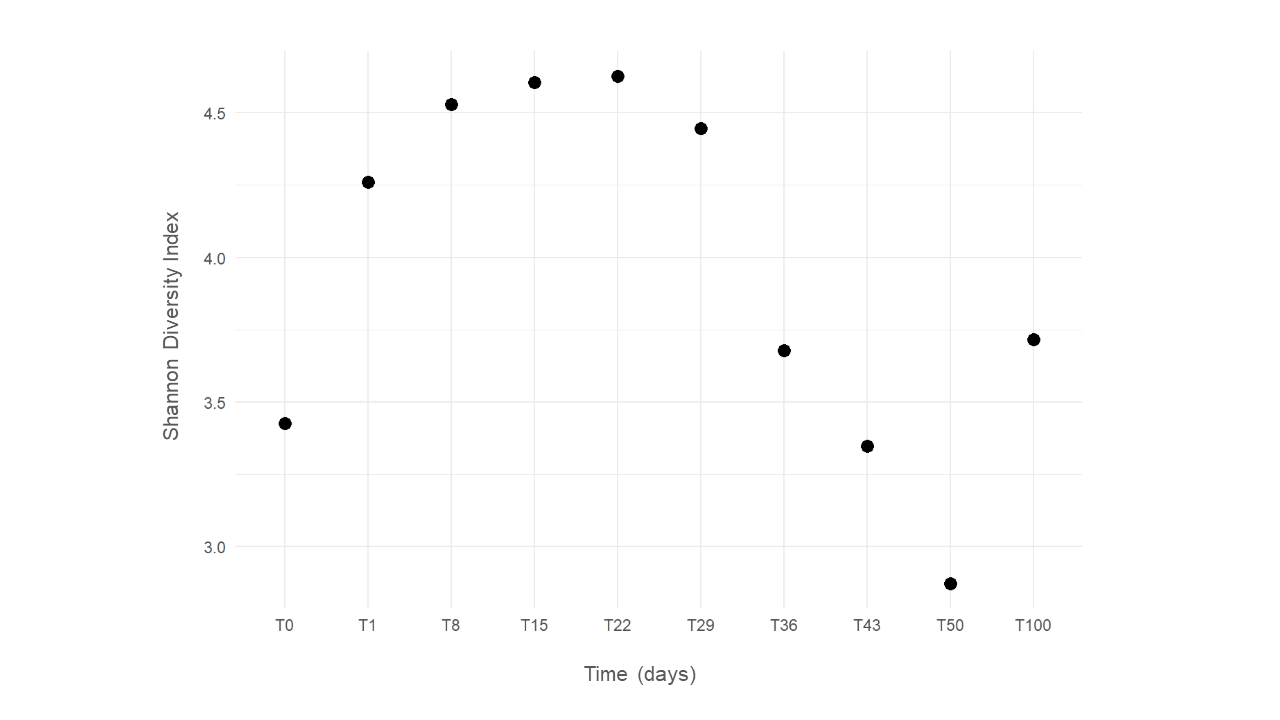

Supplement: Supplementary file 6 — Supplementary Material 3 [file 11274_2026_4976_MOESM3_ESM.tif]

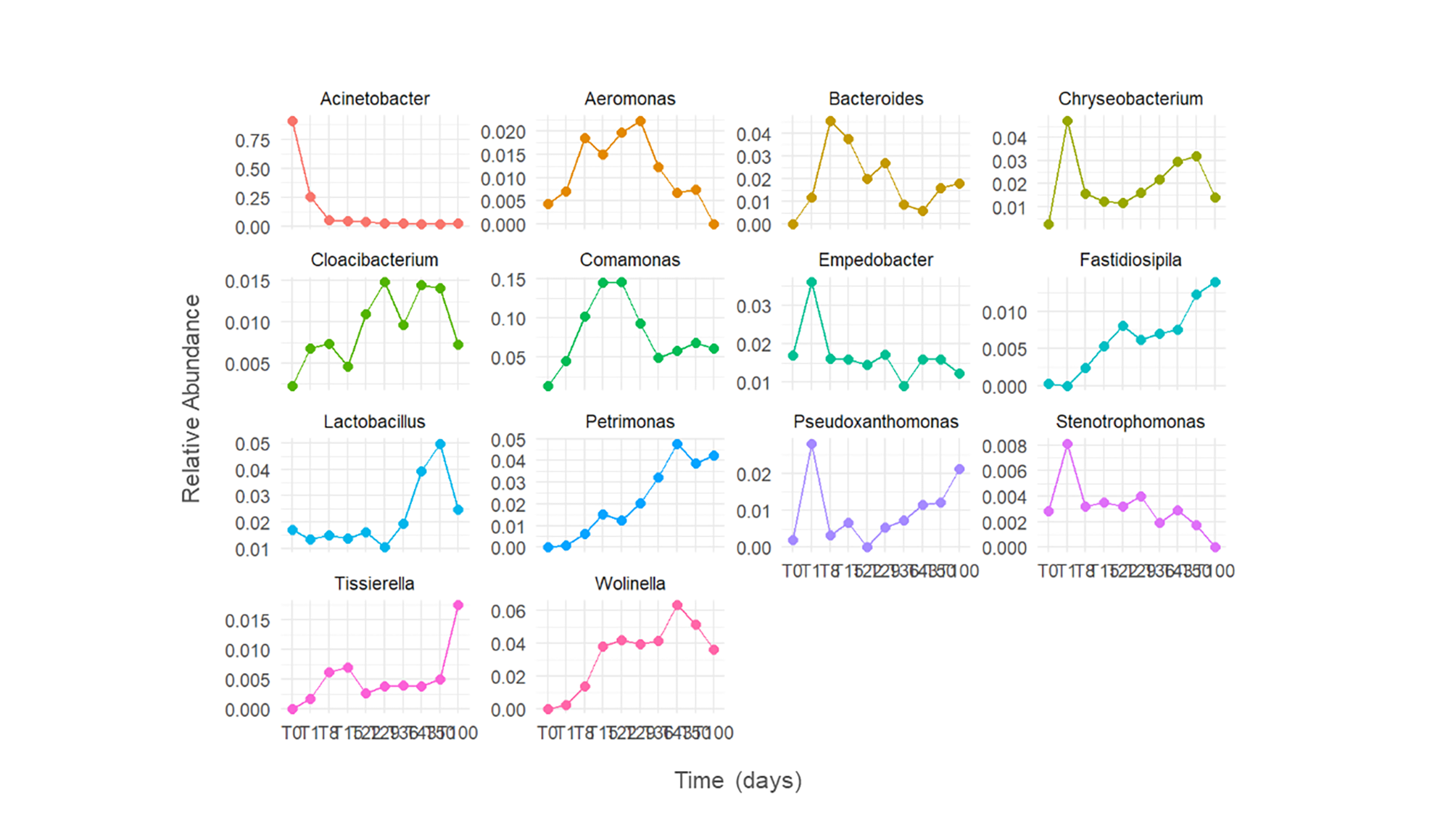

Supplement: Supplementary file 7 — Supplementary Figure S4: Temporal dynamics of the 14 core microbiota genera identified during the pilot-scale feather valorization process. (PNG 506 kb) [file 11274_2026_4976_Fig9_ESM.png]

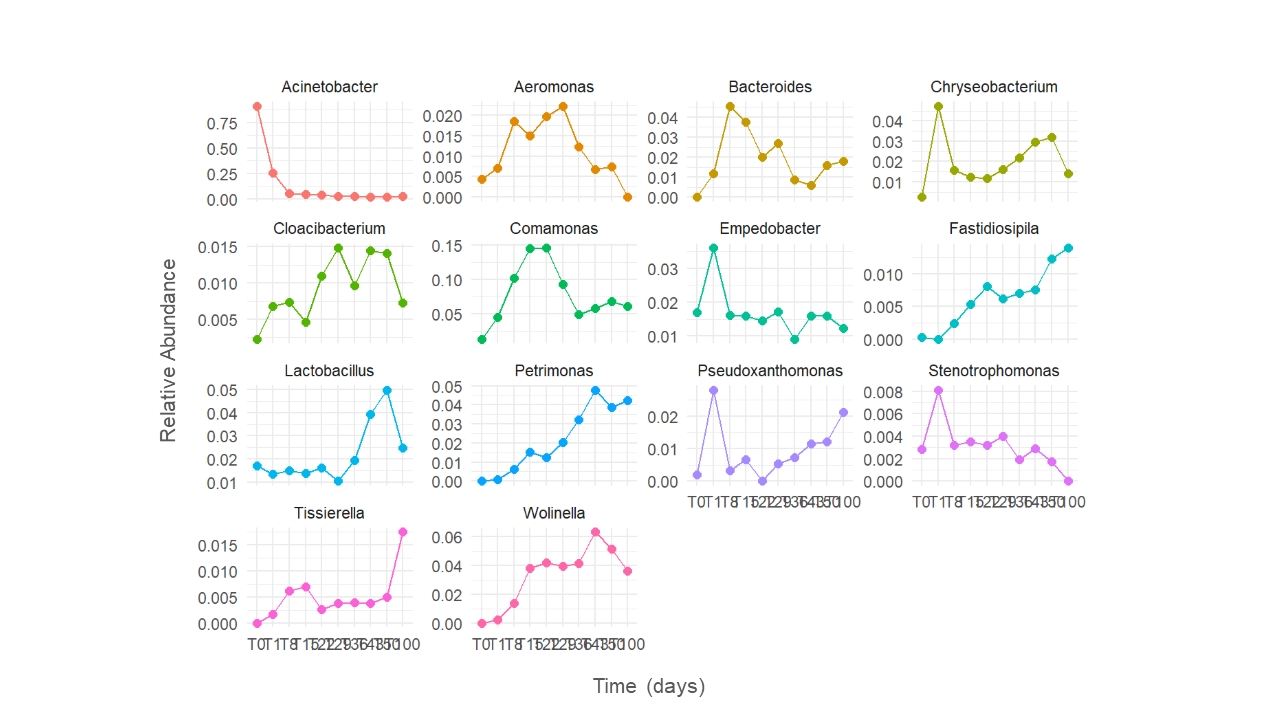

Supplement: Supplementary file 8 — Supplementary Material 4 [file 11274_2026_4976_MOESM4_ESM.tif]

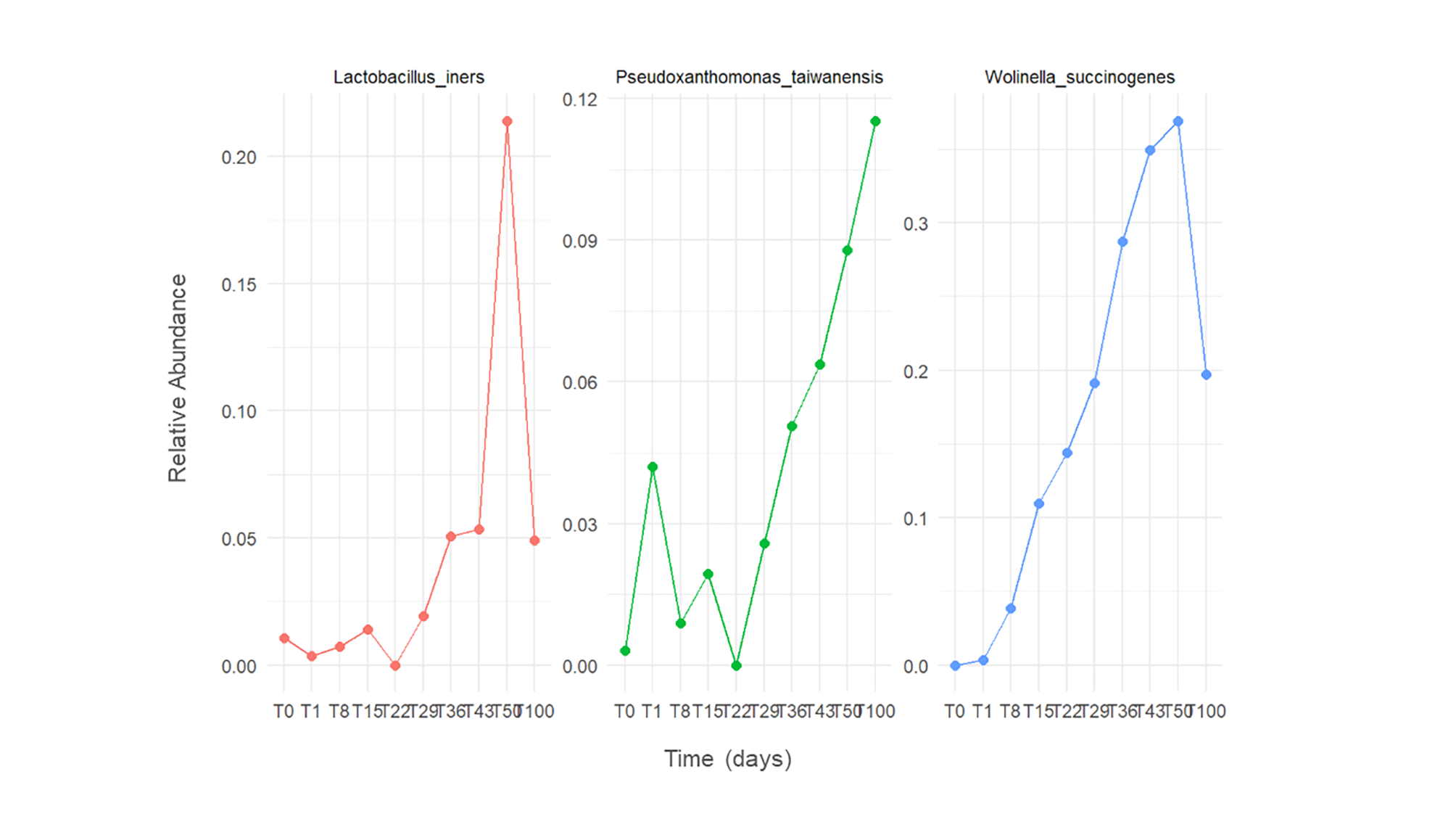

Supplement: Supplementary file 9 — Supplementary Figure S5: Temporal dynamics of the three core microbiota species identified during the pilot-scale feather valorization process. (PNG 225 kb) [file 11274_2026_4976_Fig10_ESM.png]

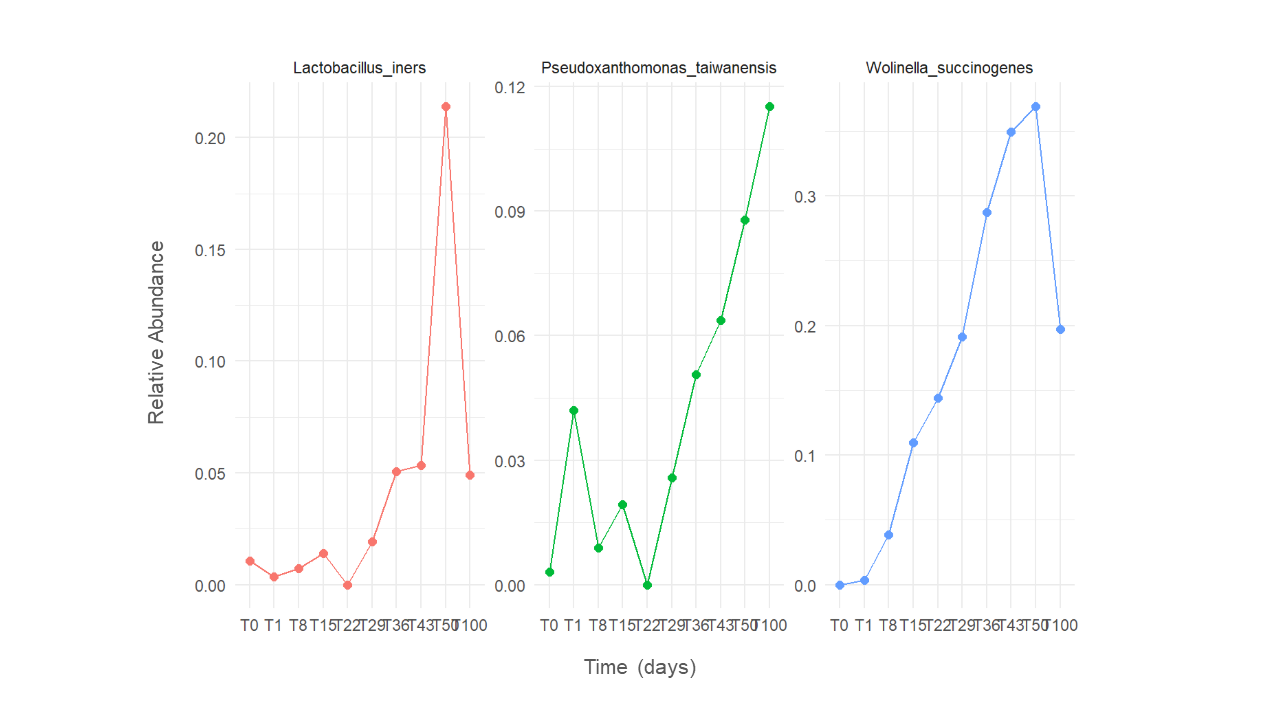

Supplement: Supplementary file 10 — Supplementary Material 5 [file 11274_2026_4976_MOESM5_ESM.tif]

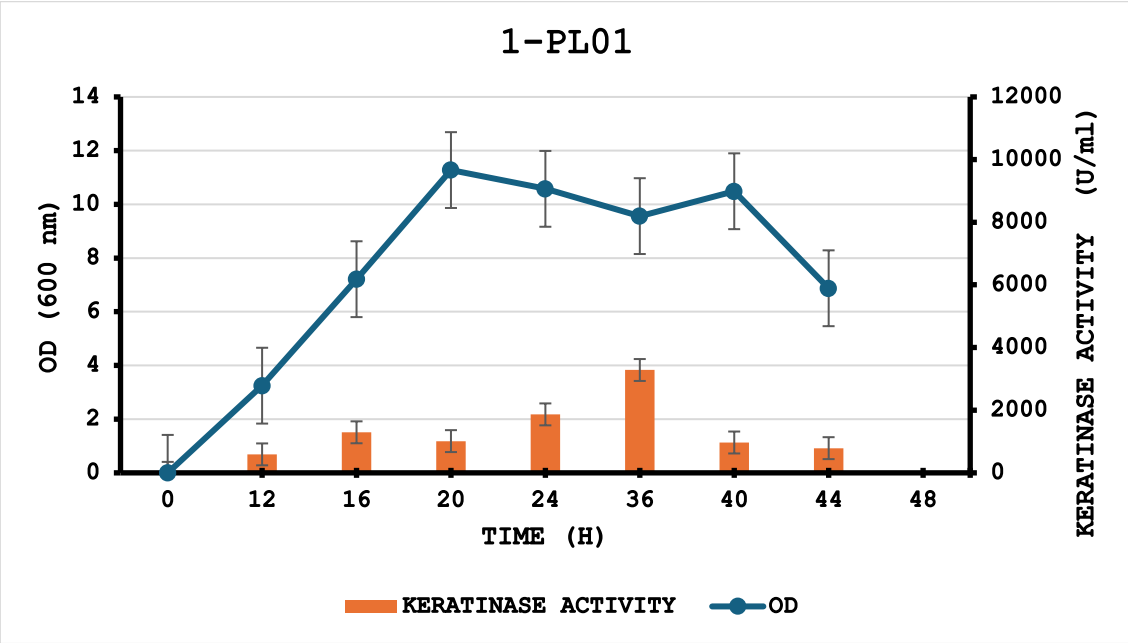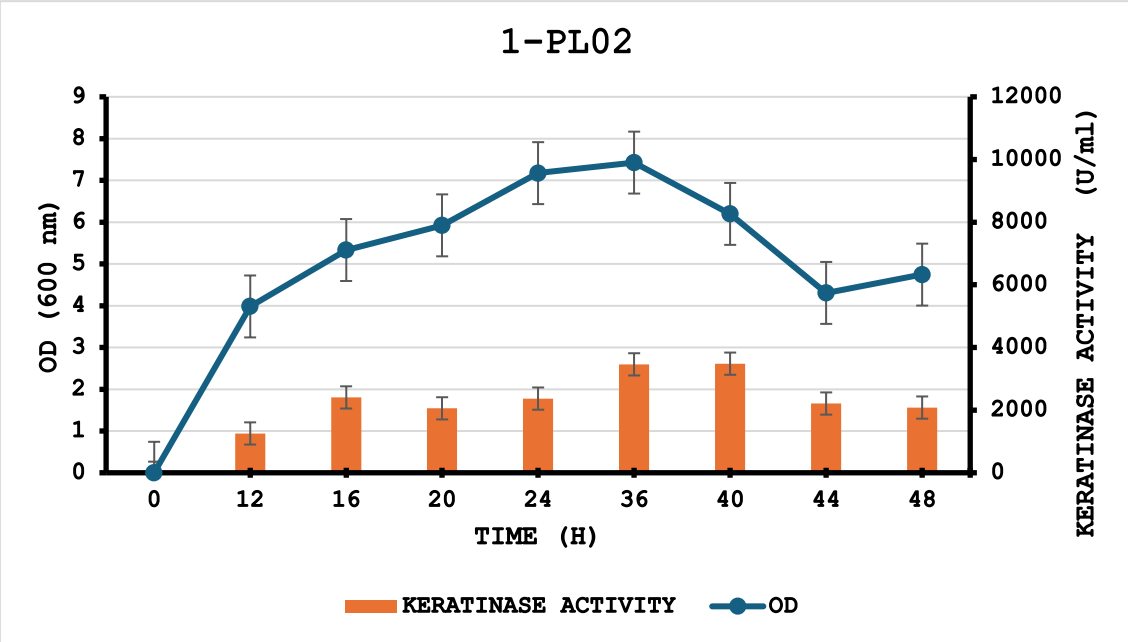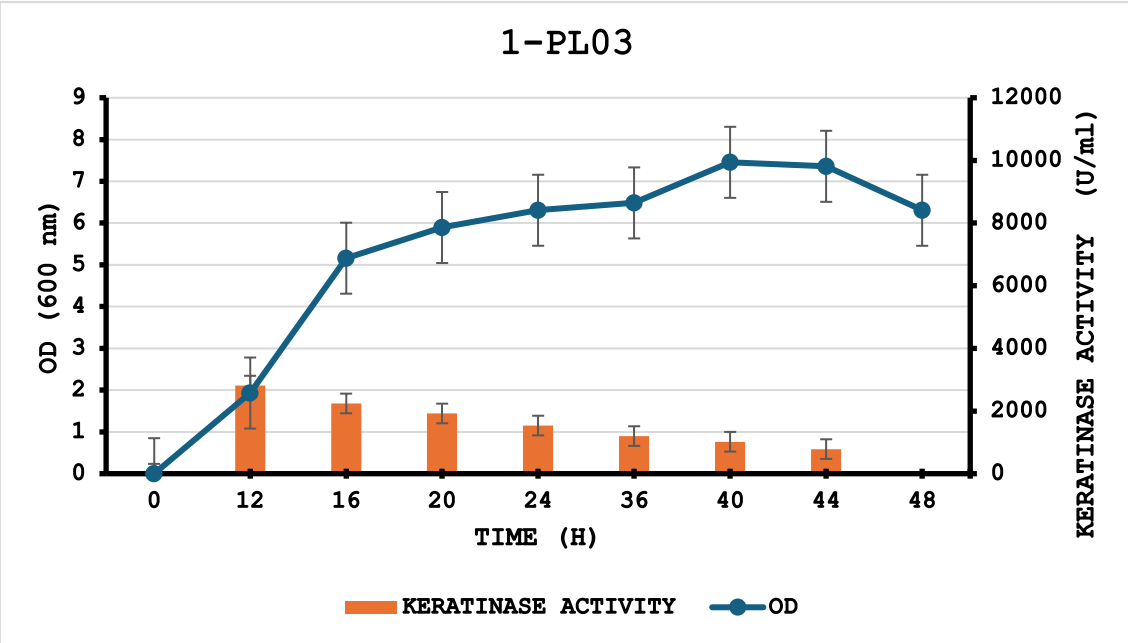

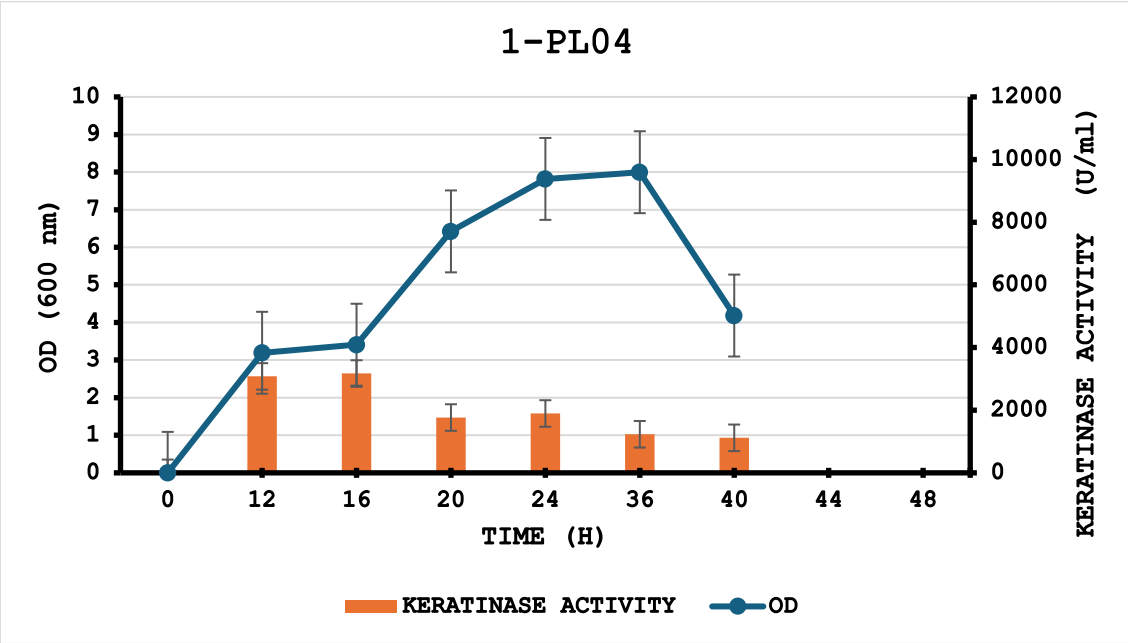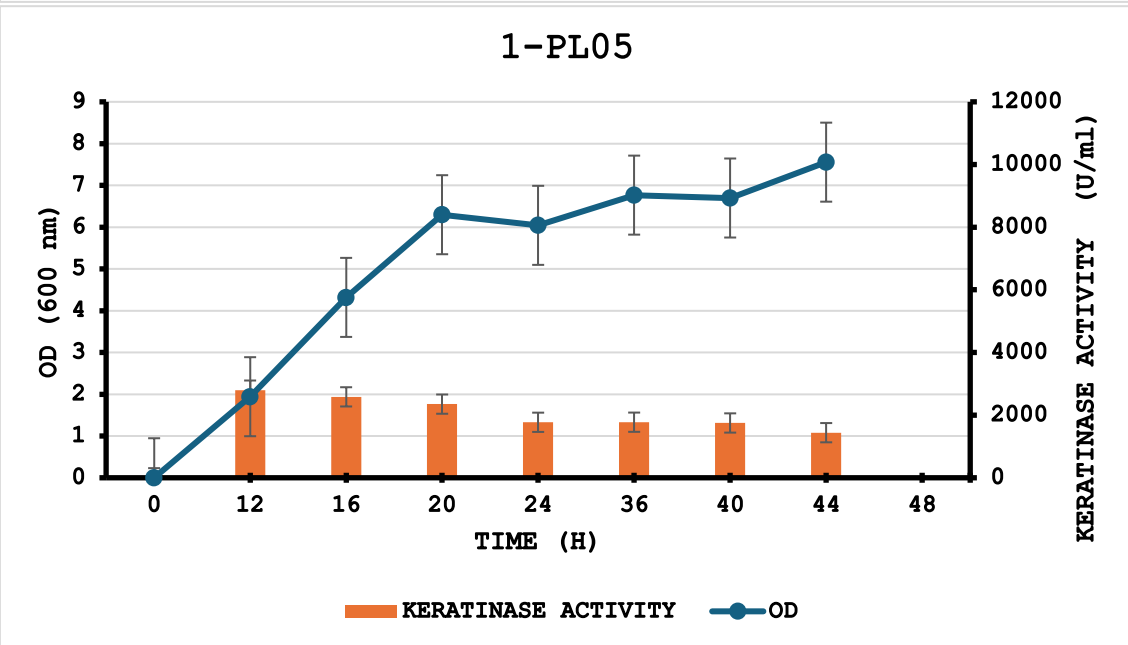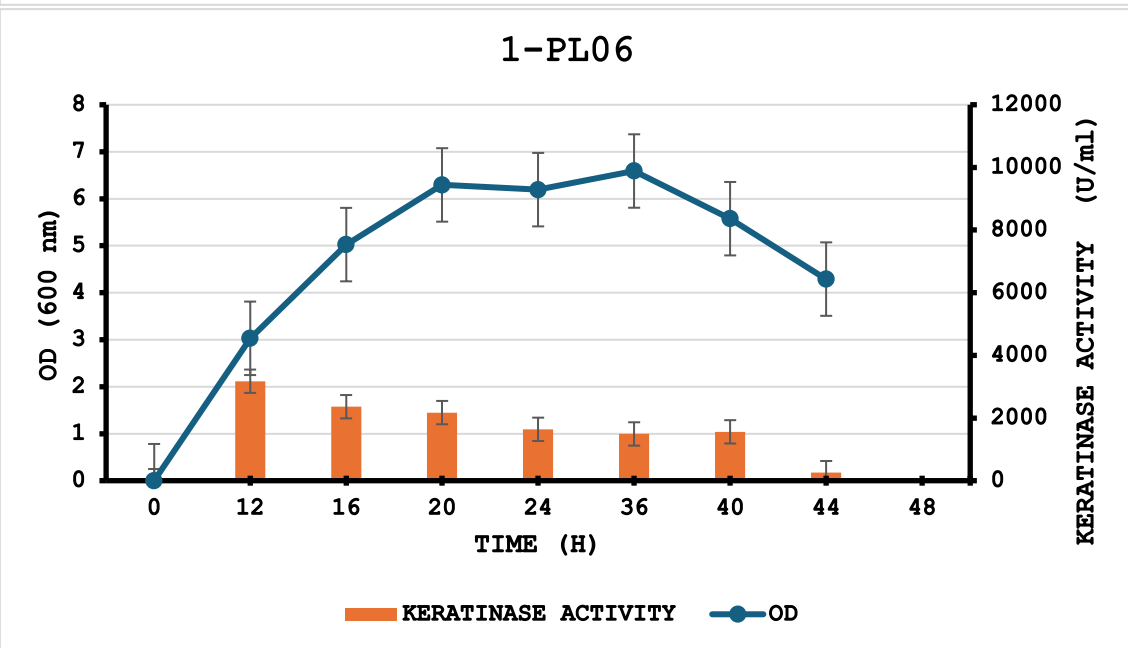

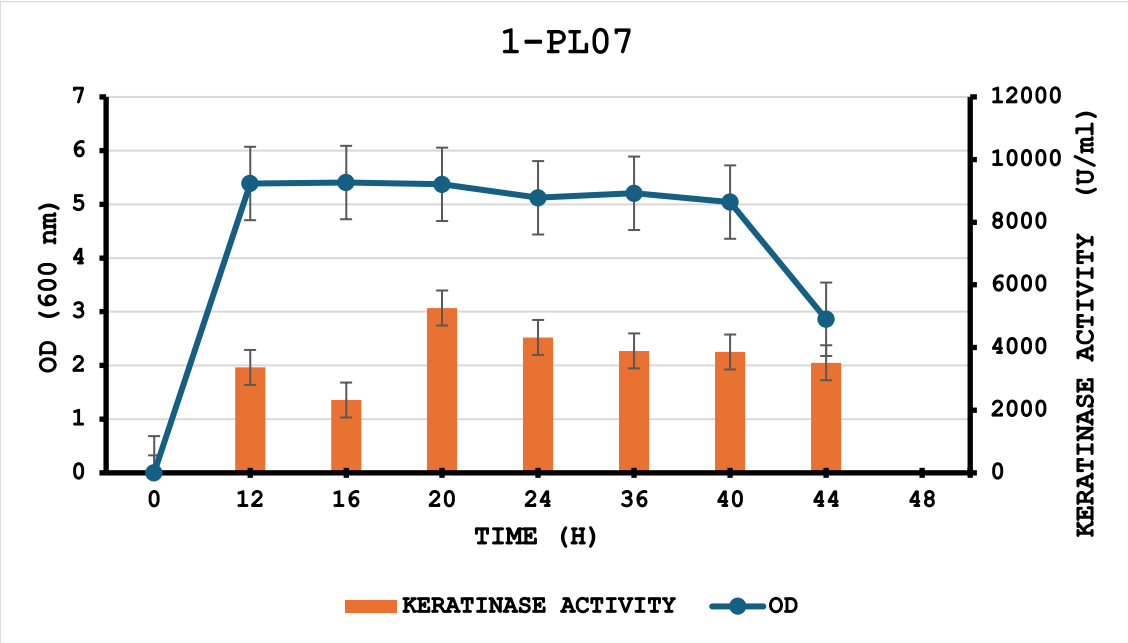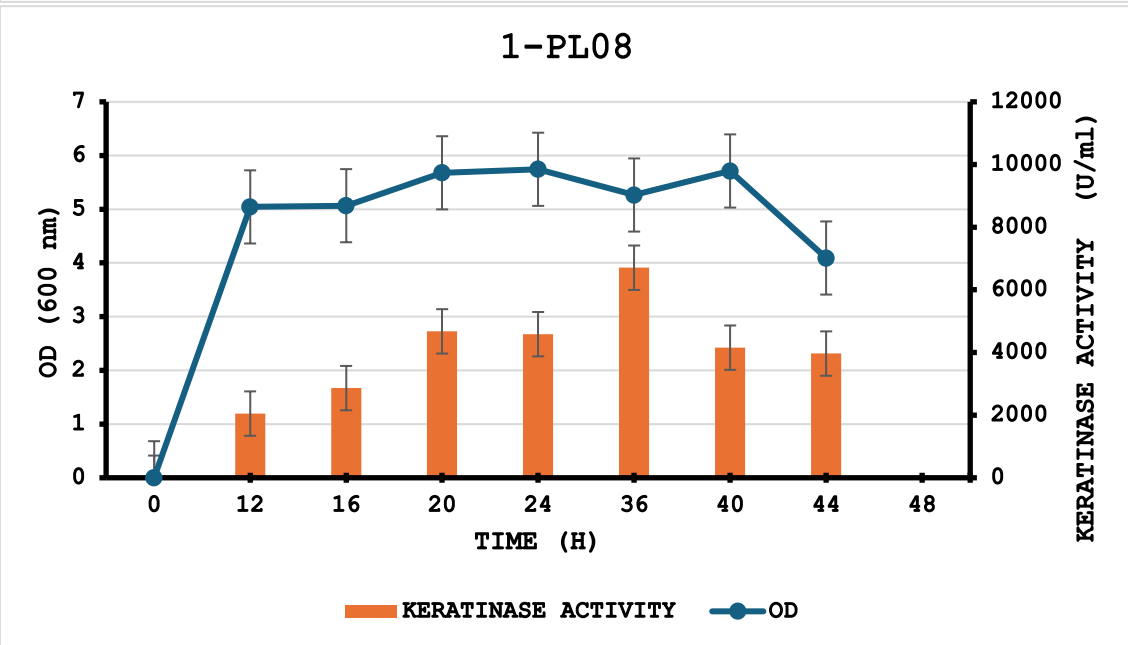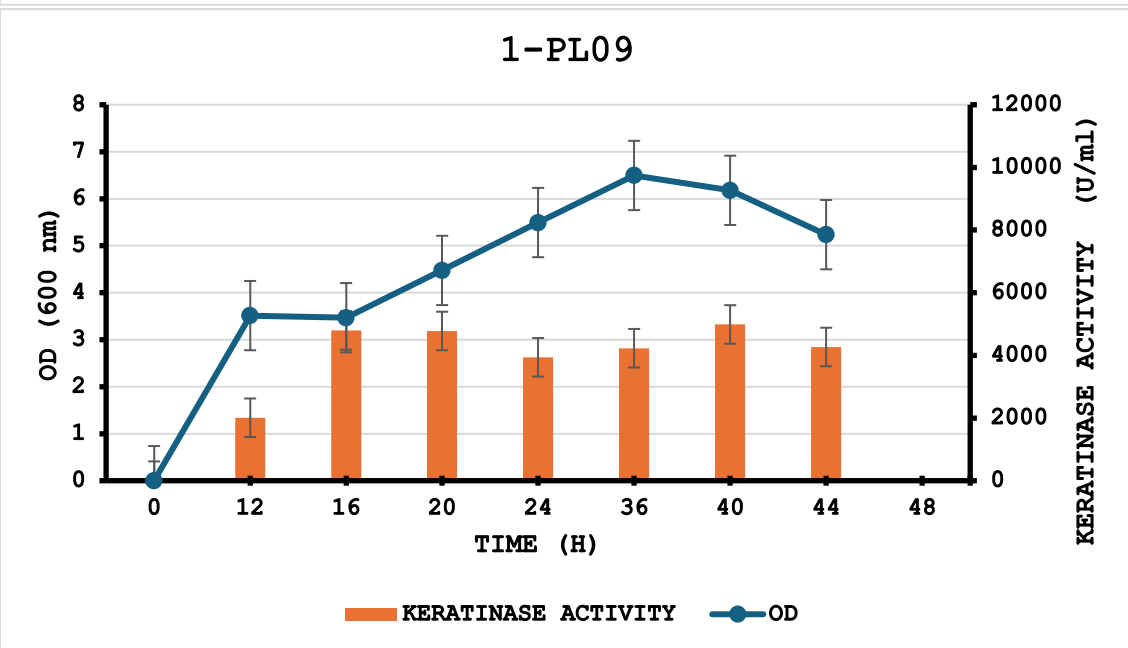

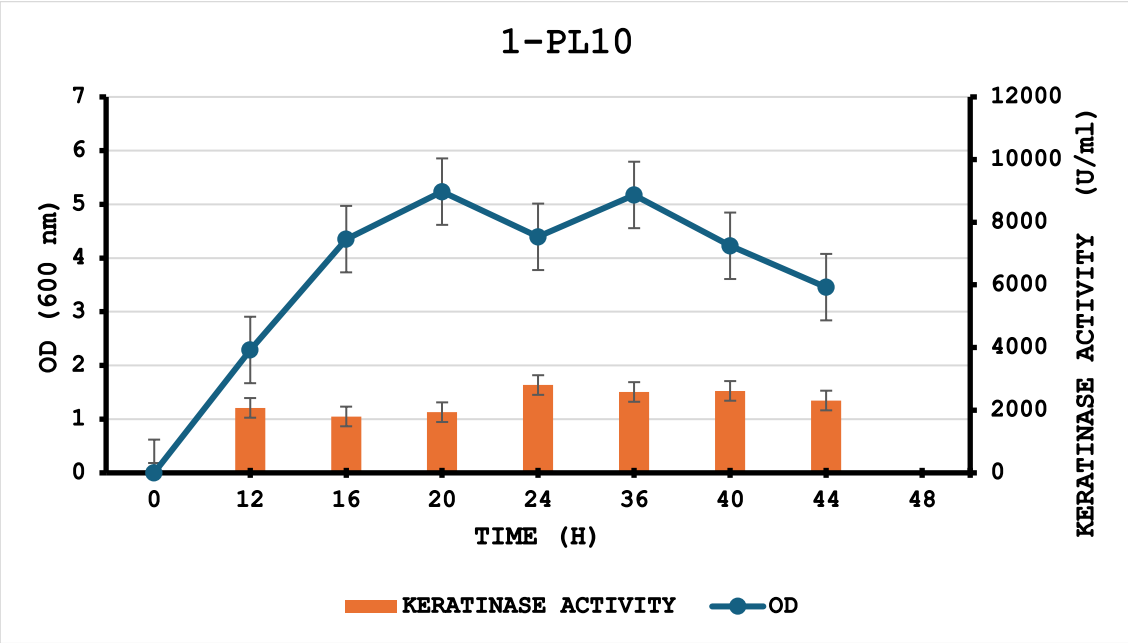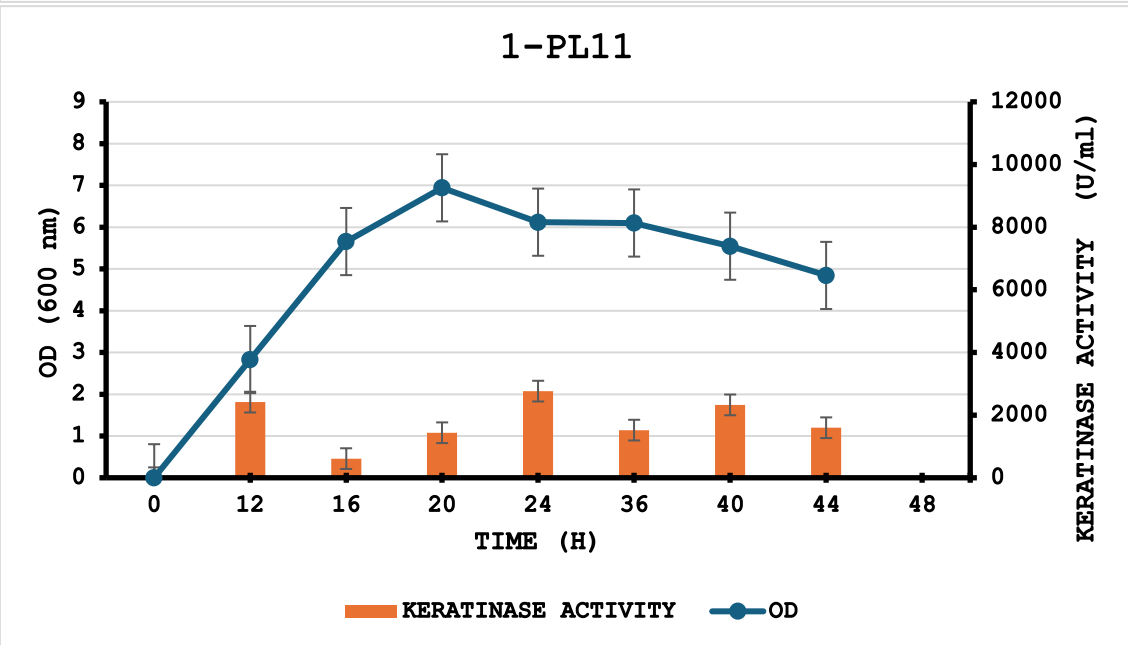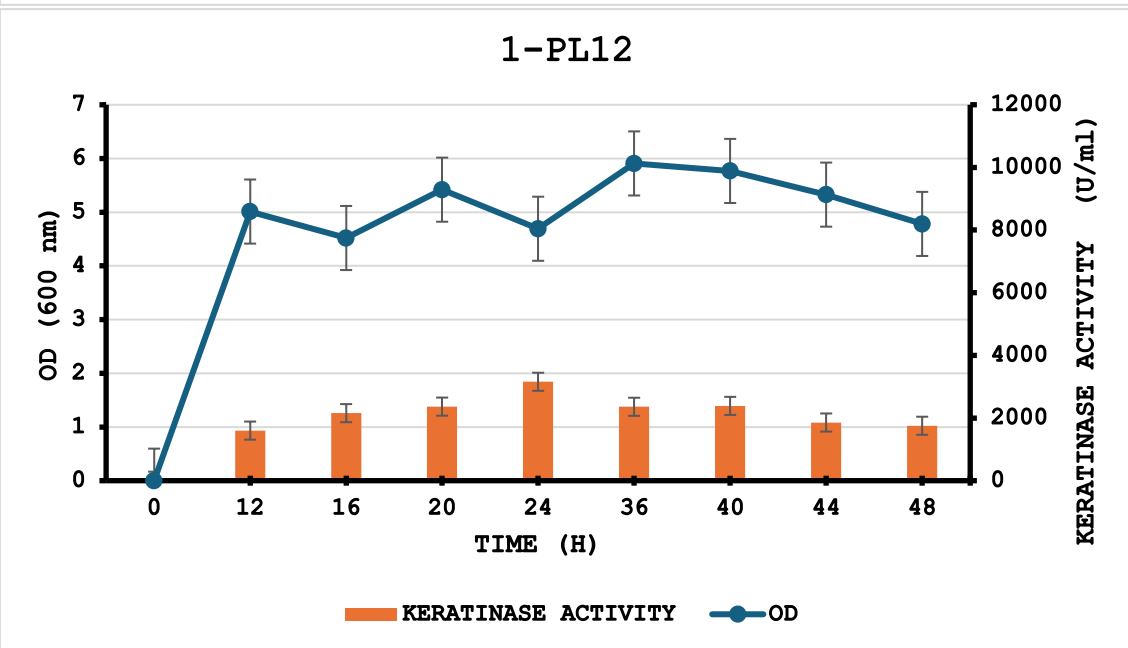

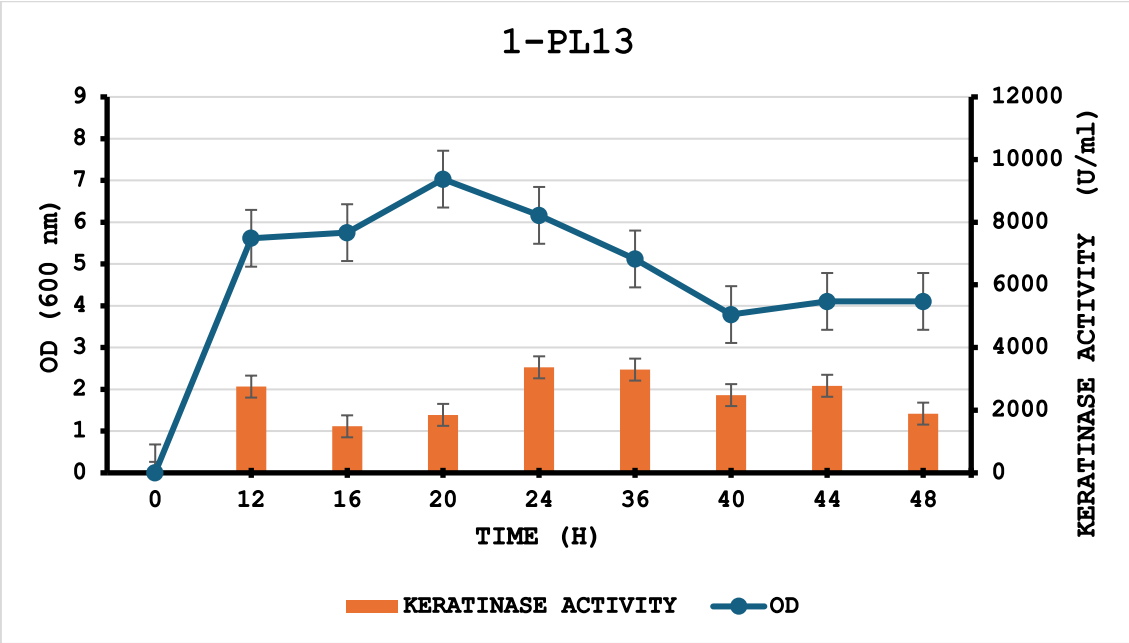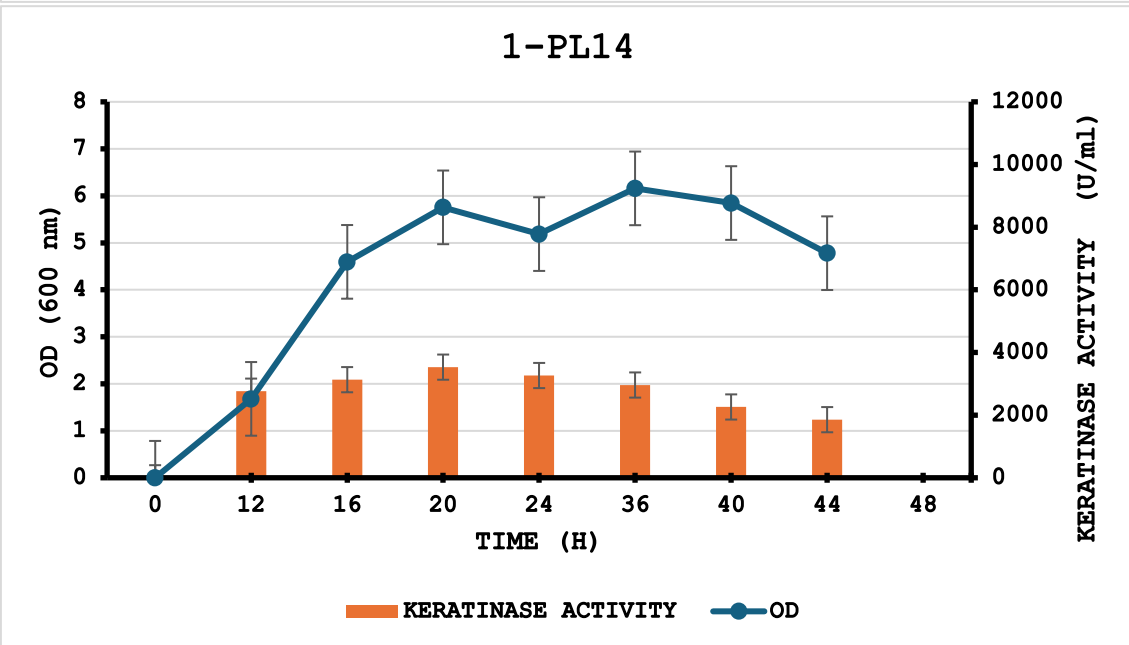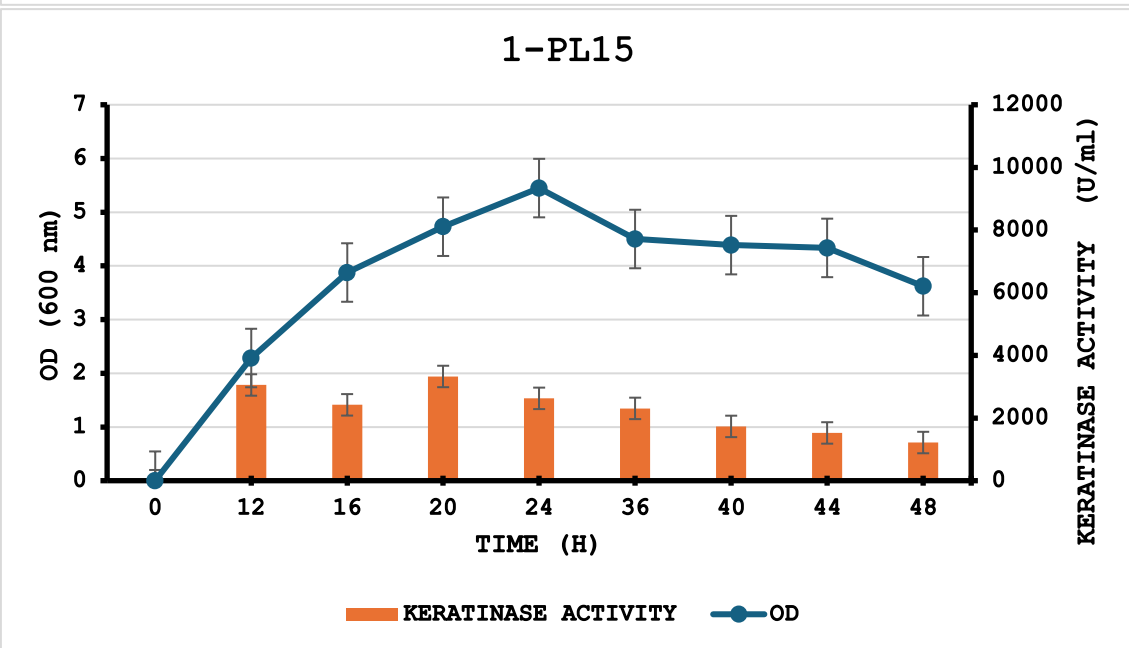

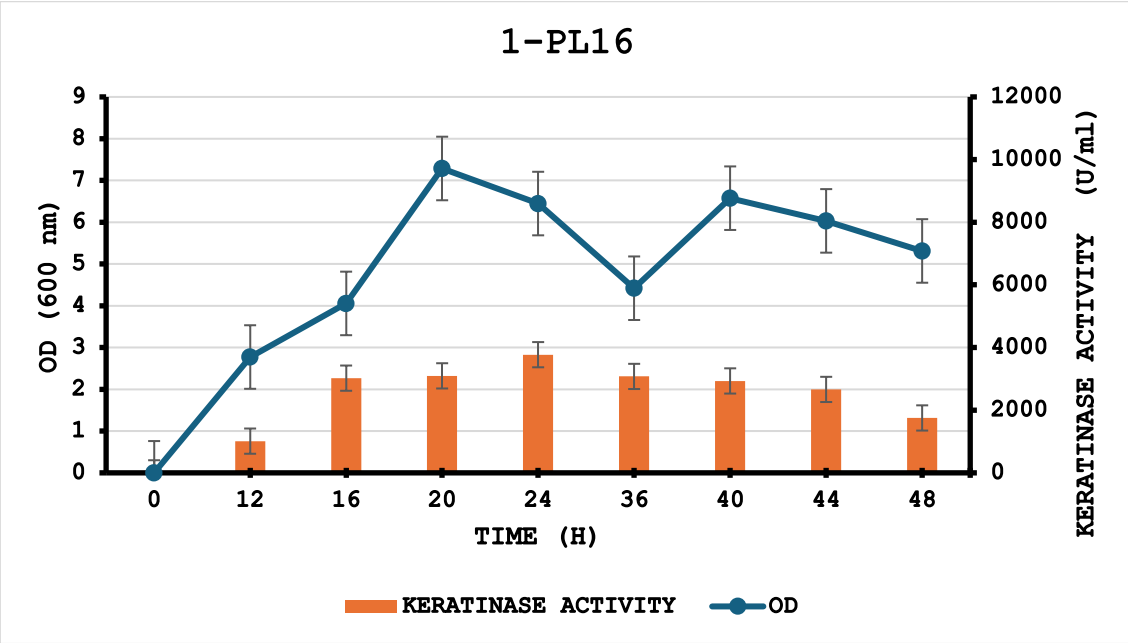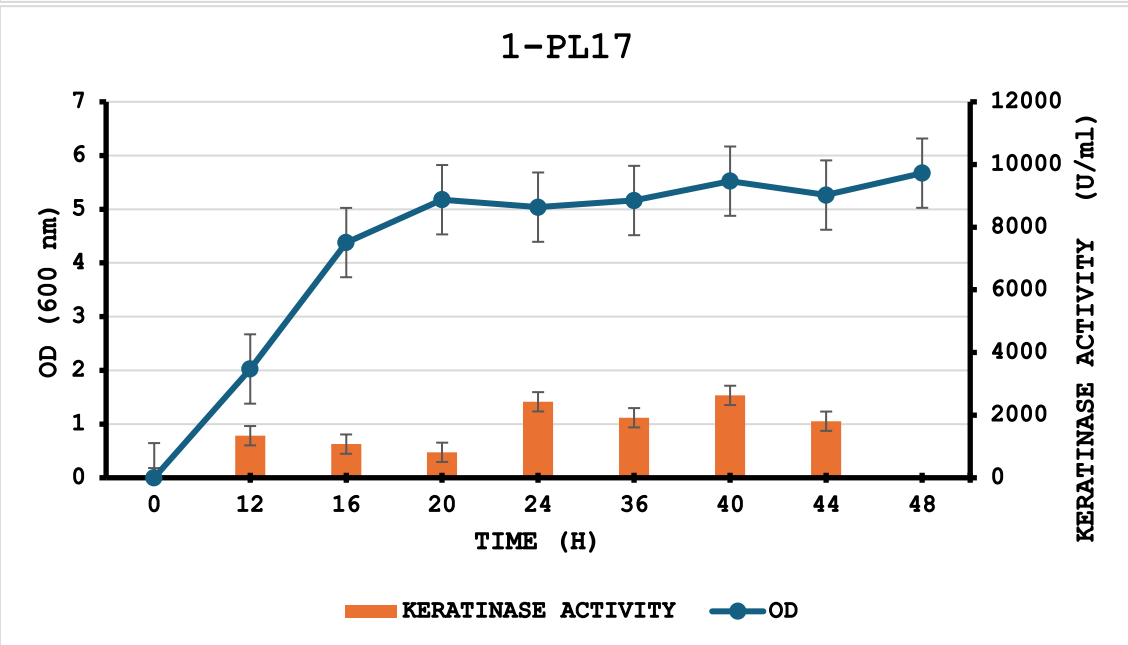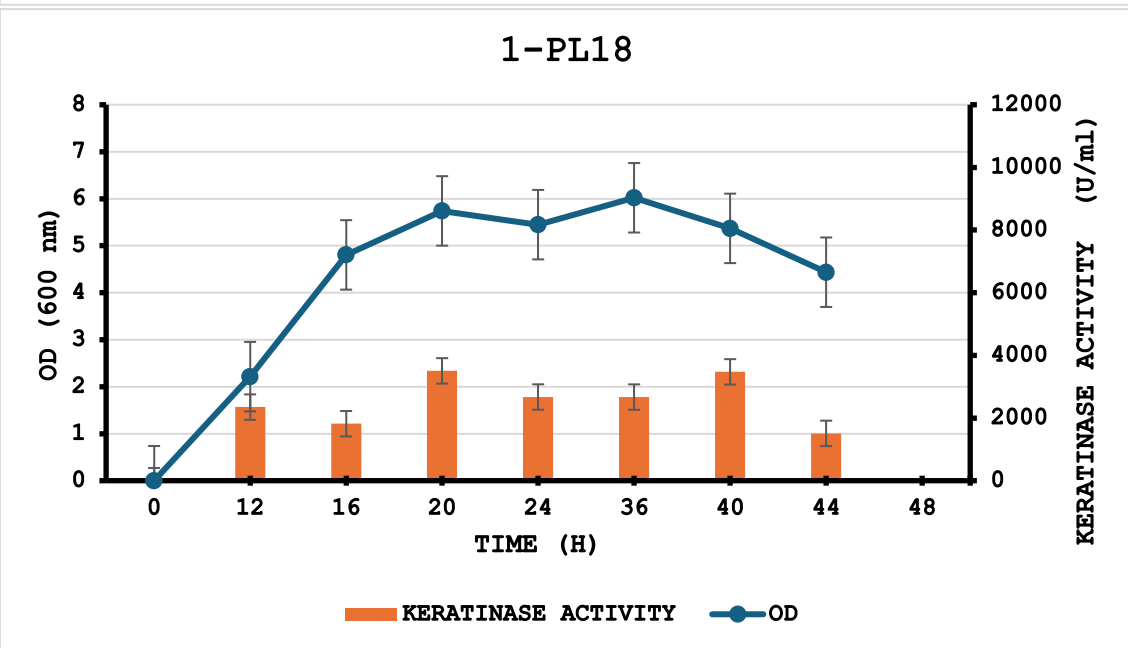

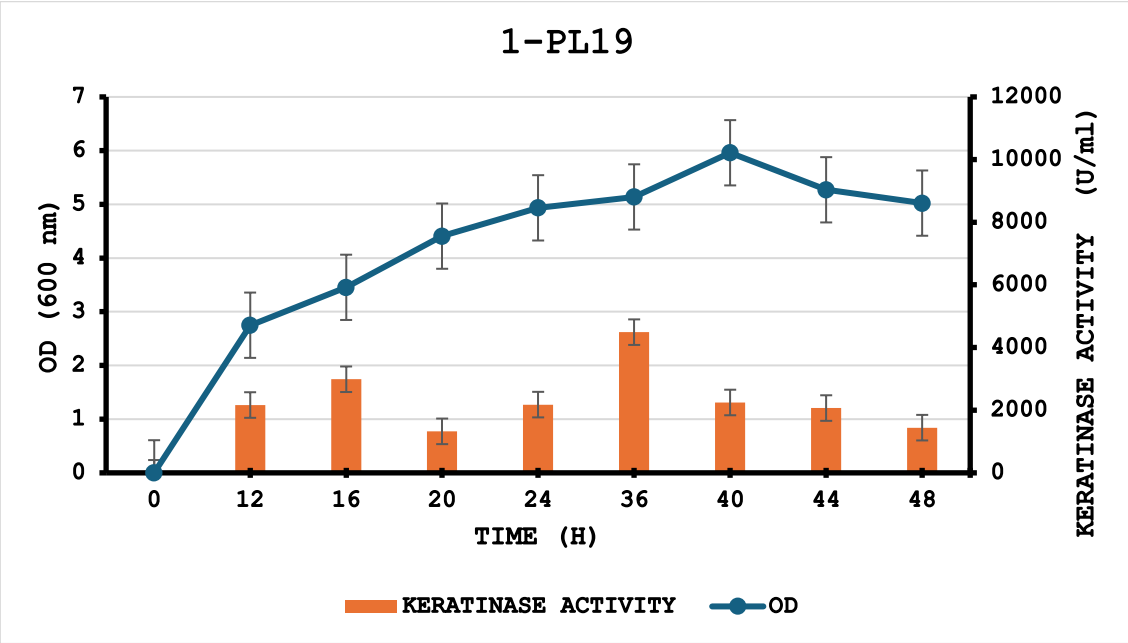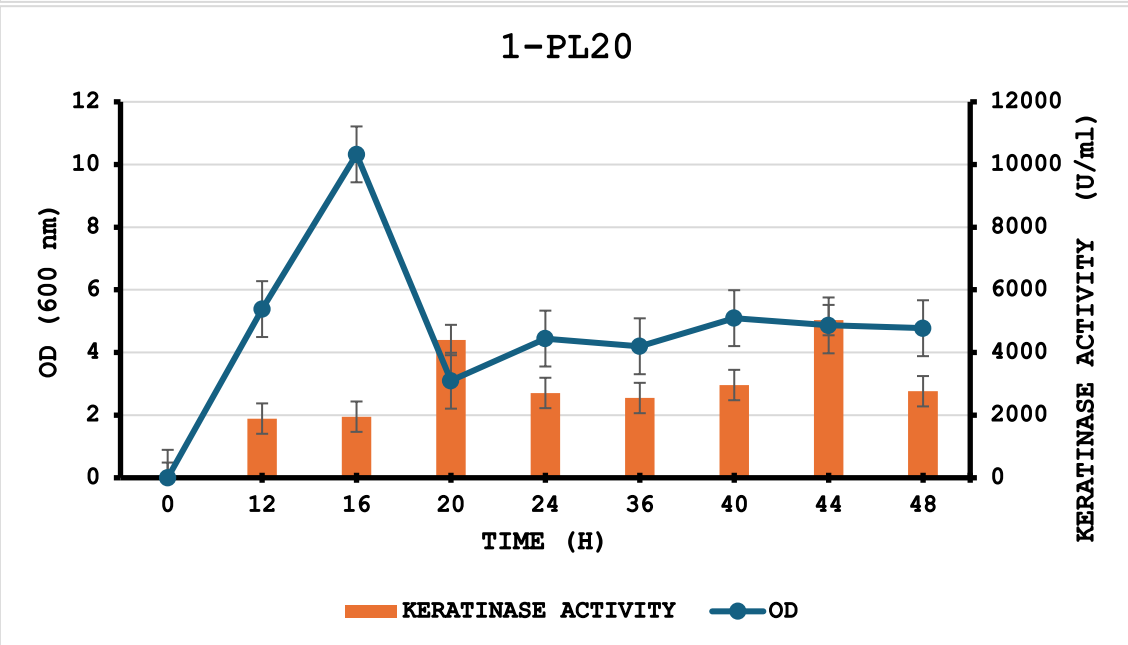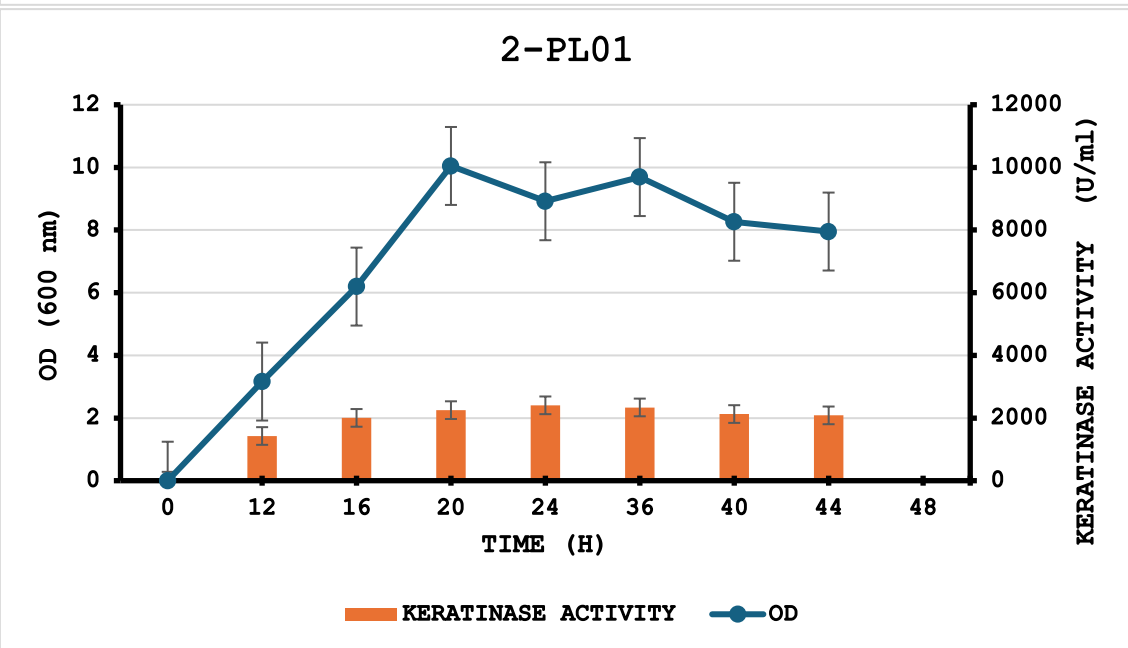

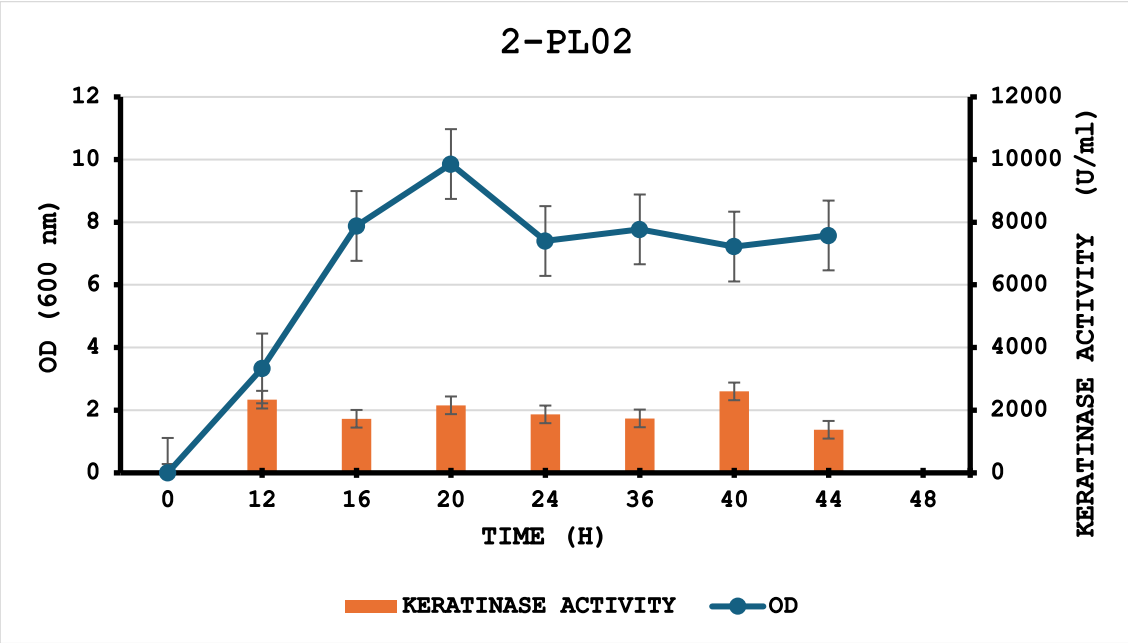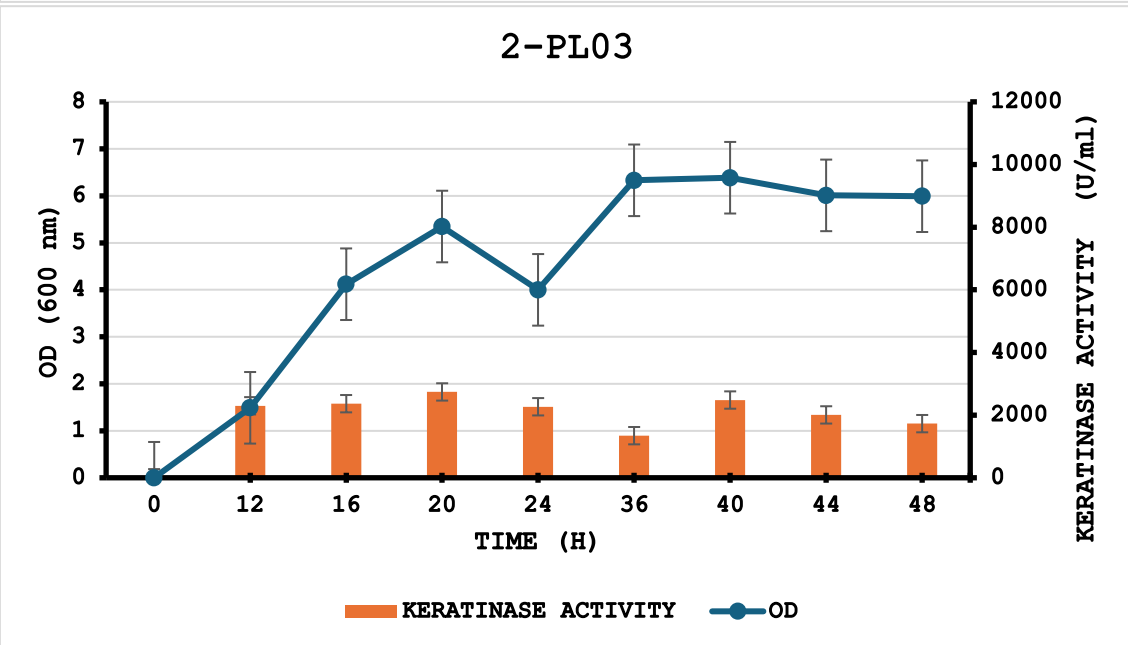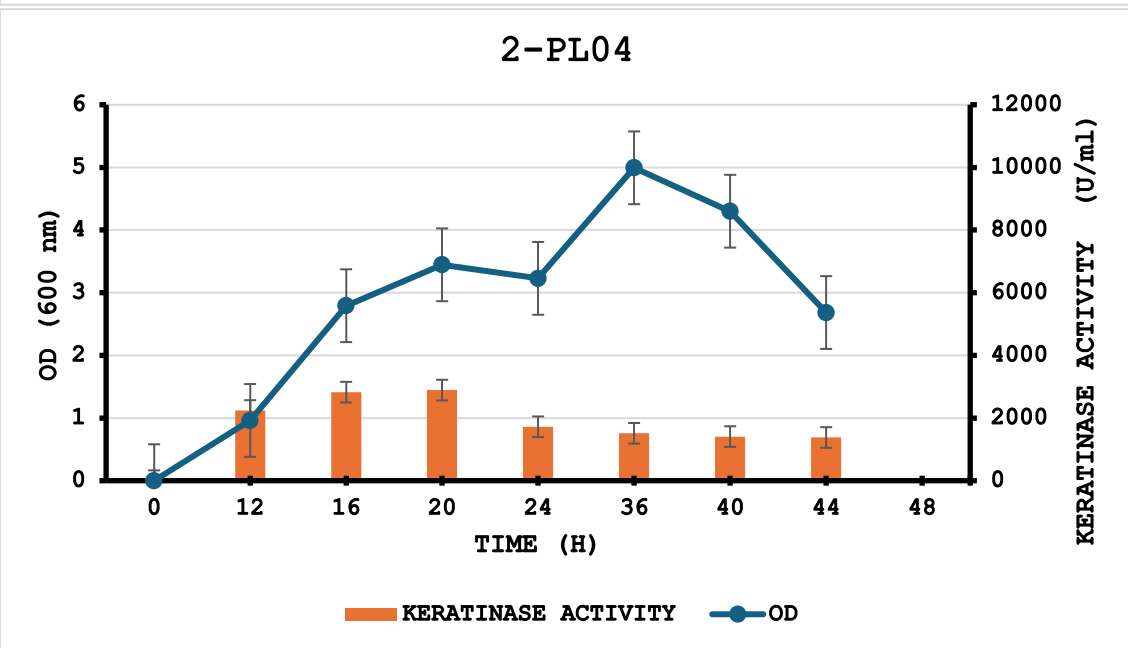

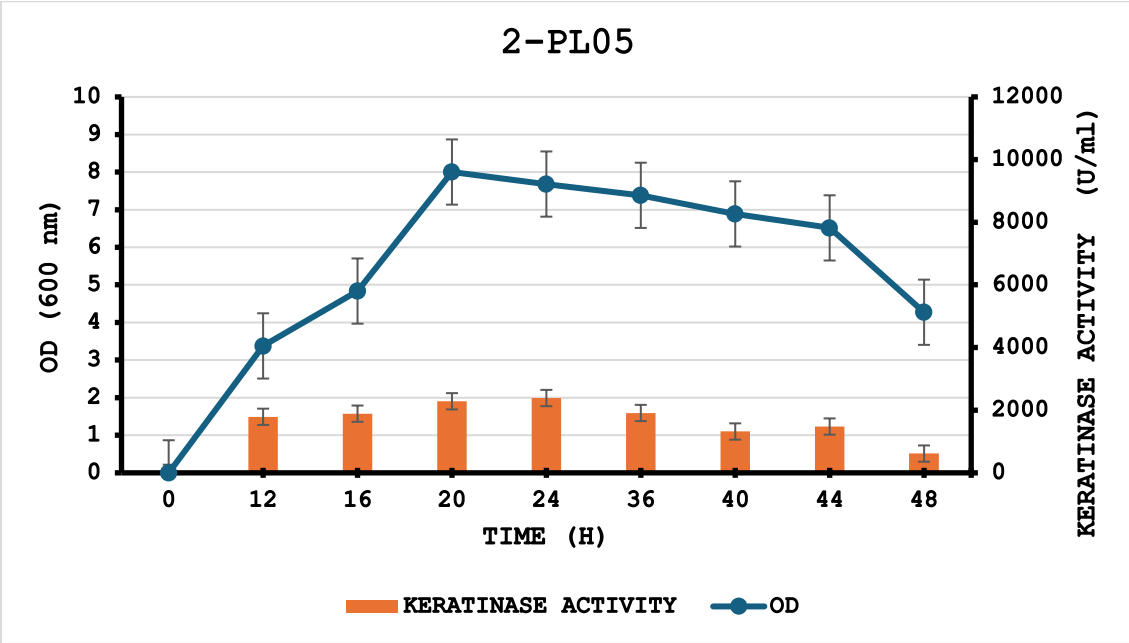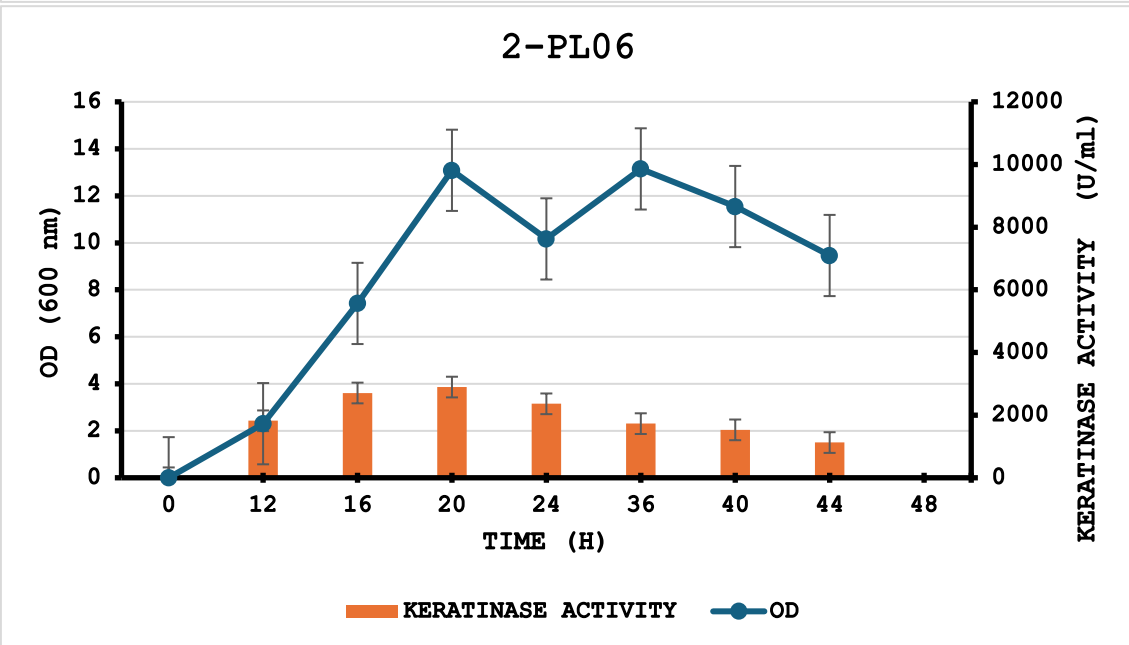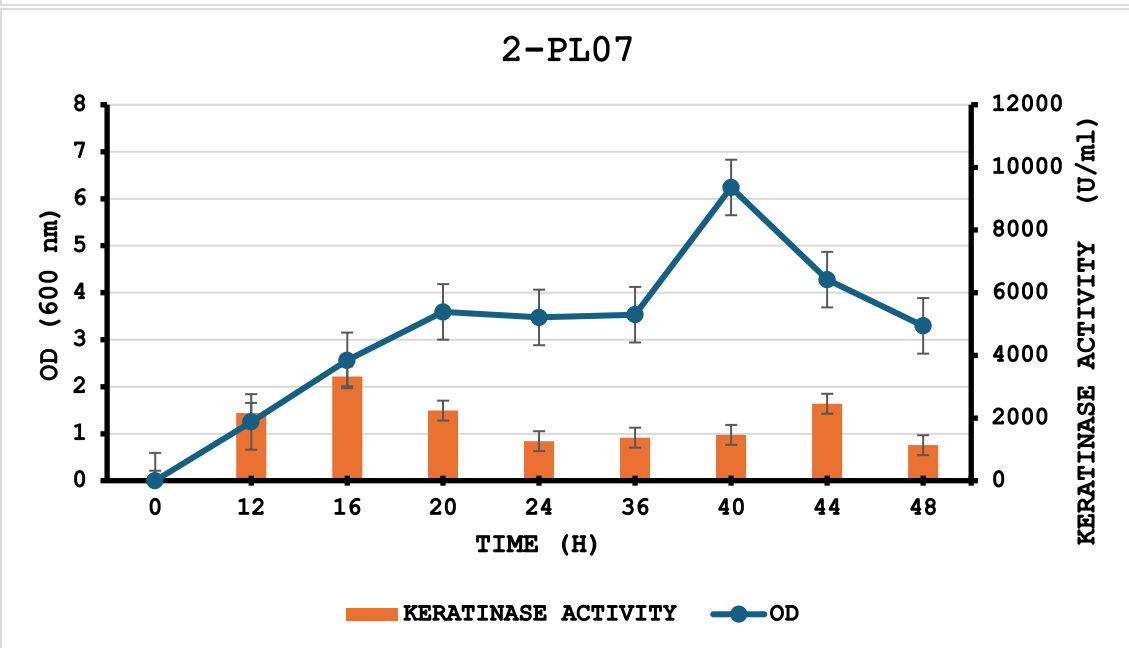

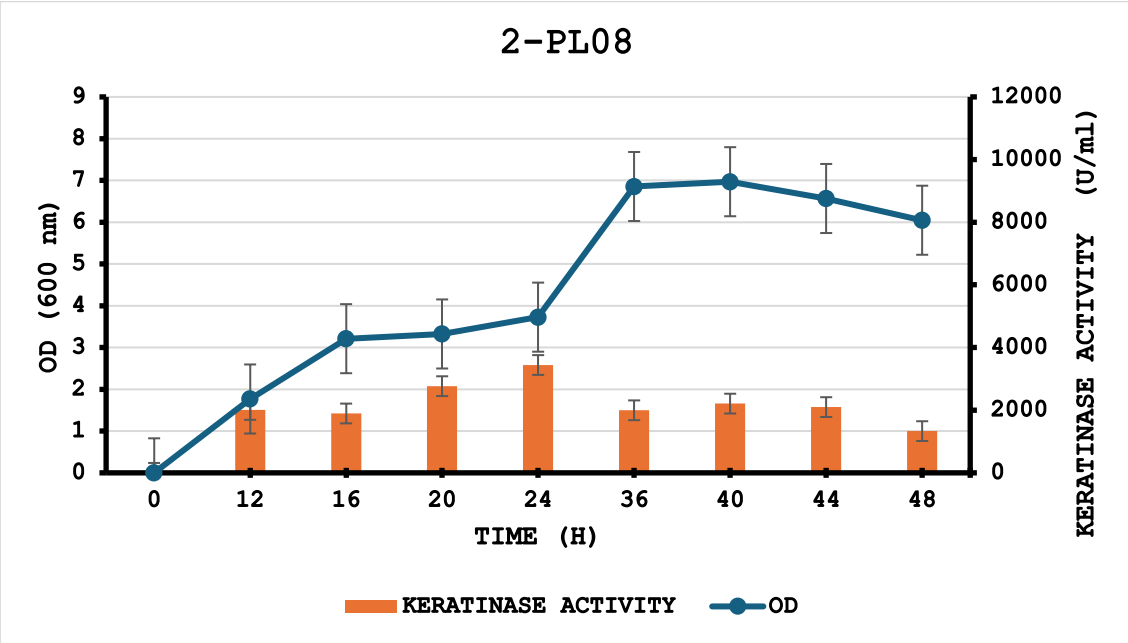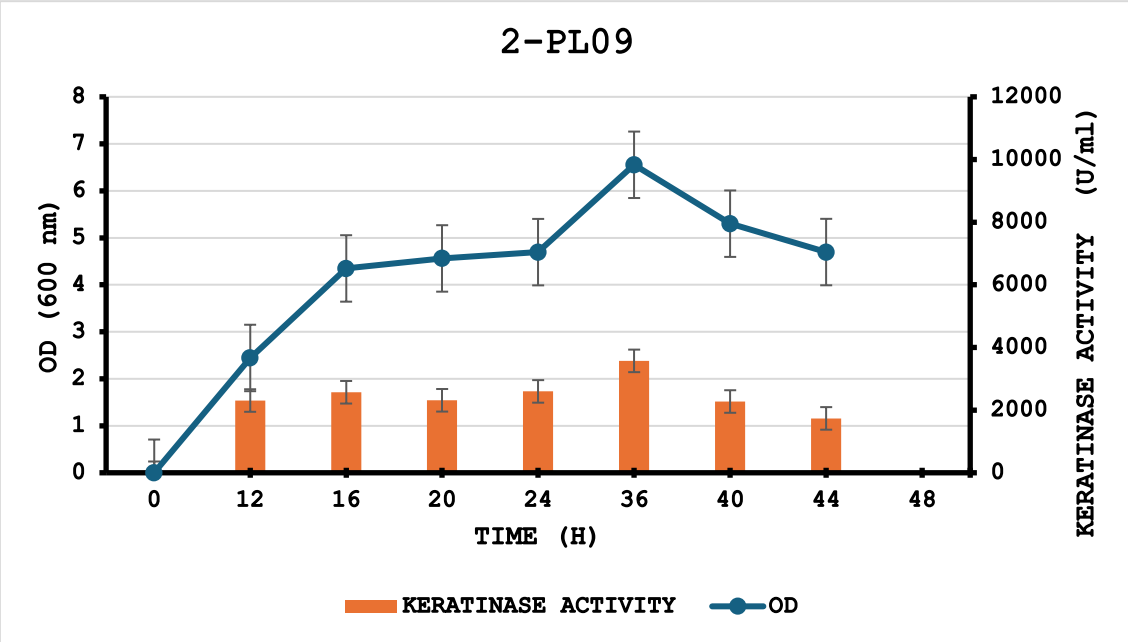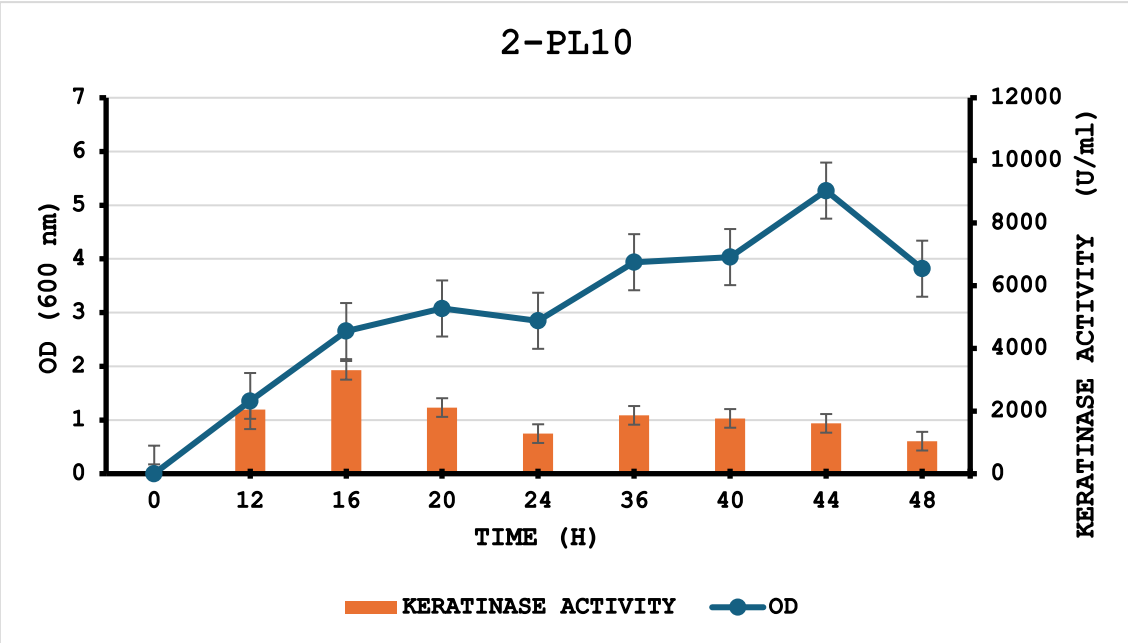

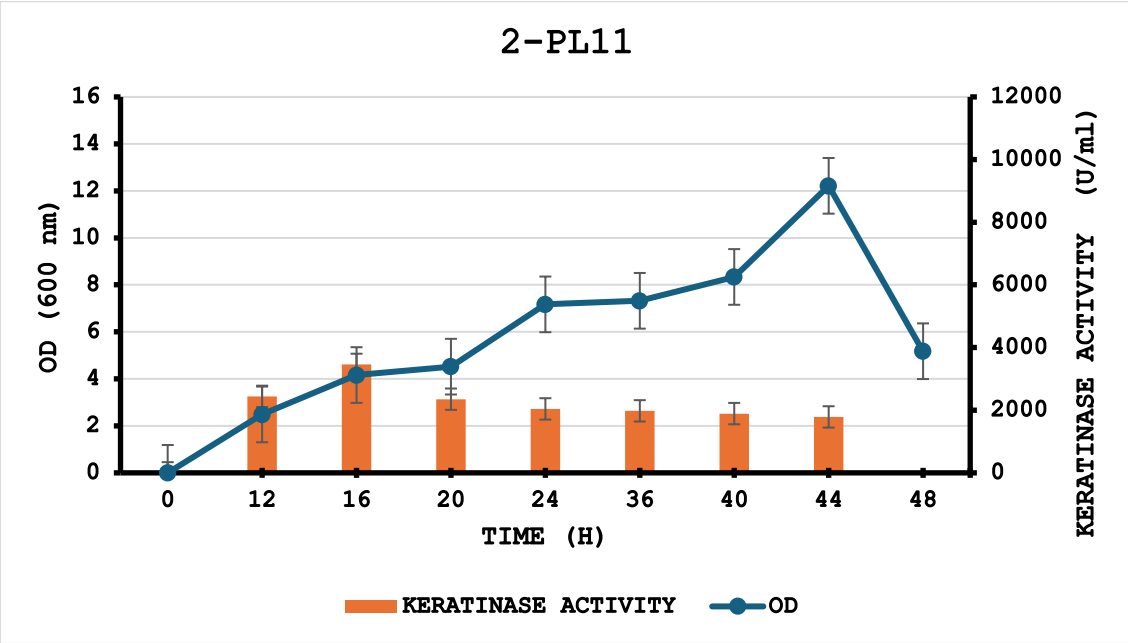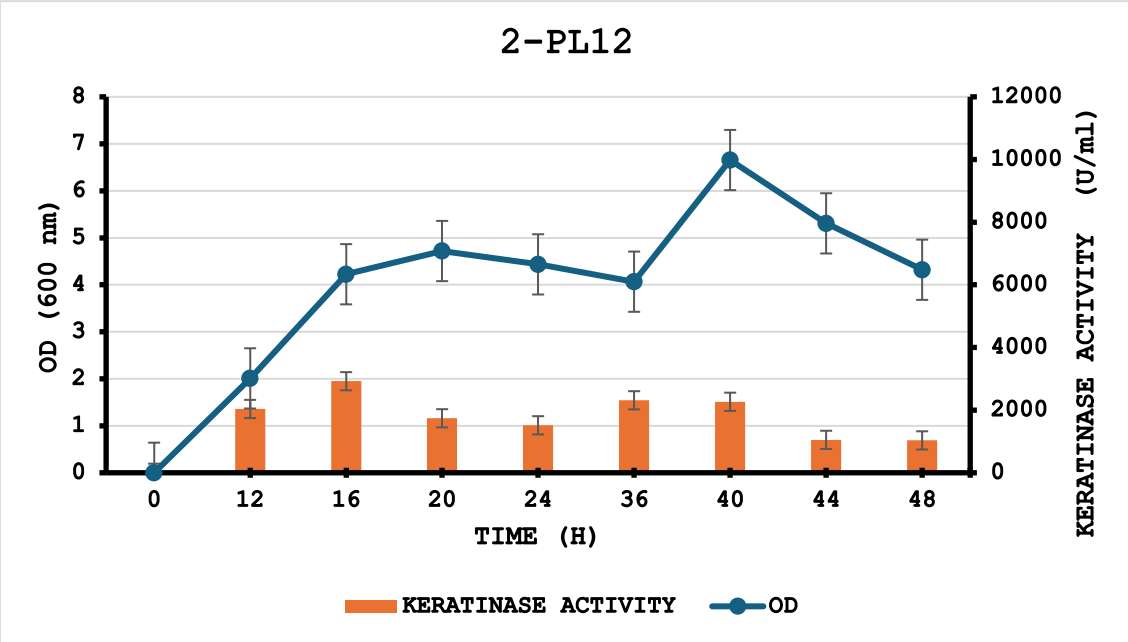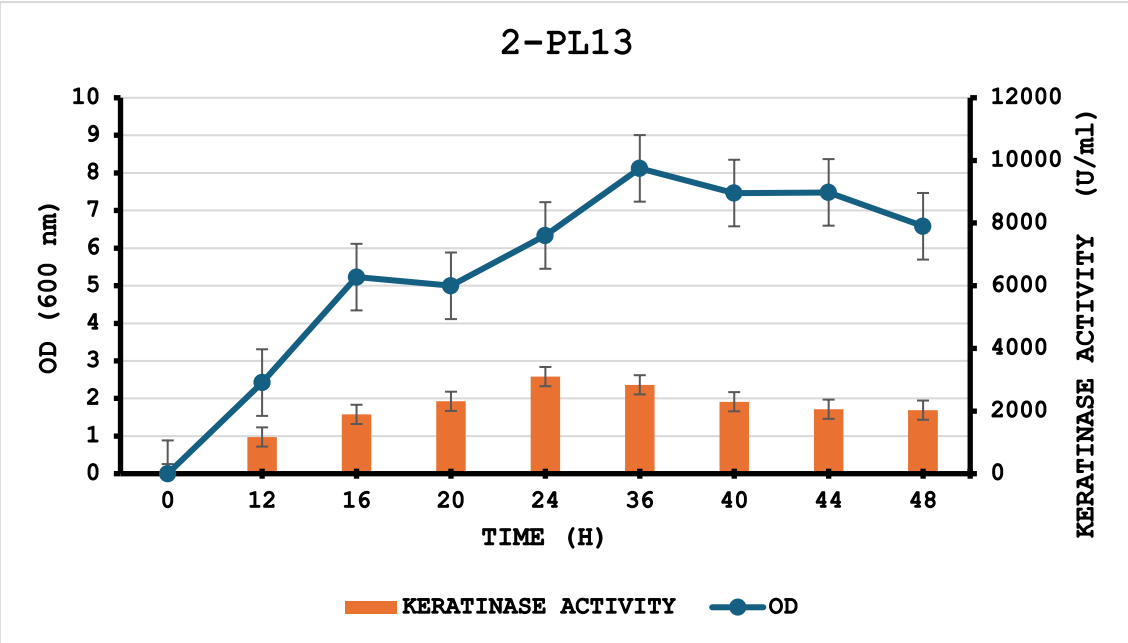

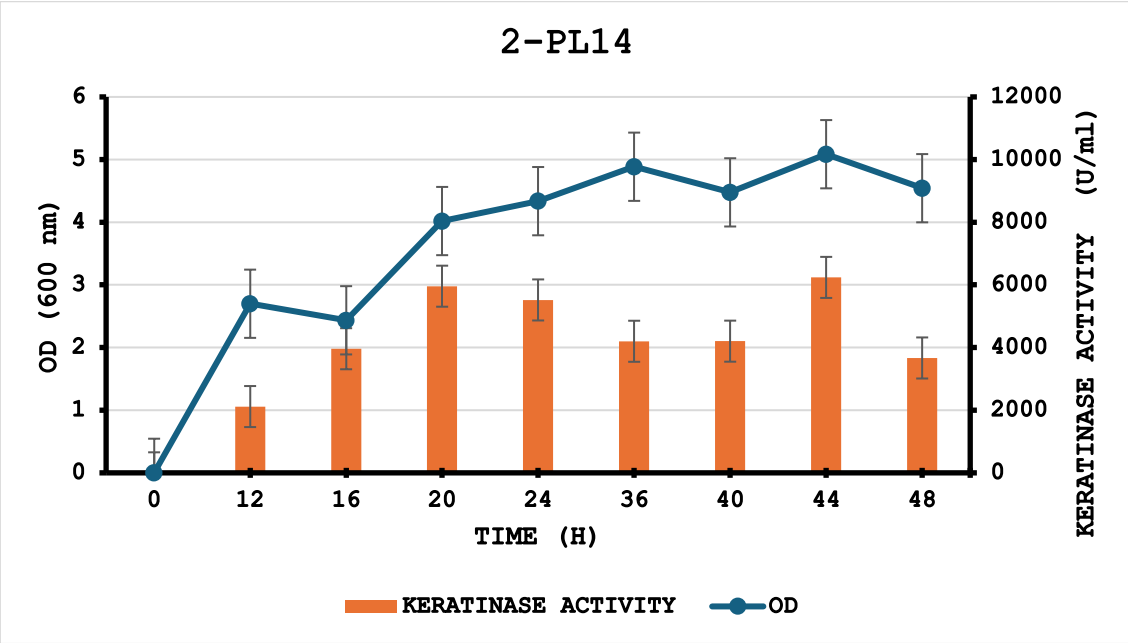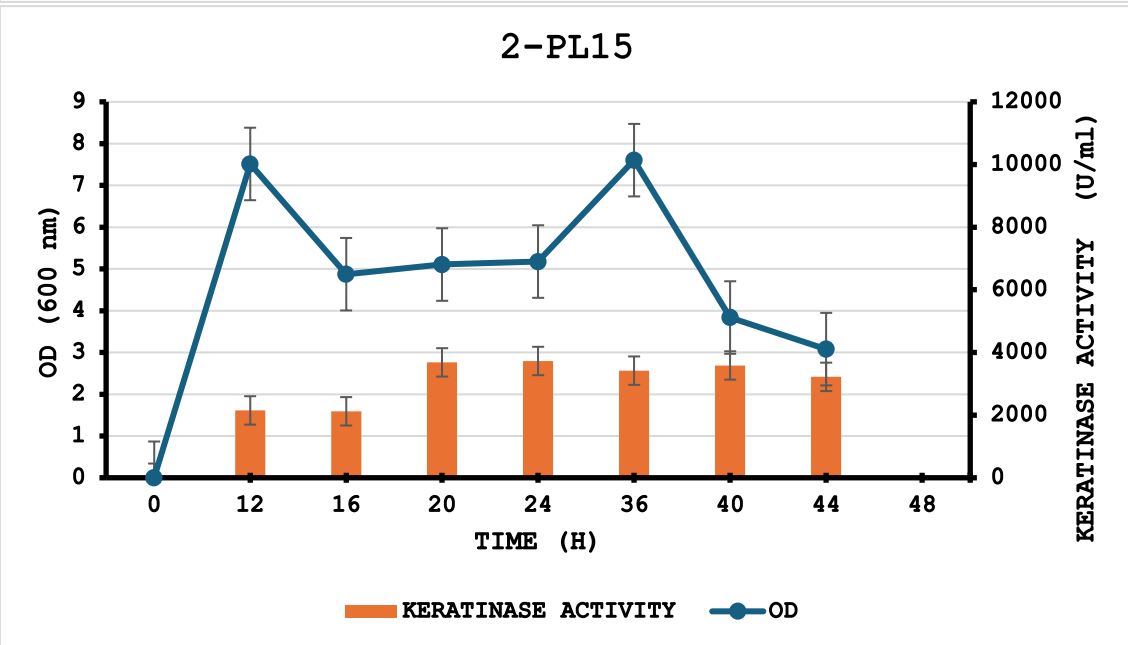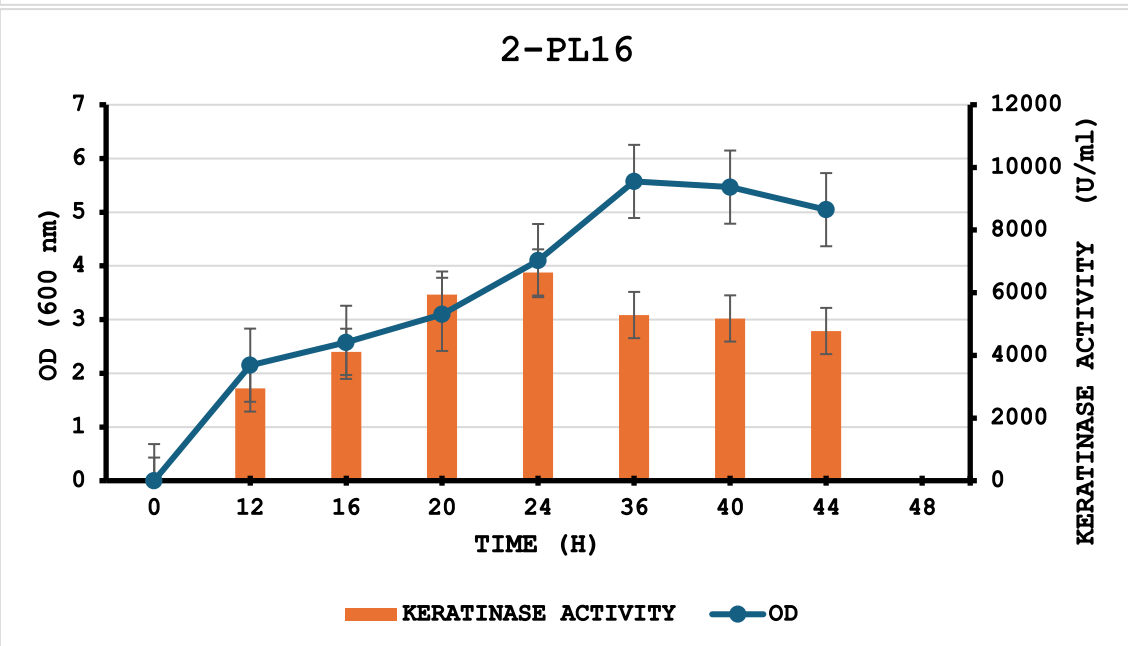

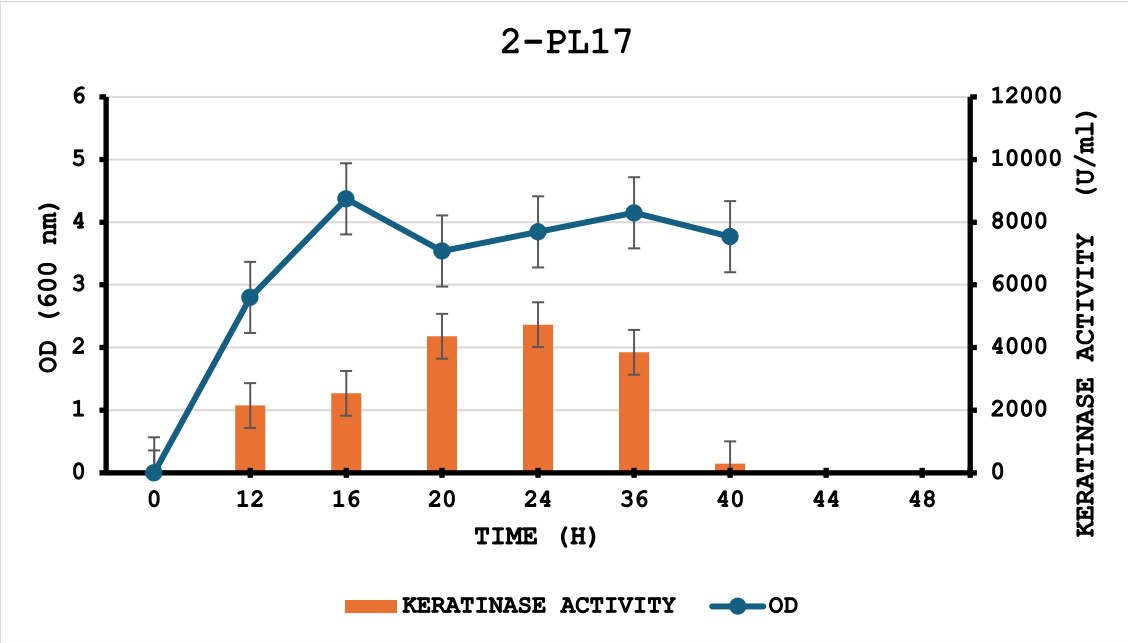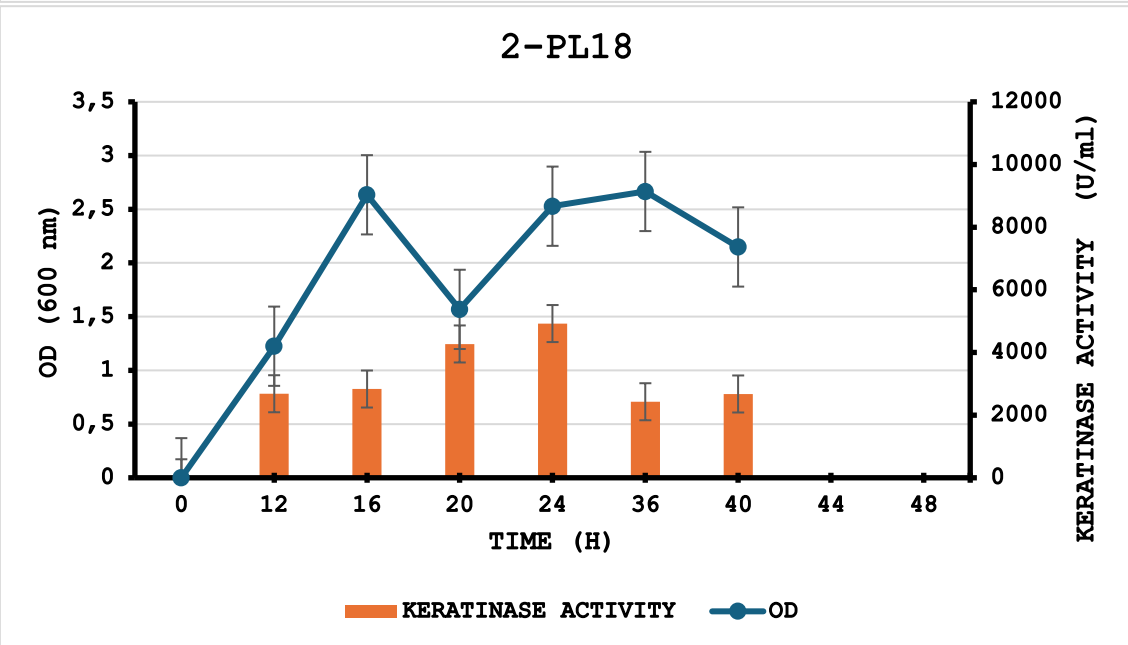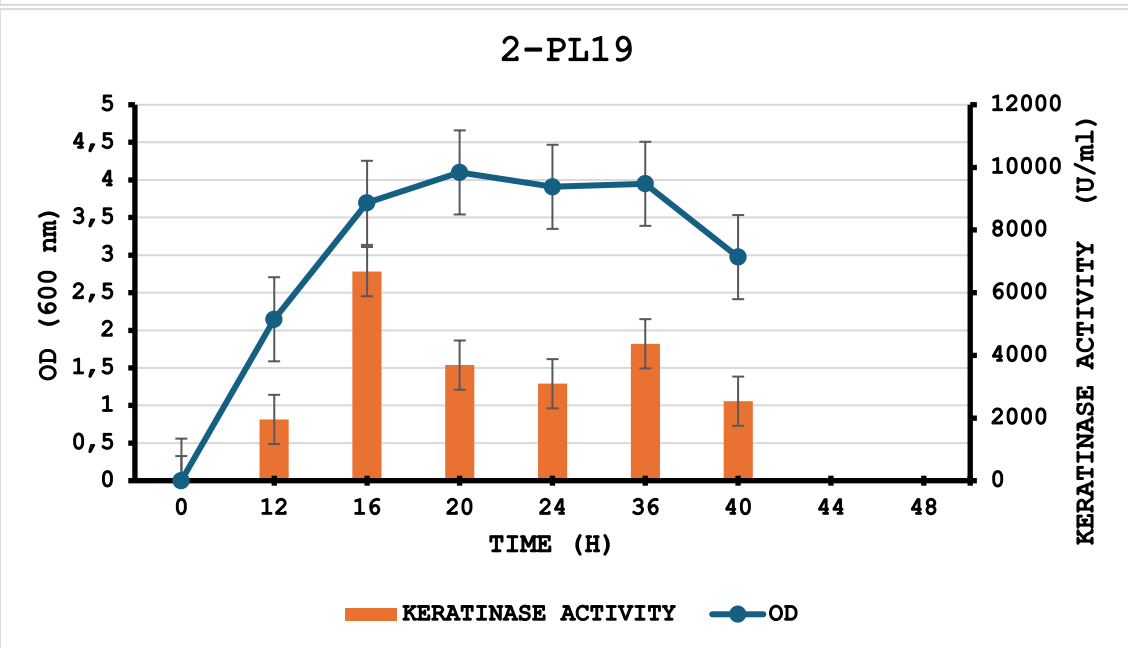

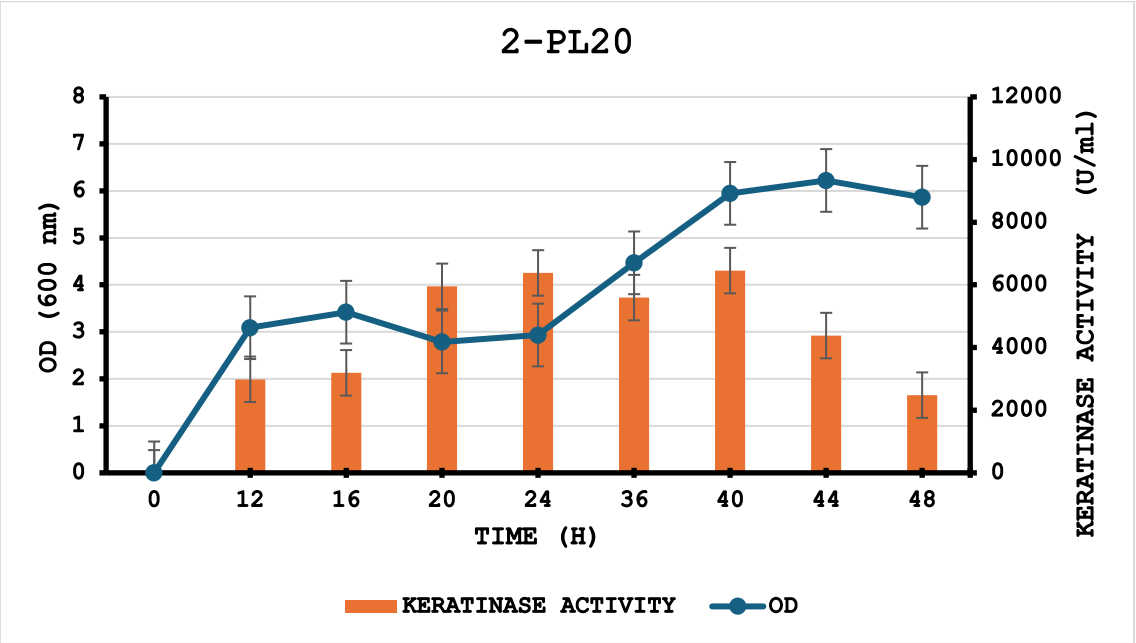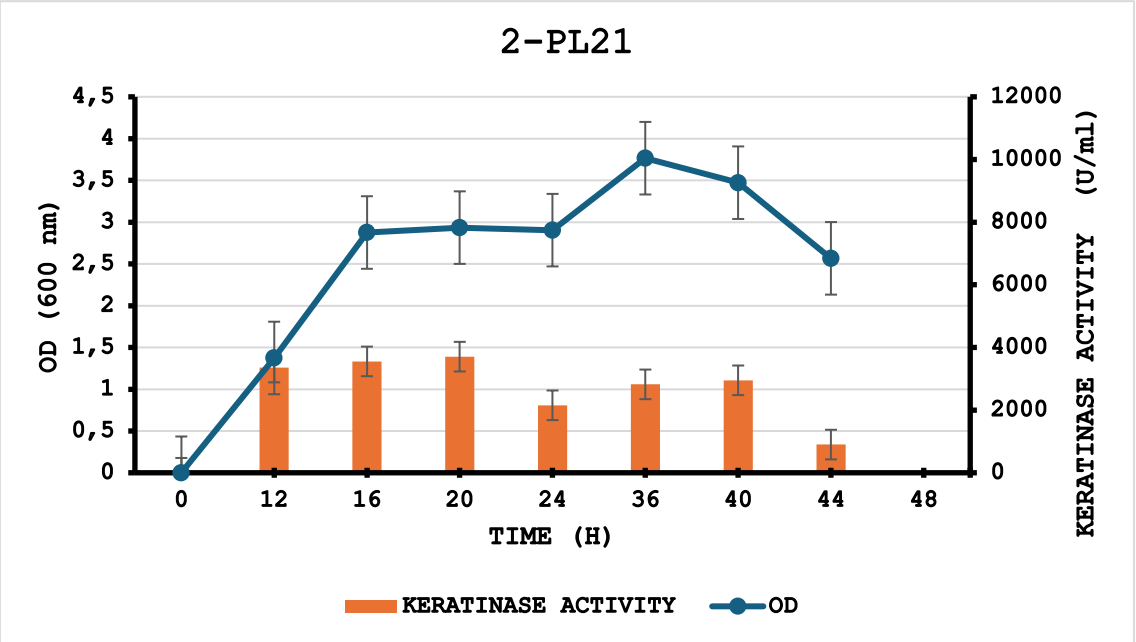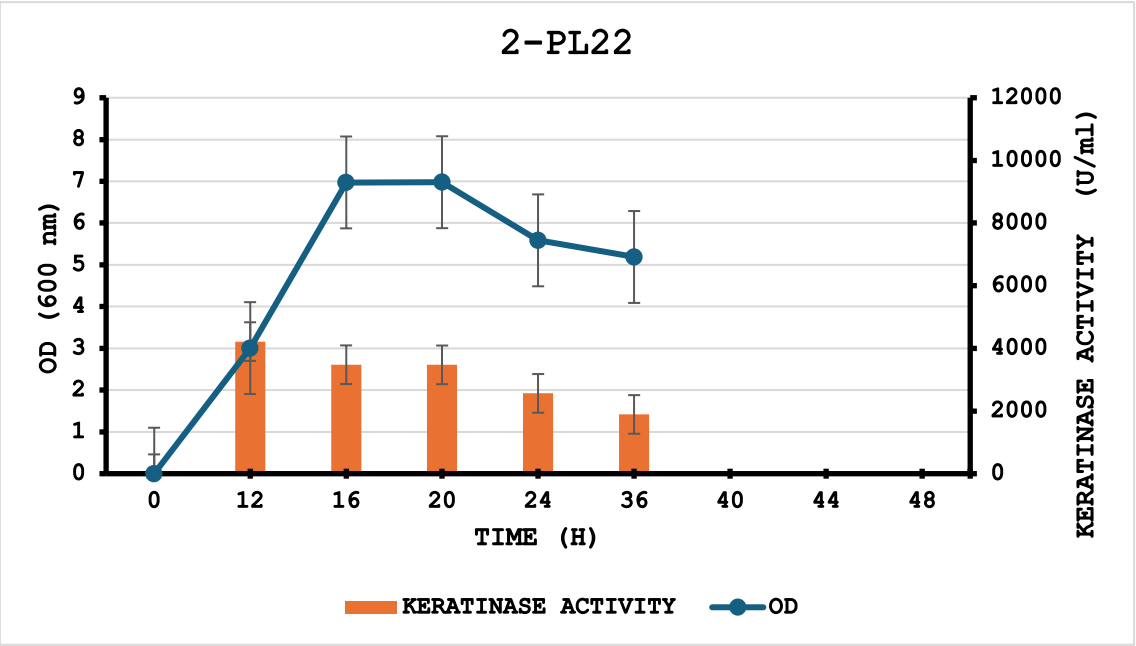

### 2-PL23

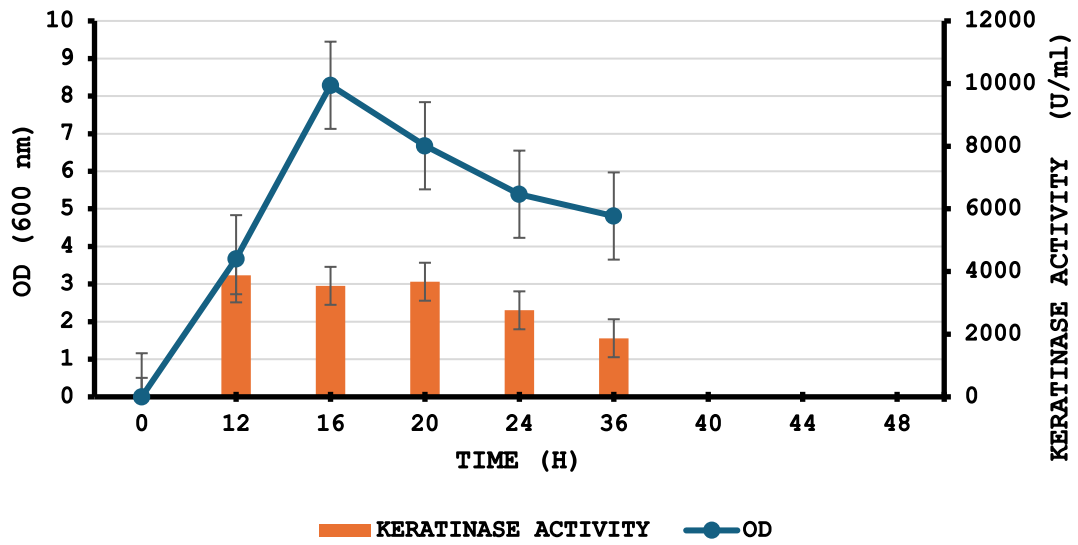

### 2-PL24

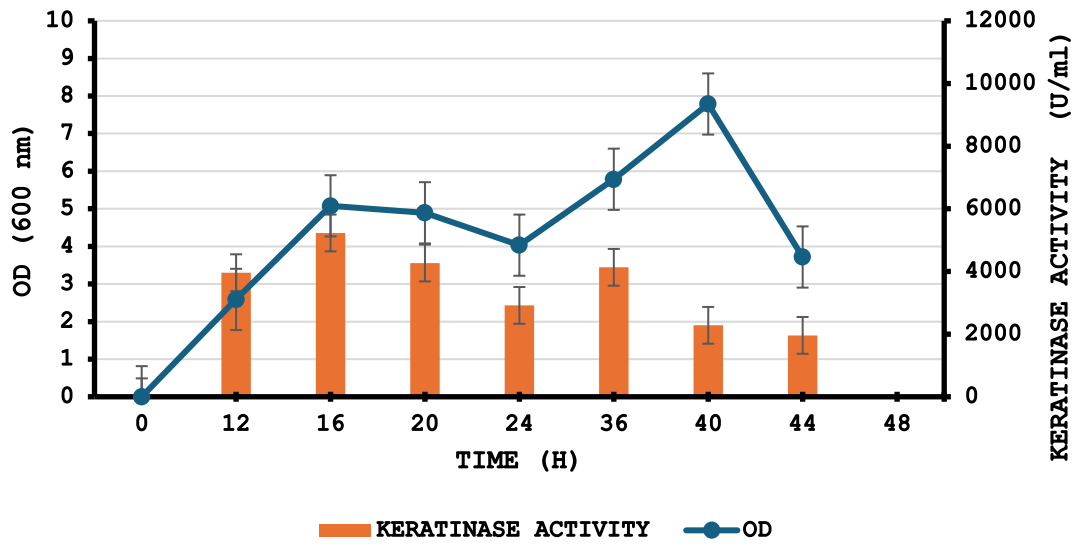

### 2-PL25

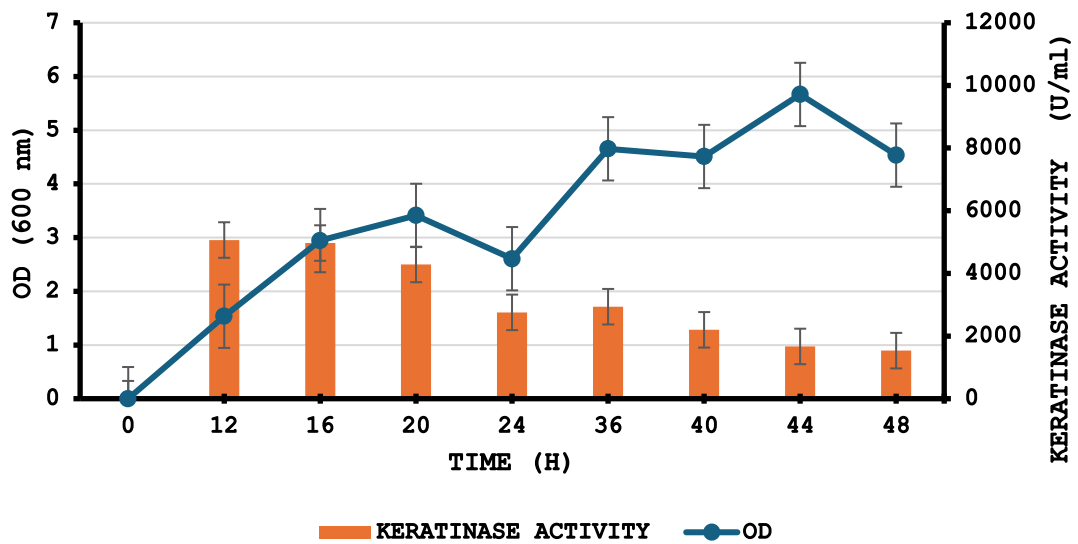

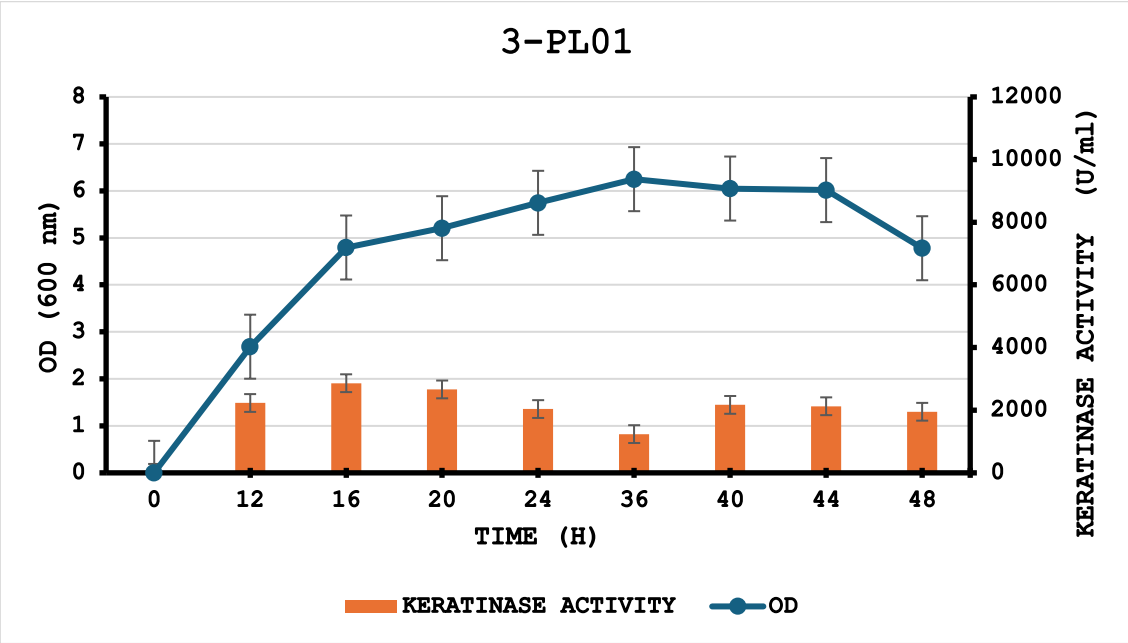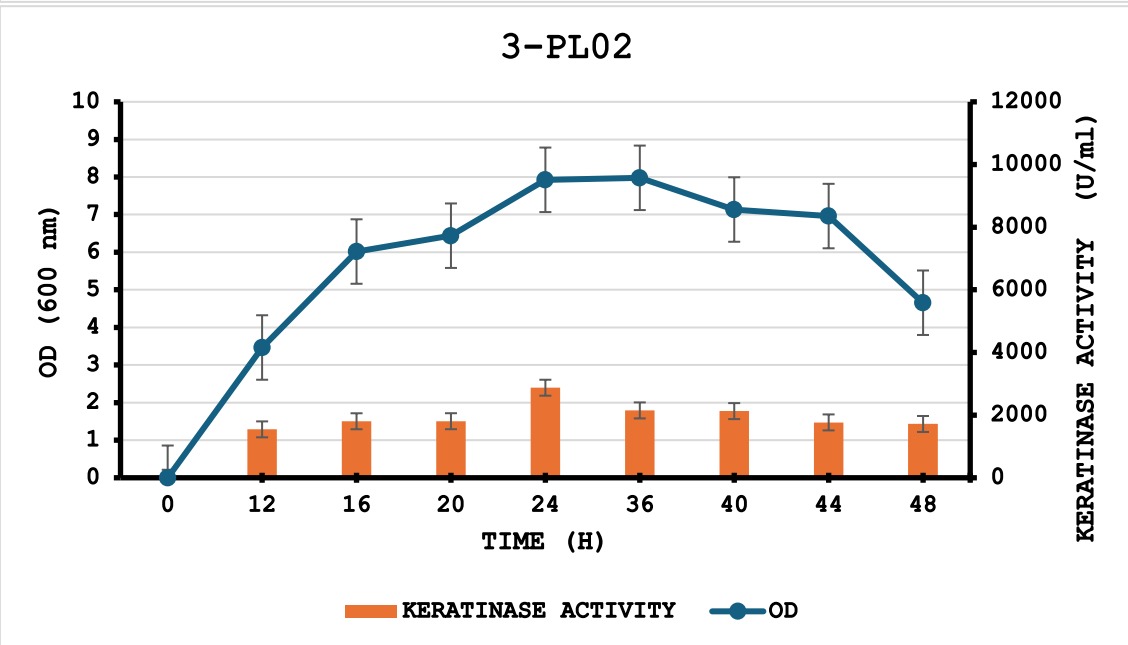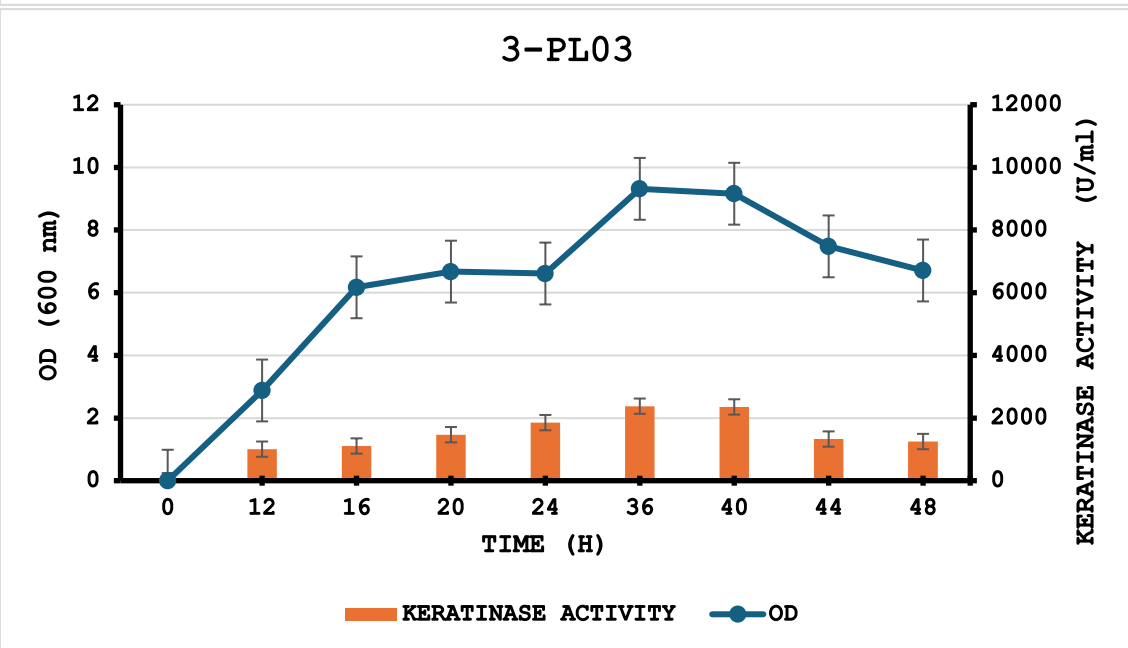

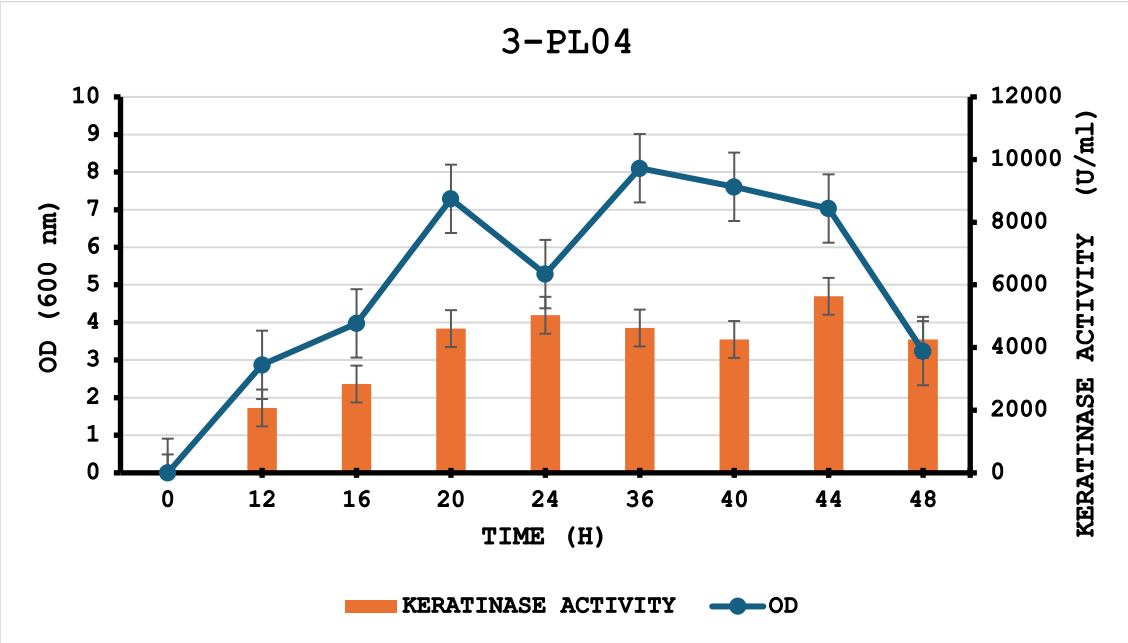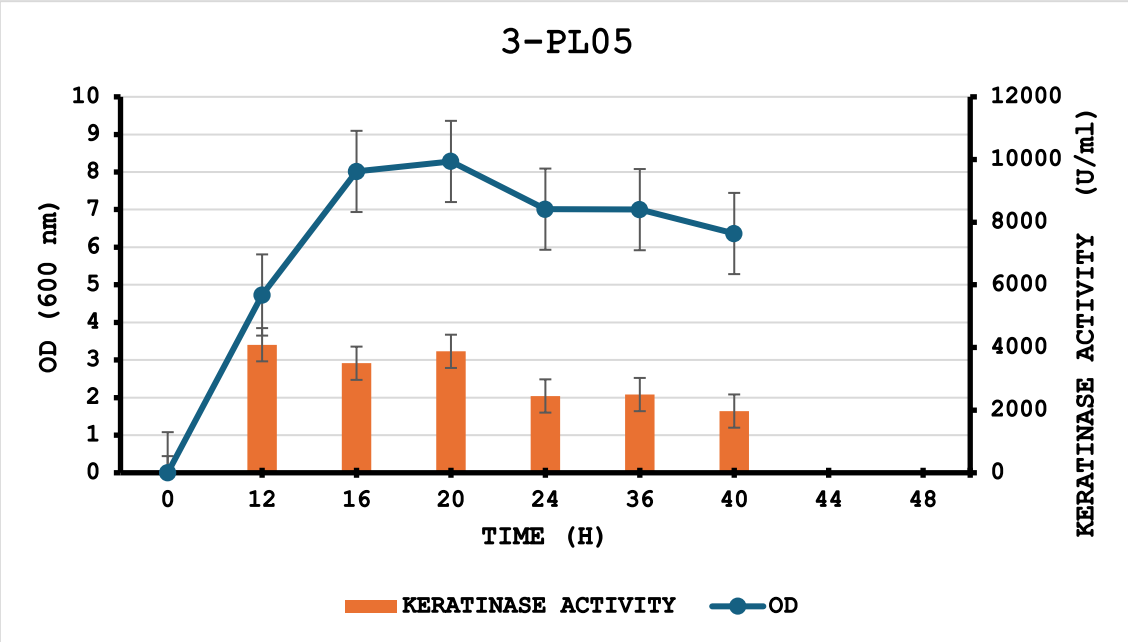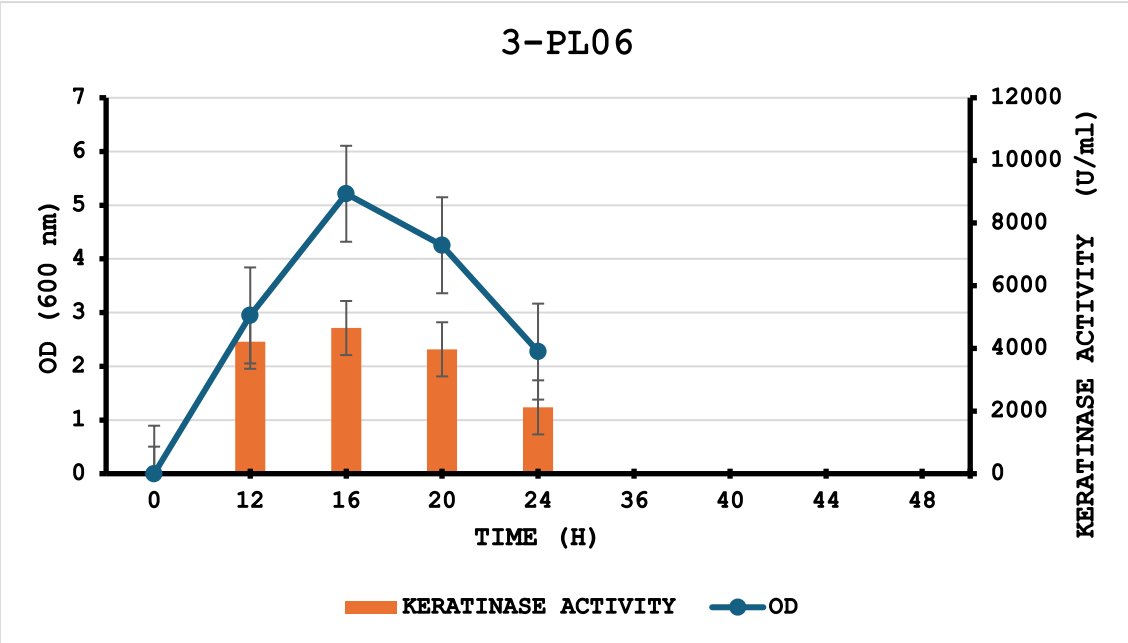

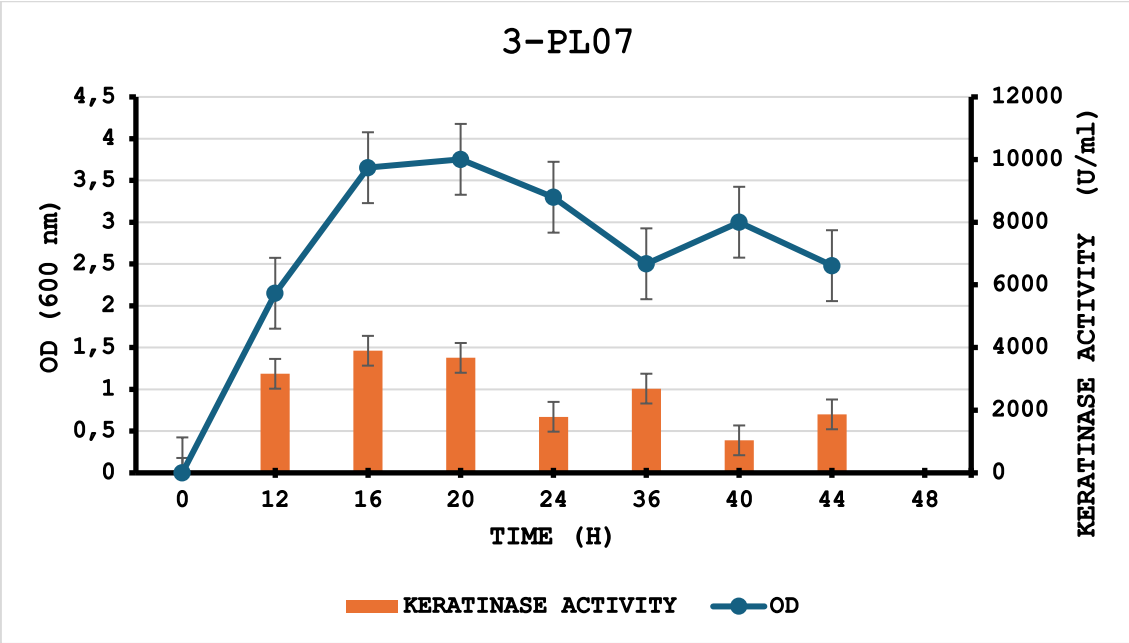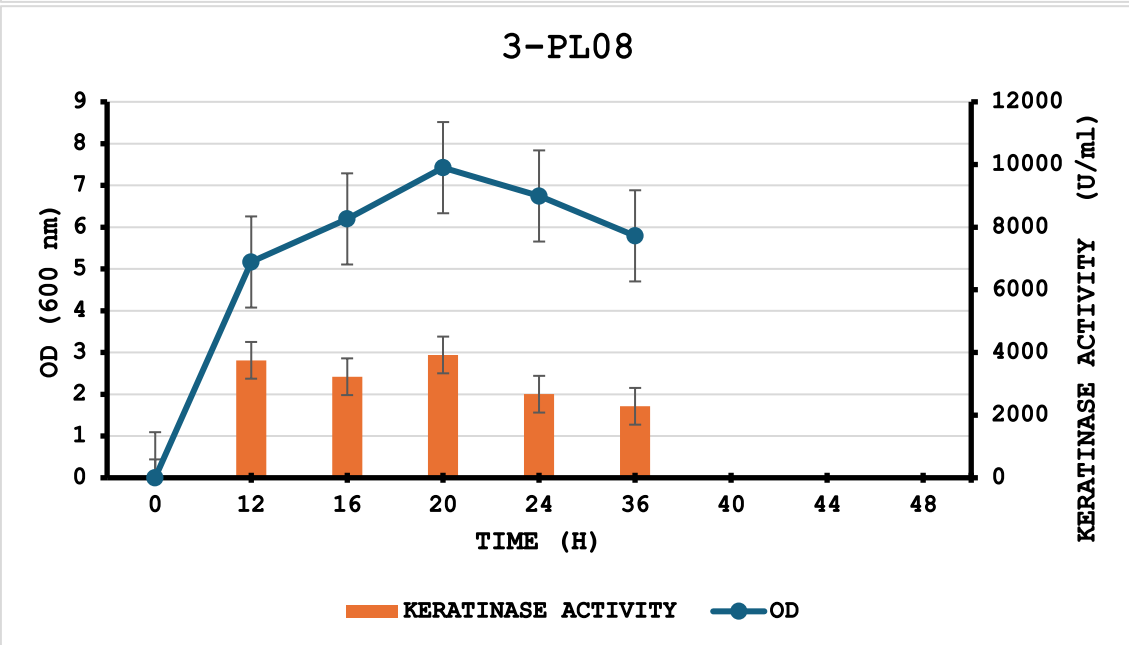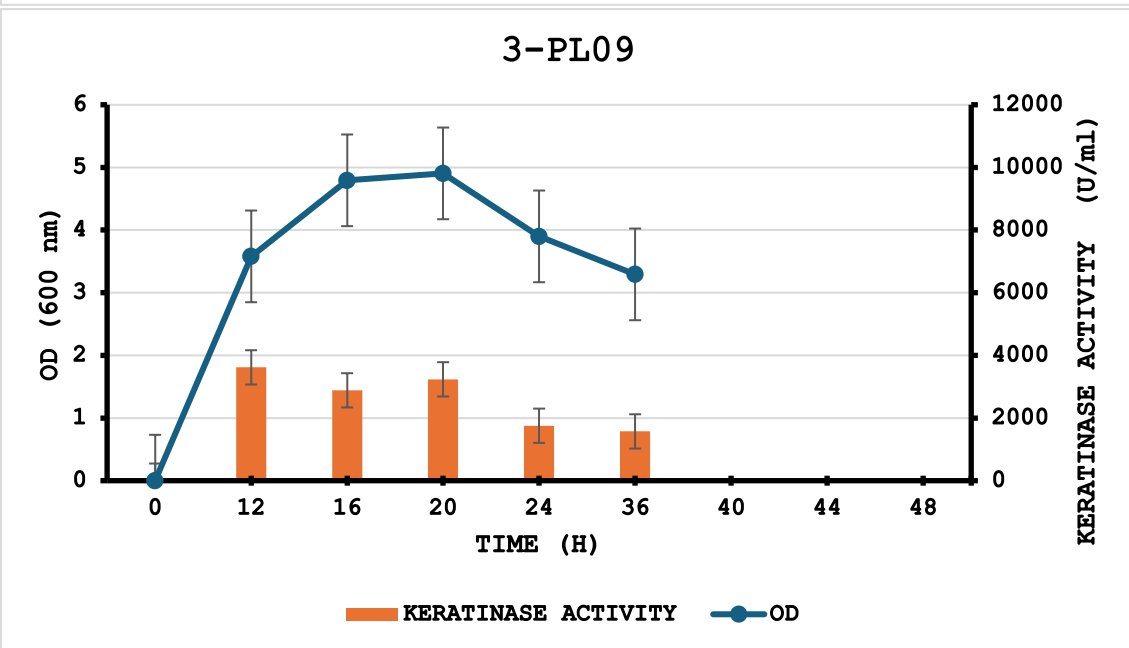

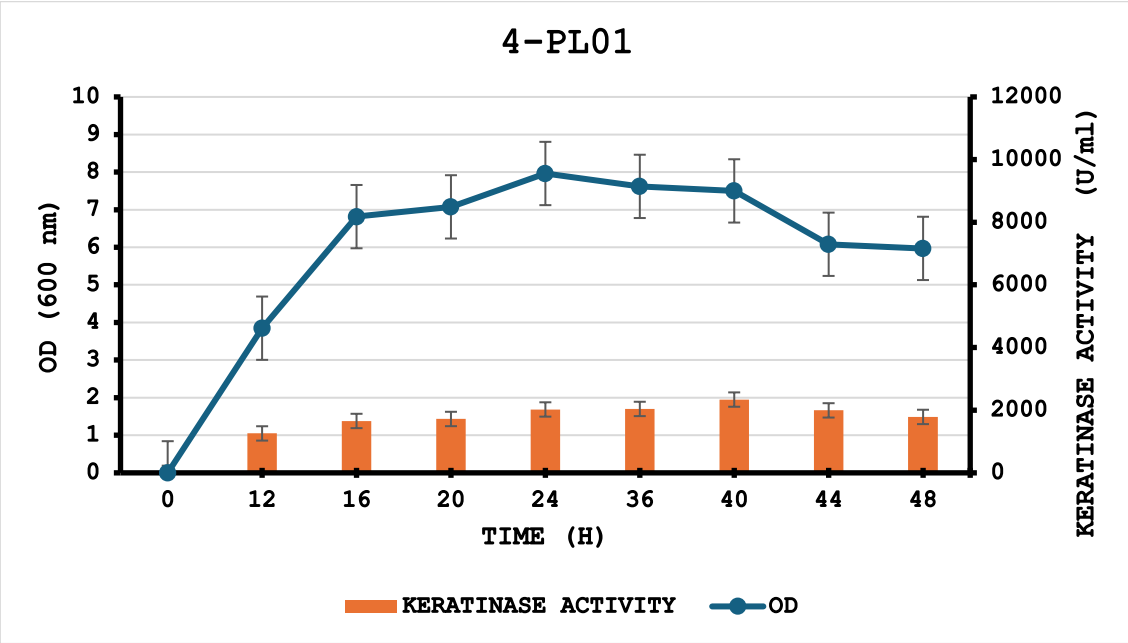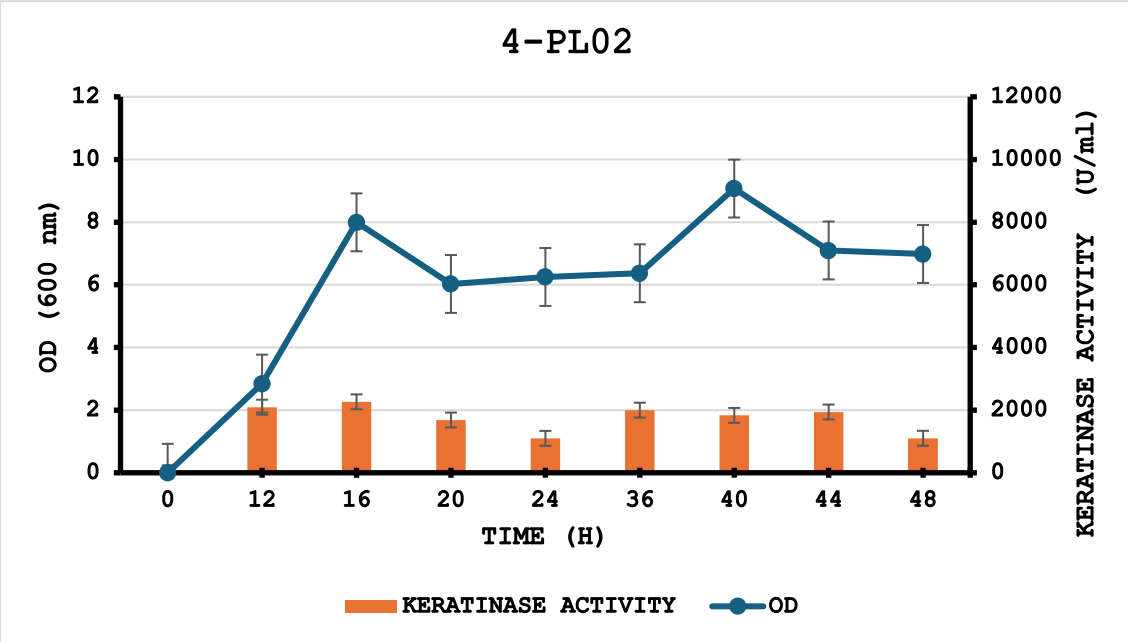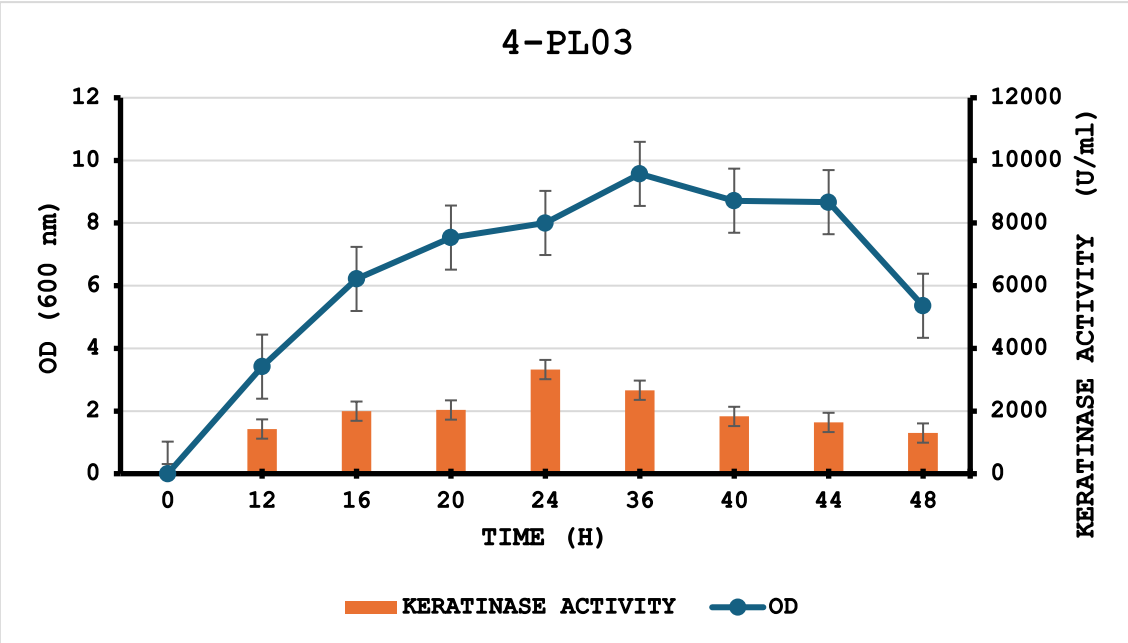

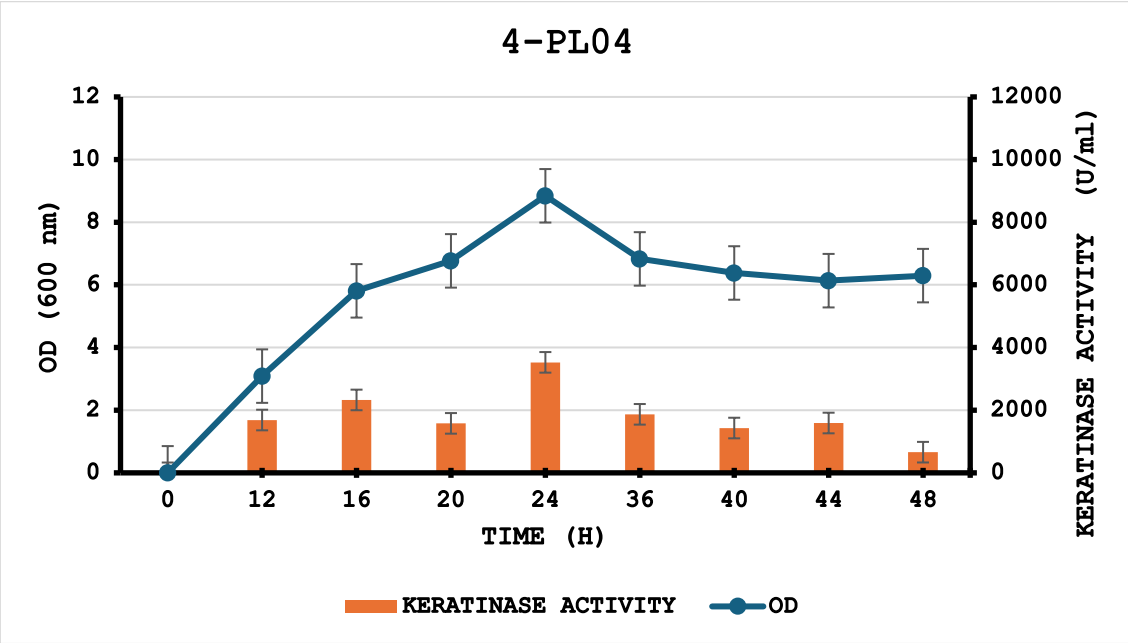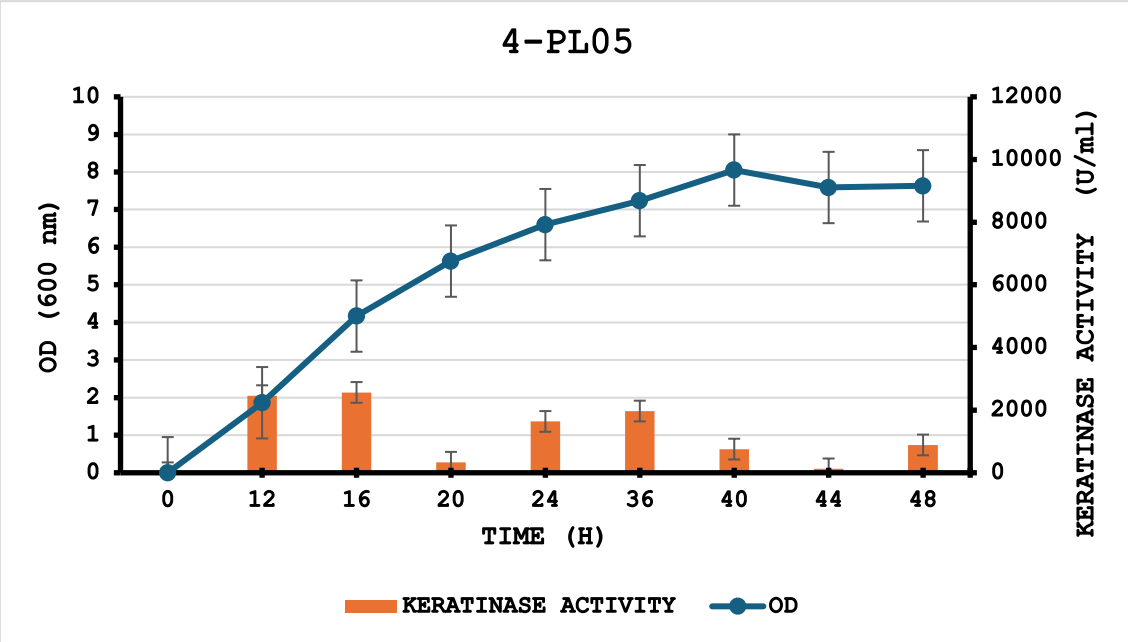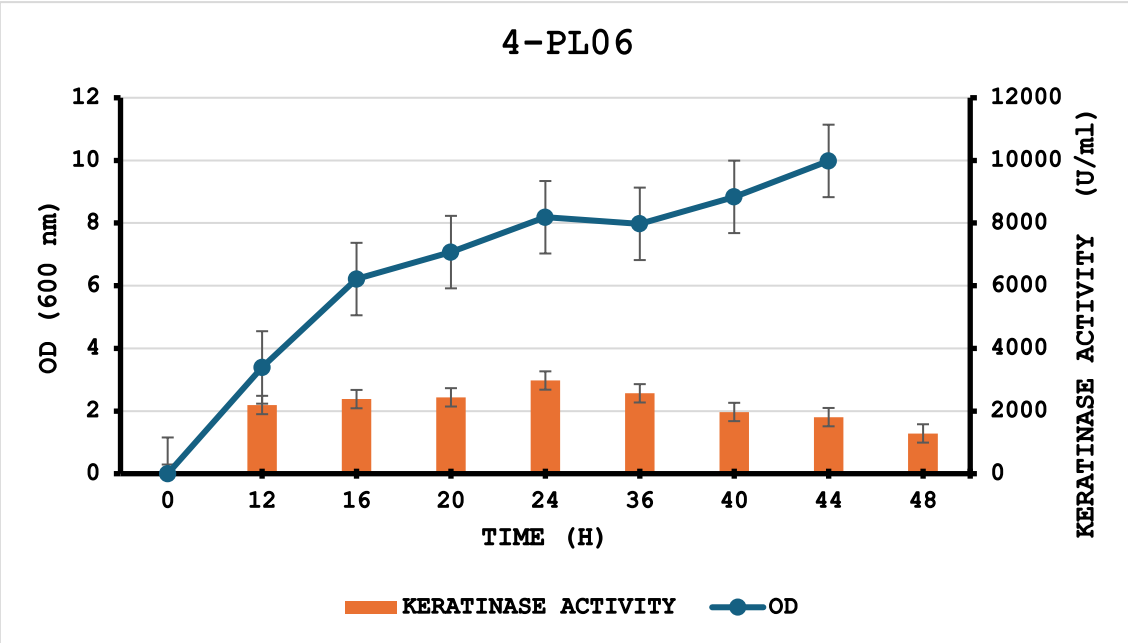

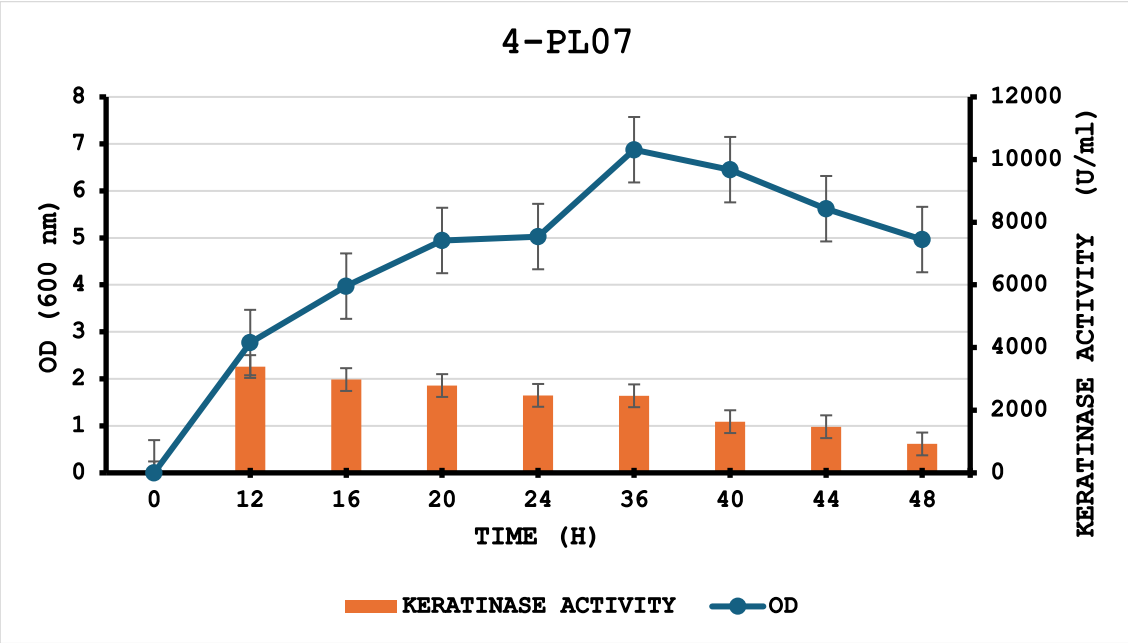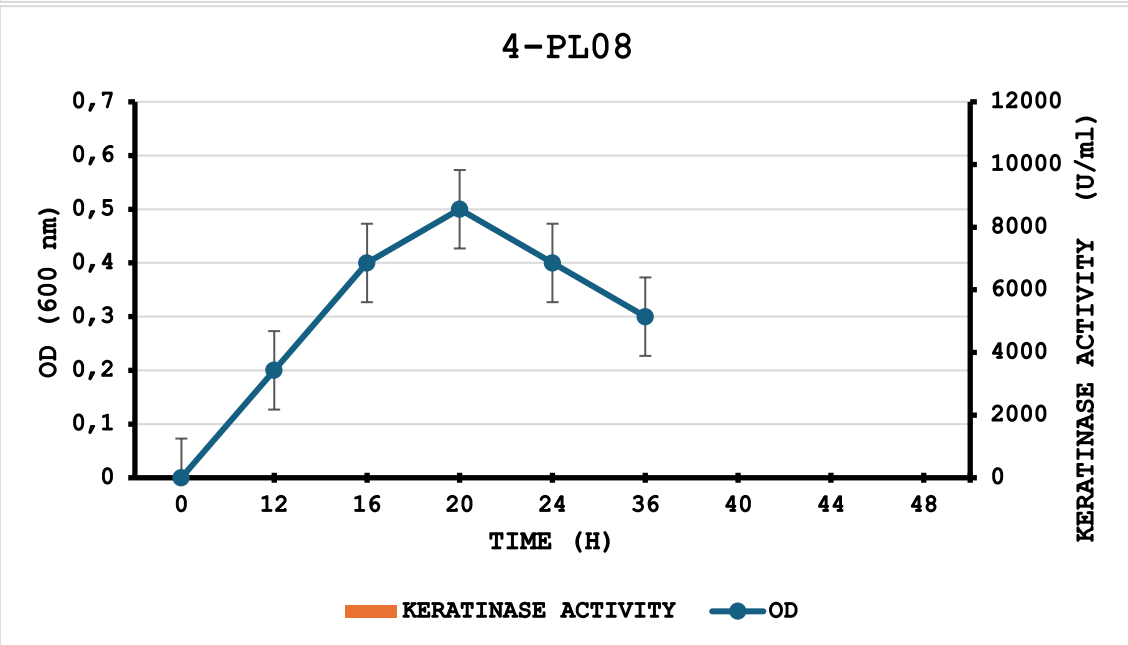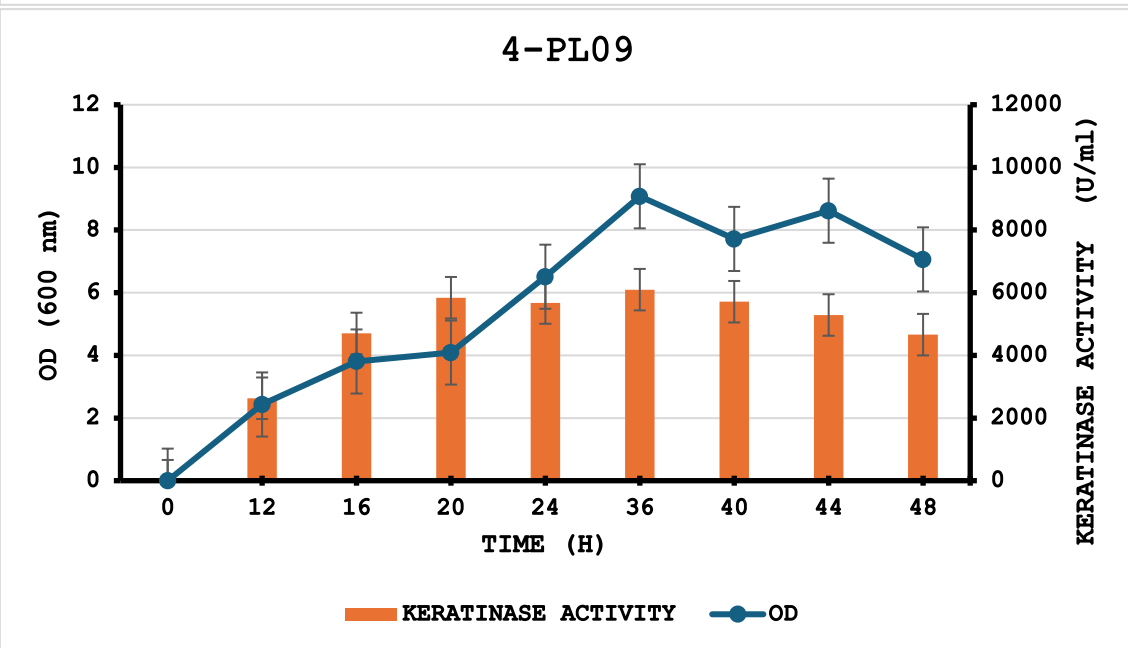

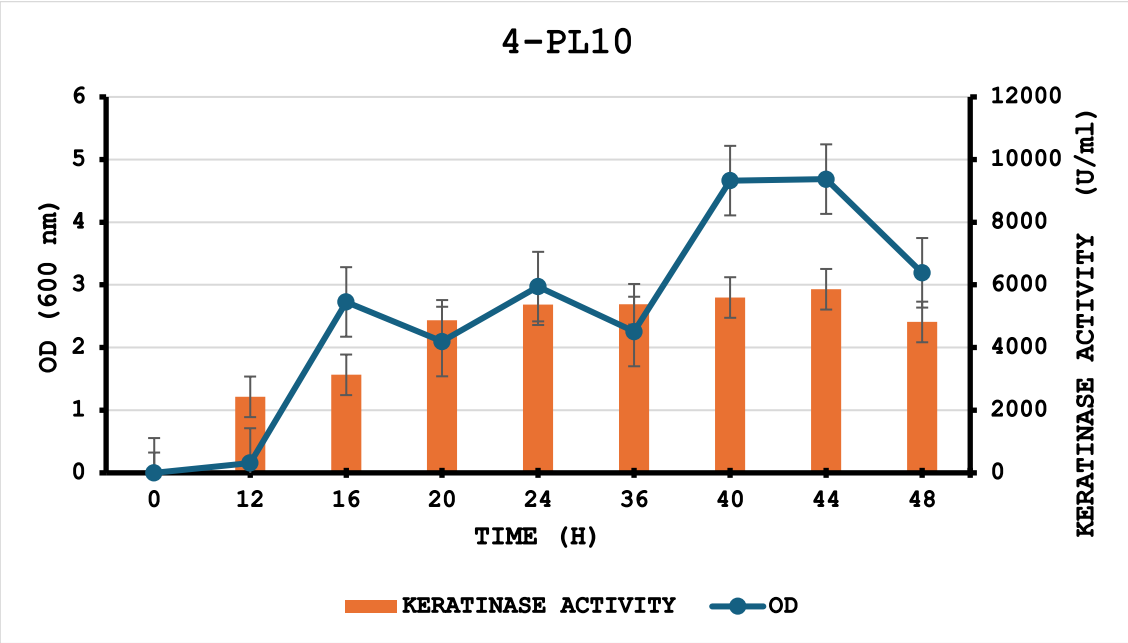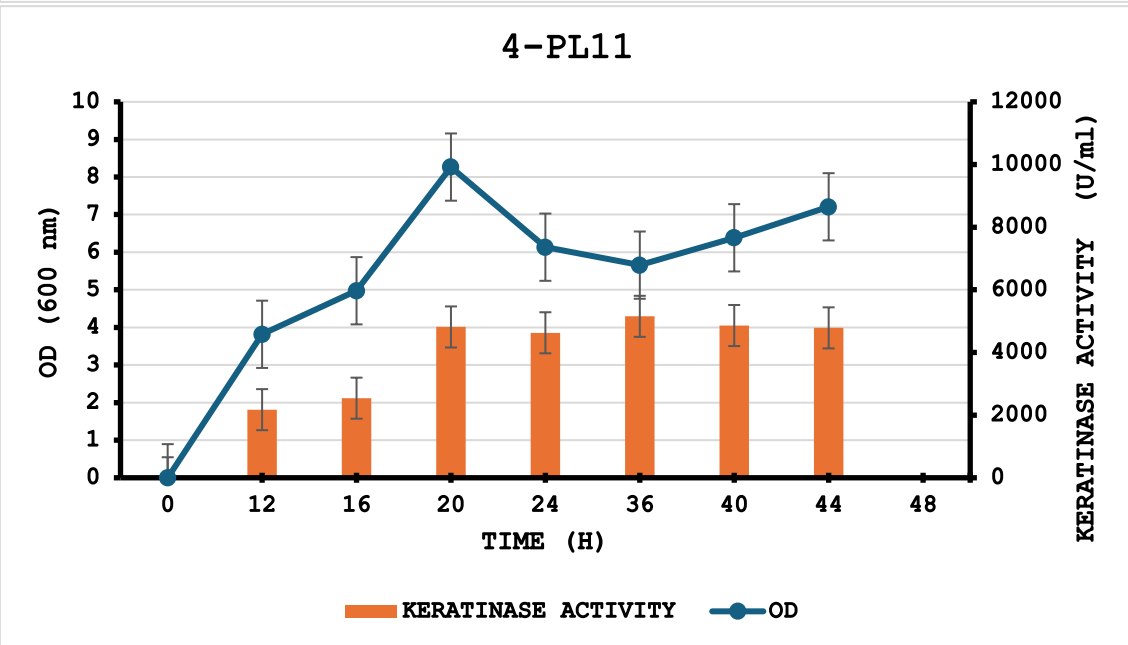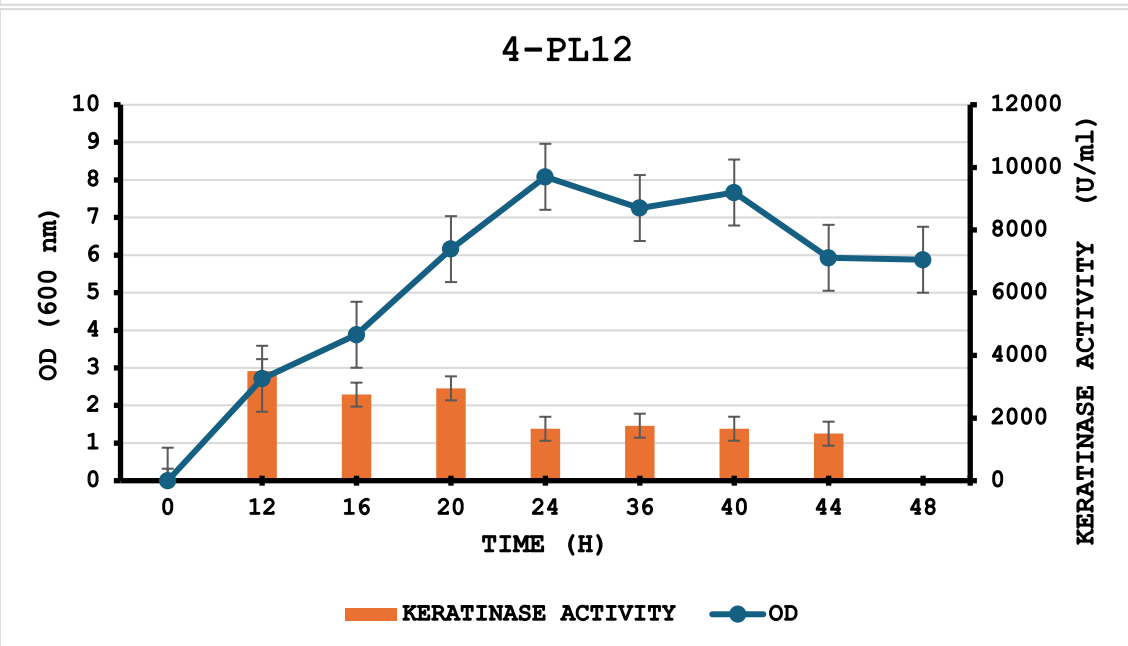

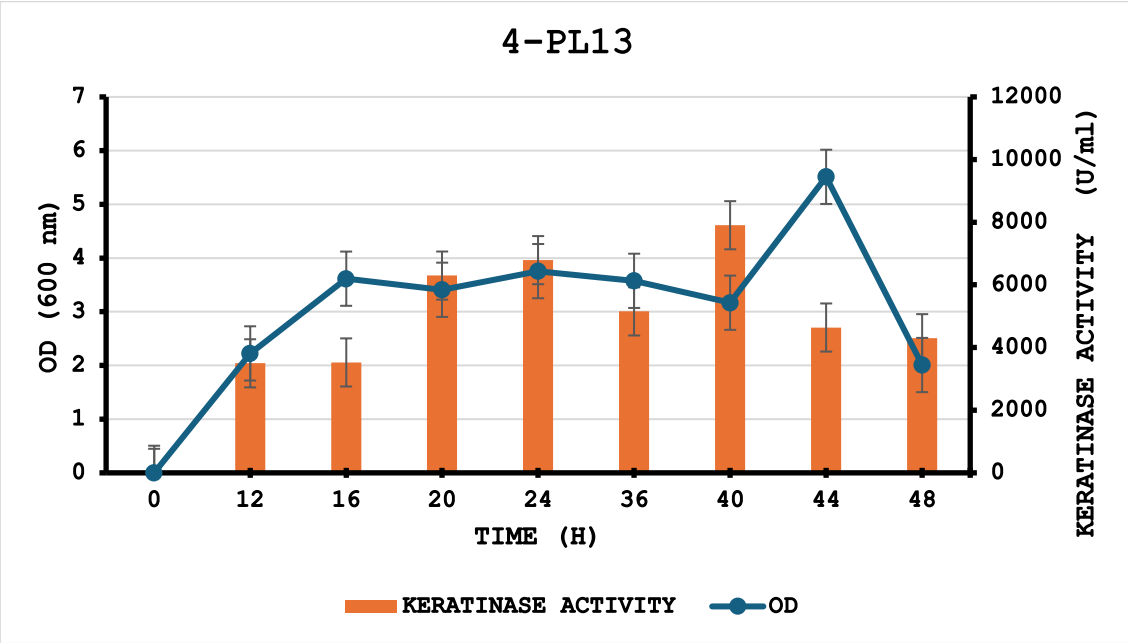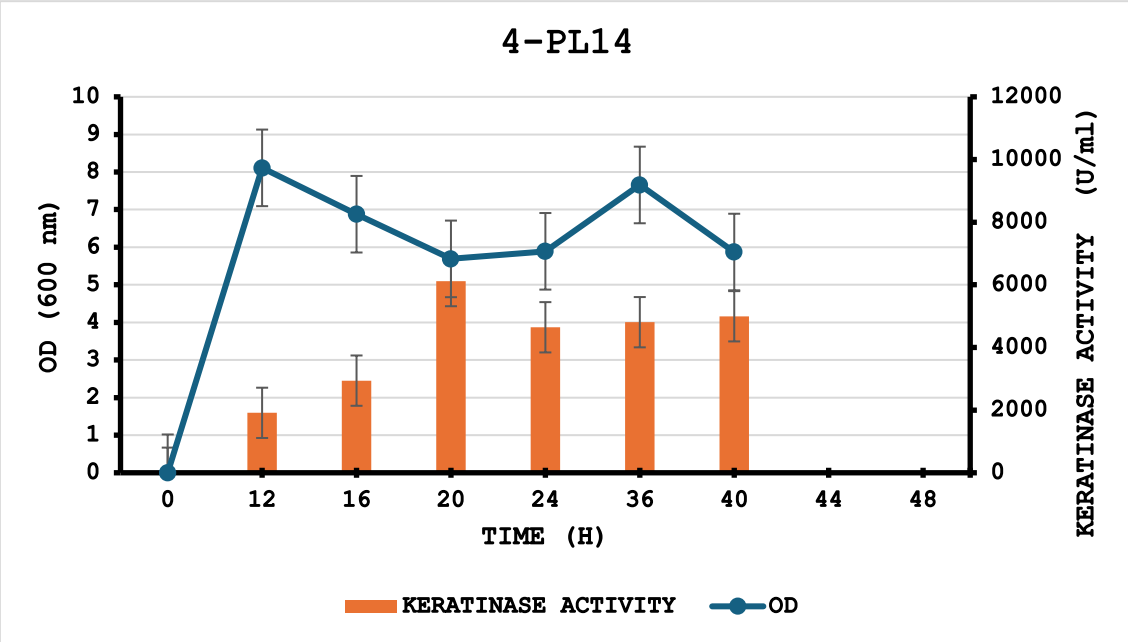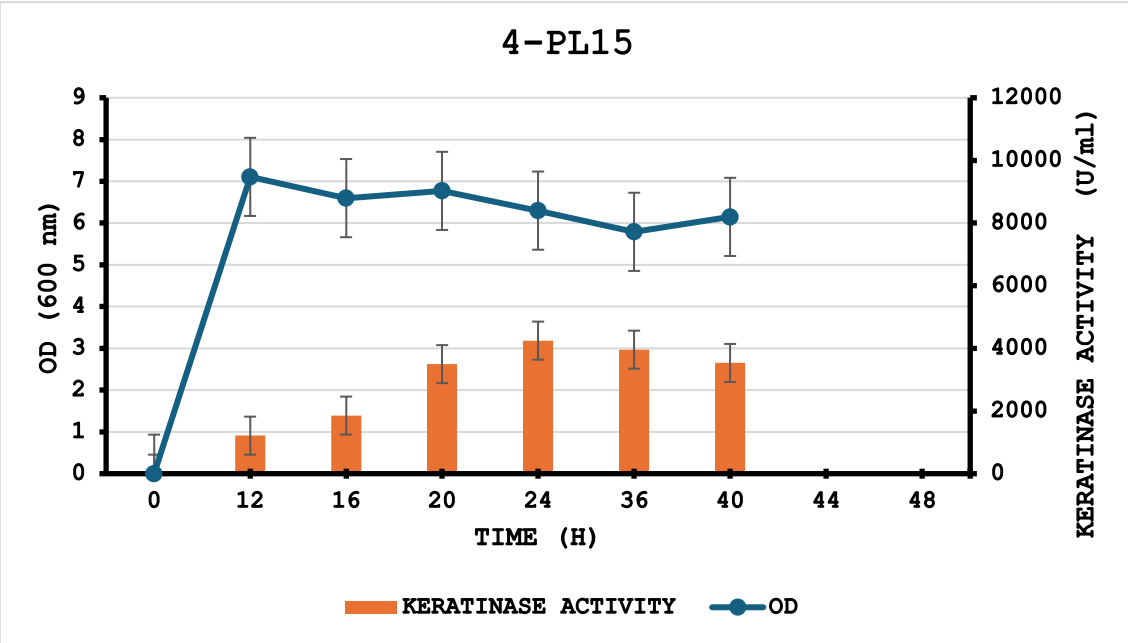

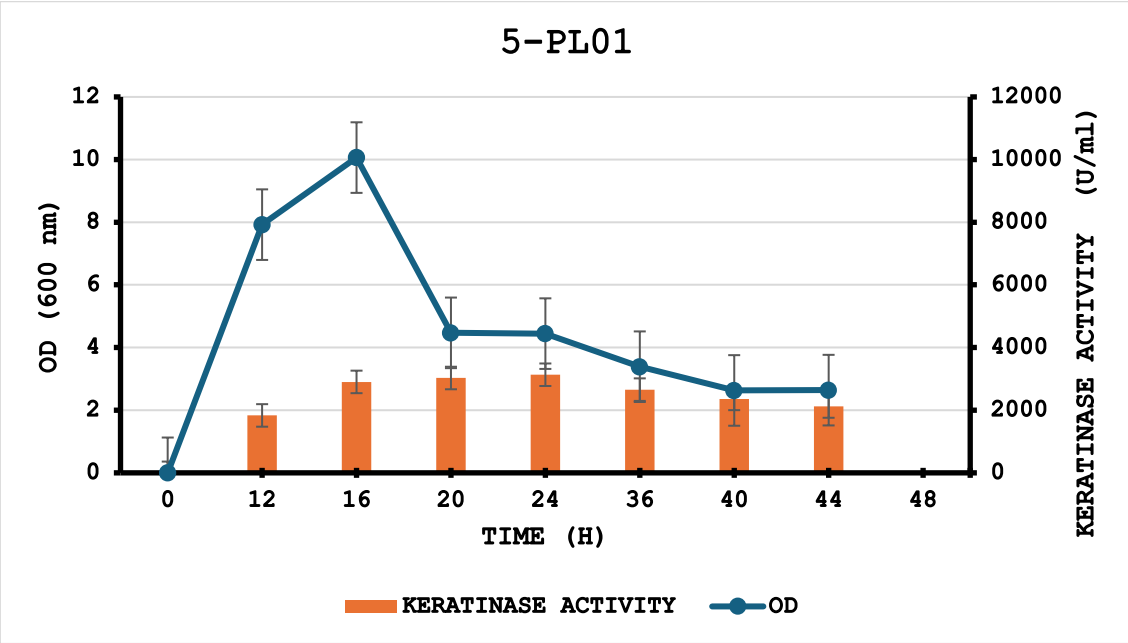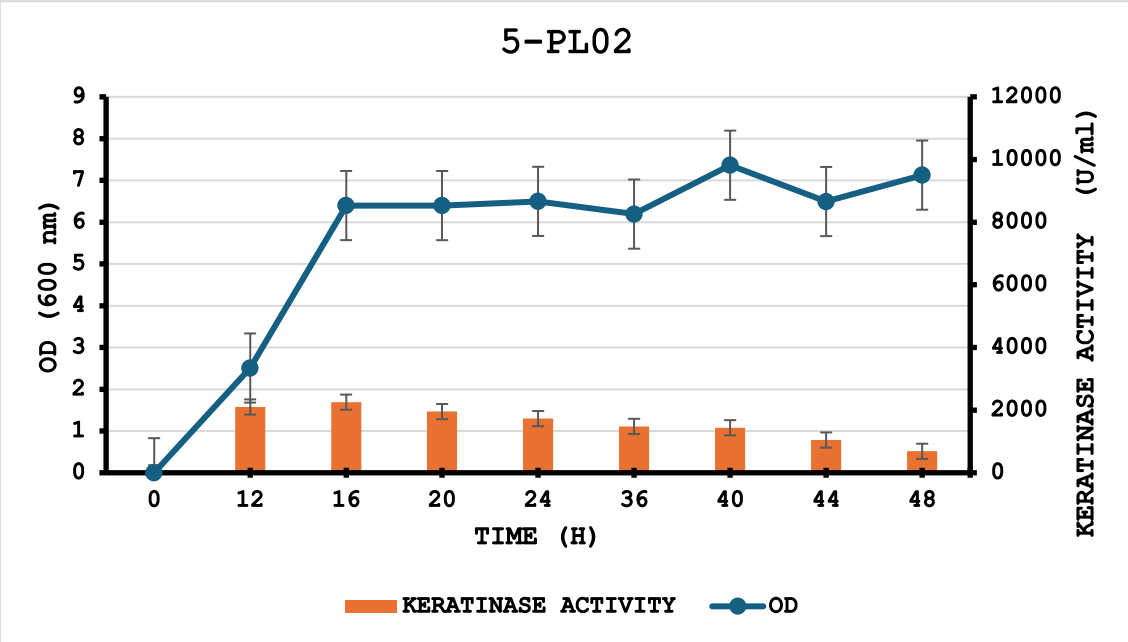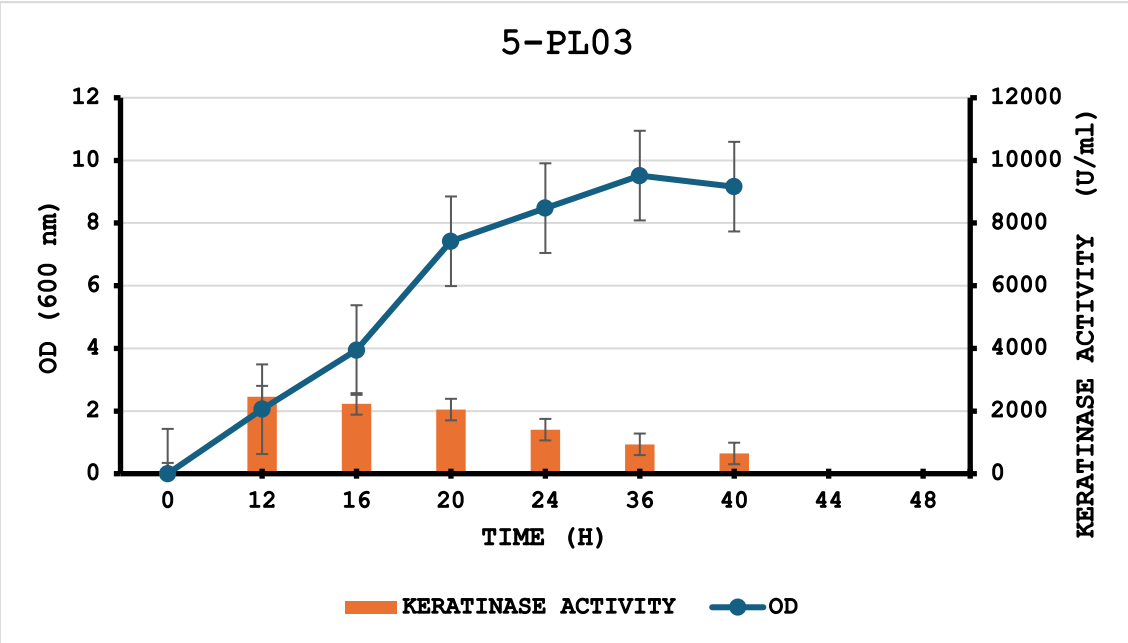

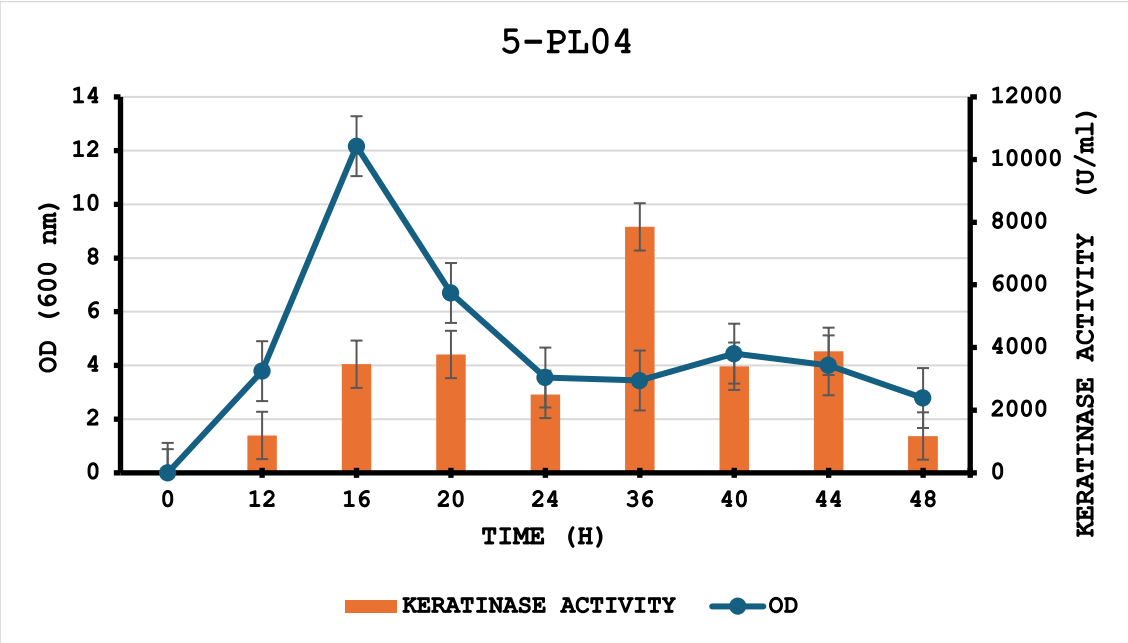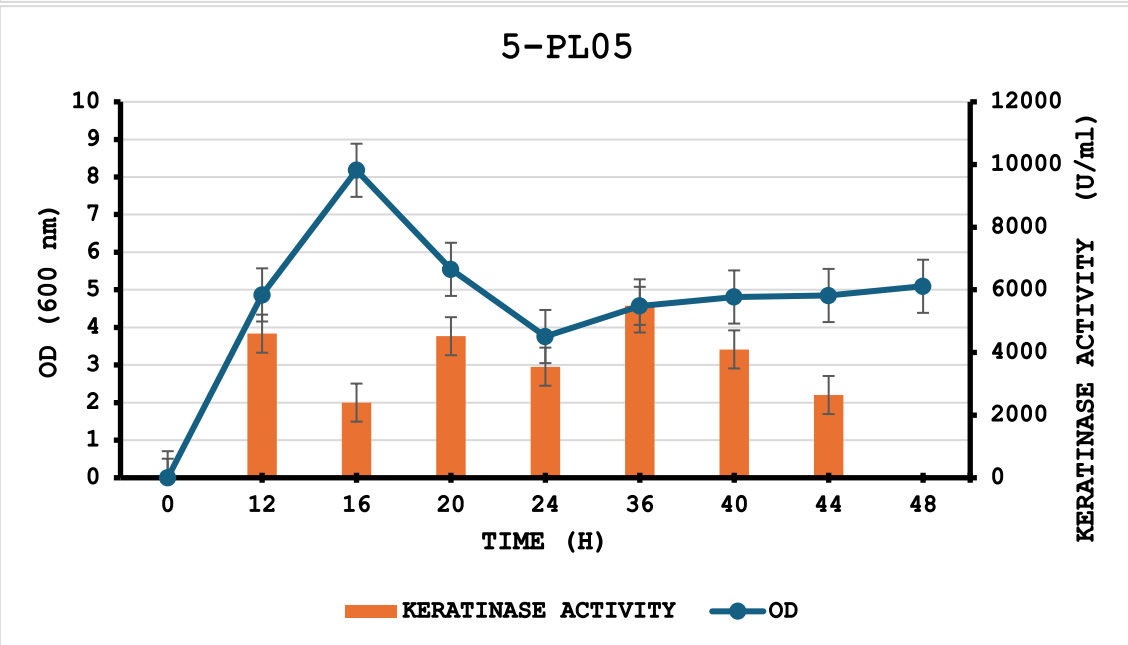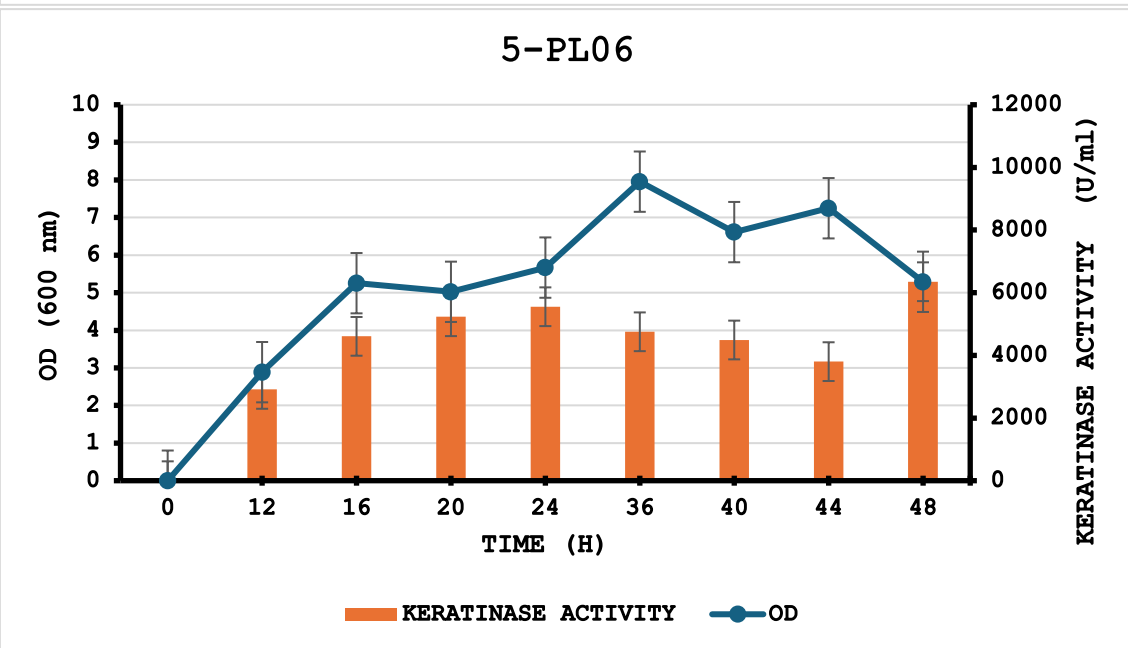

### 5-PL07

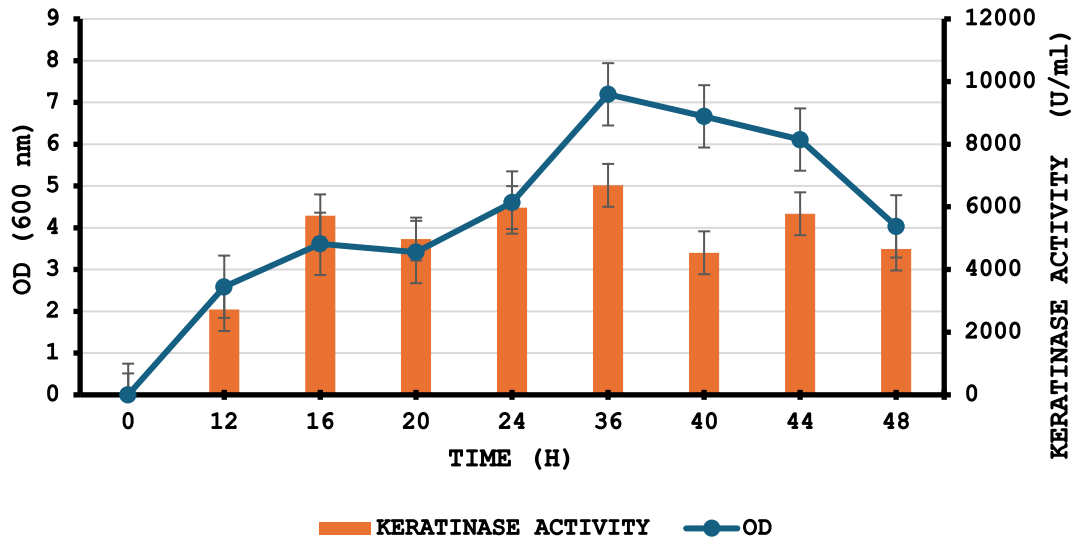

### 5-PL08

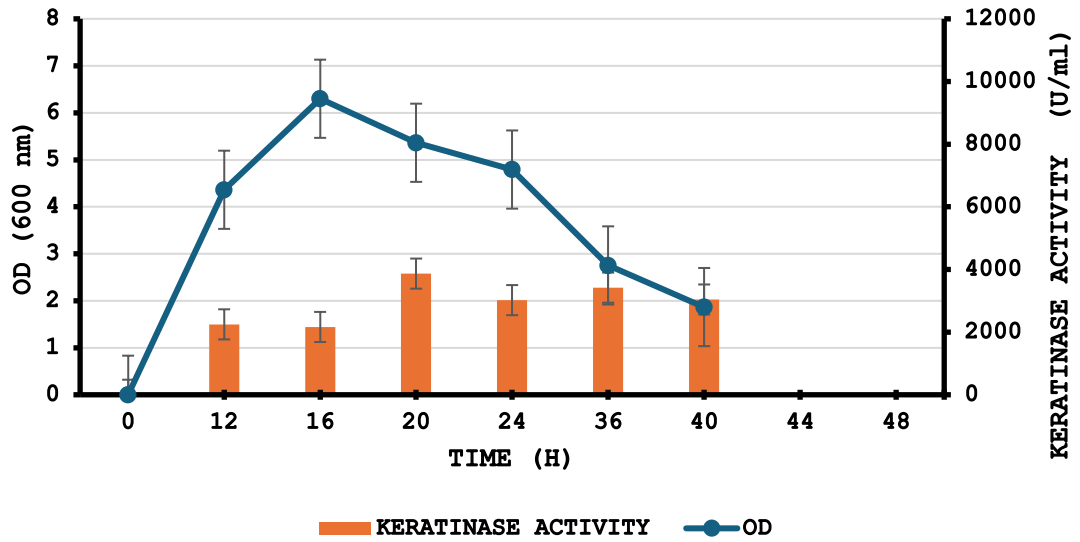

### 5-PL09

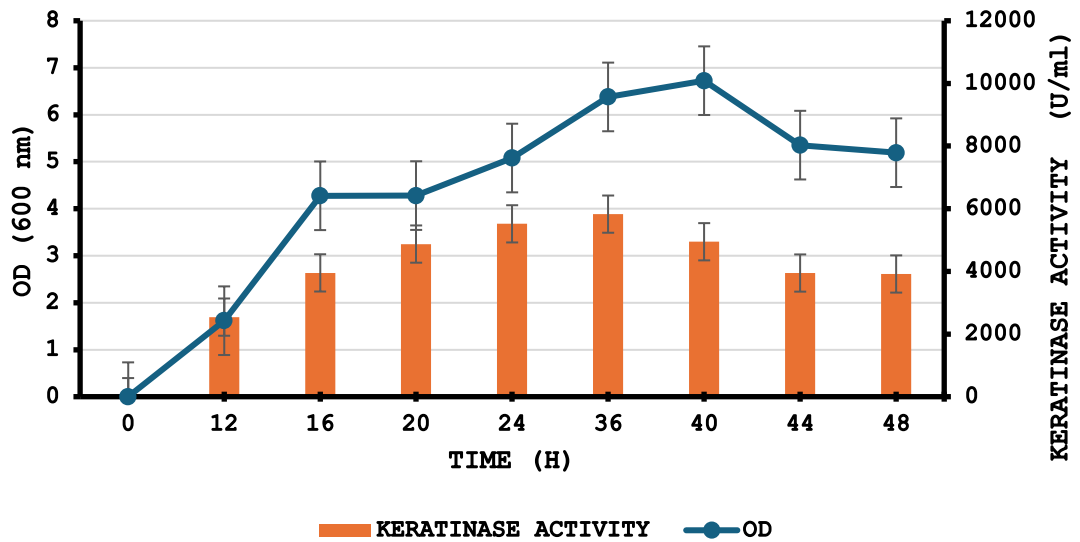

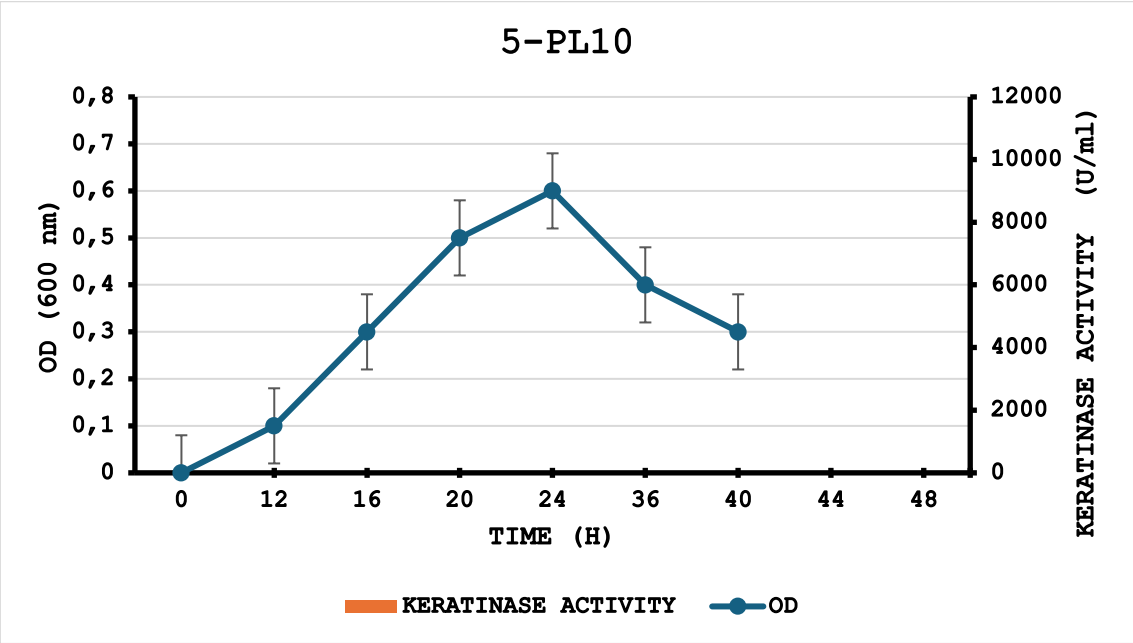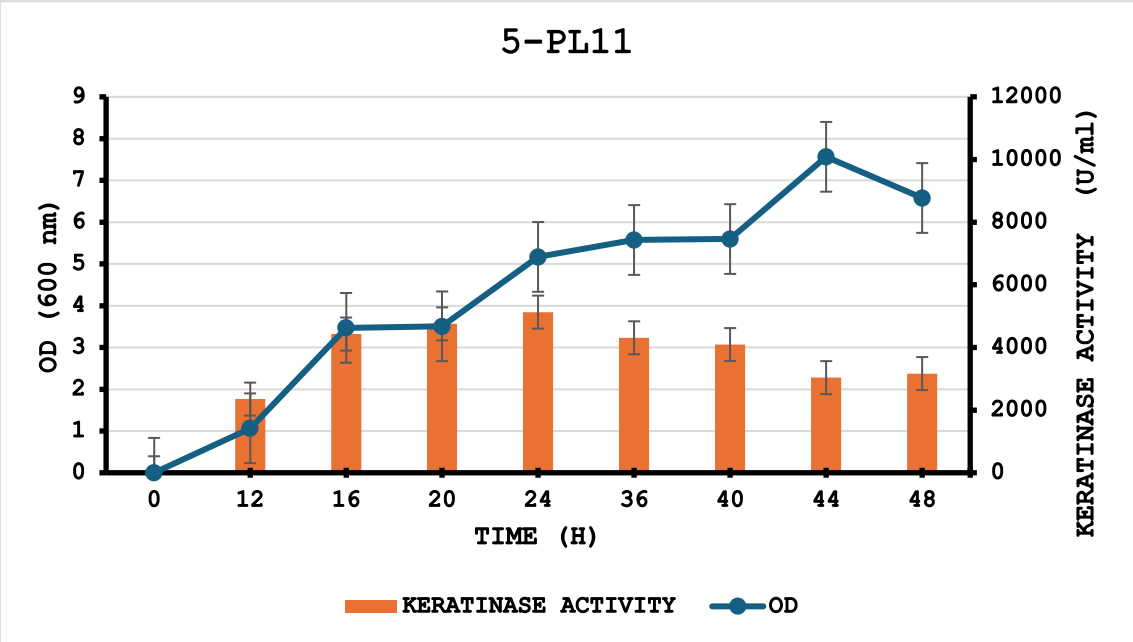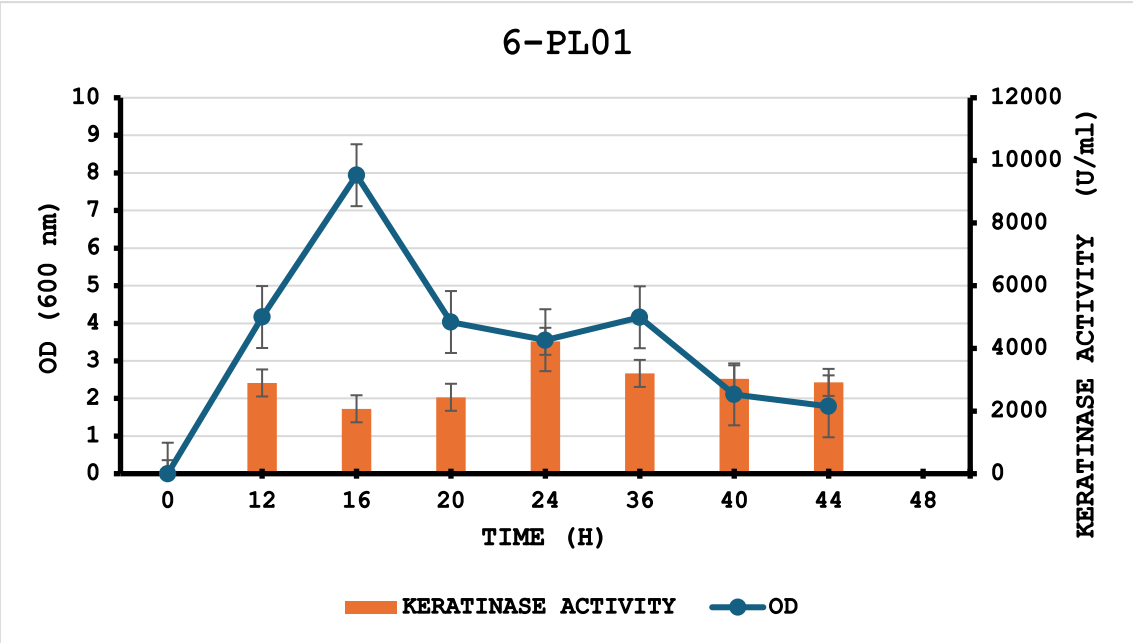

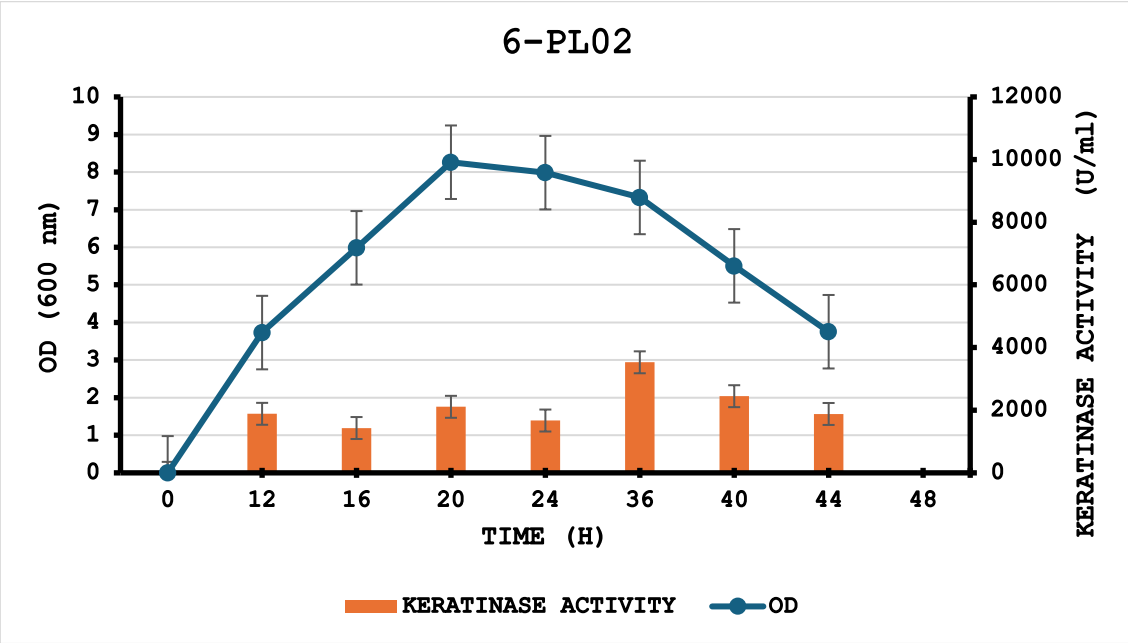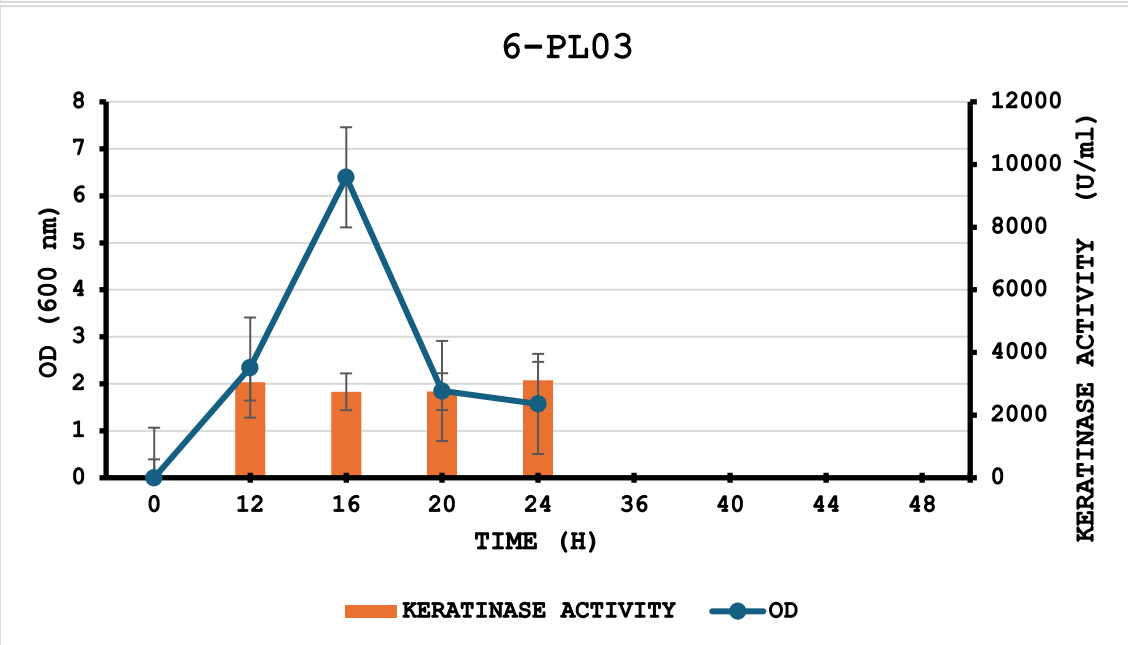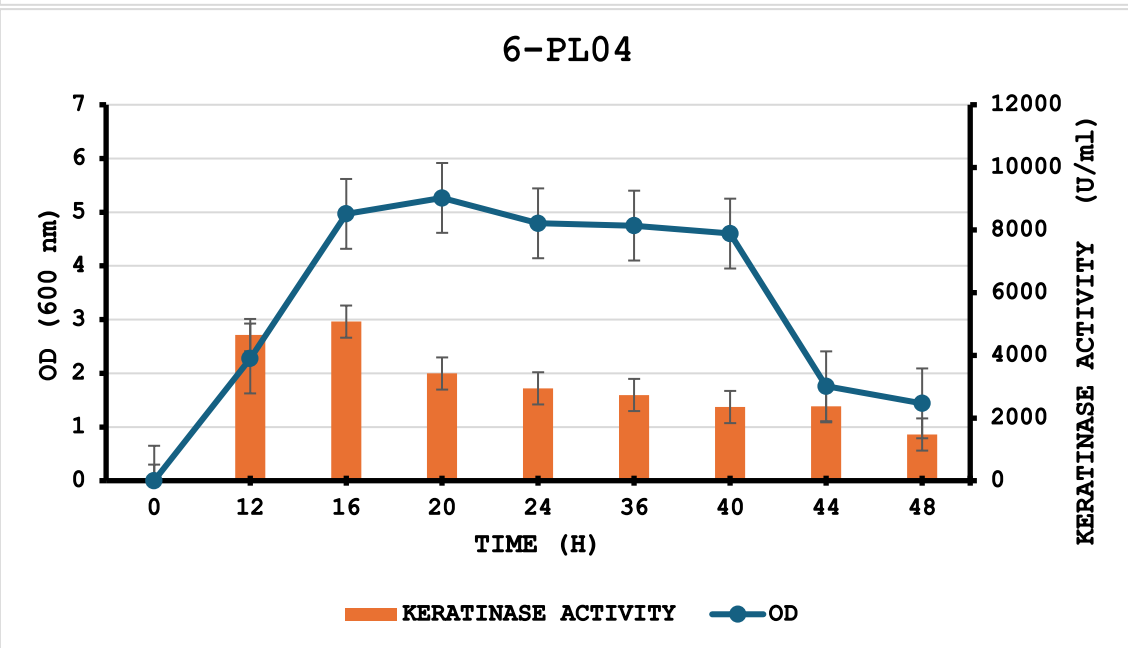

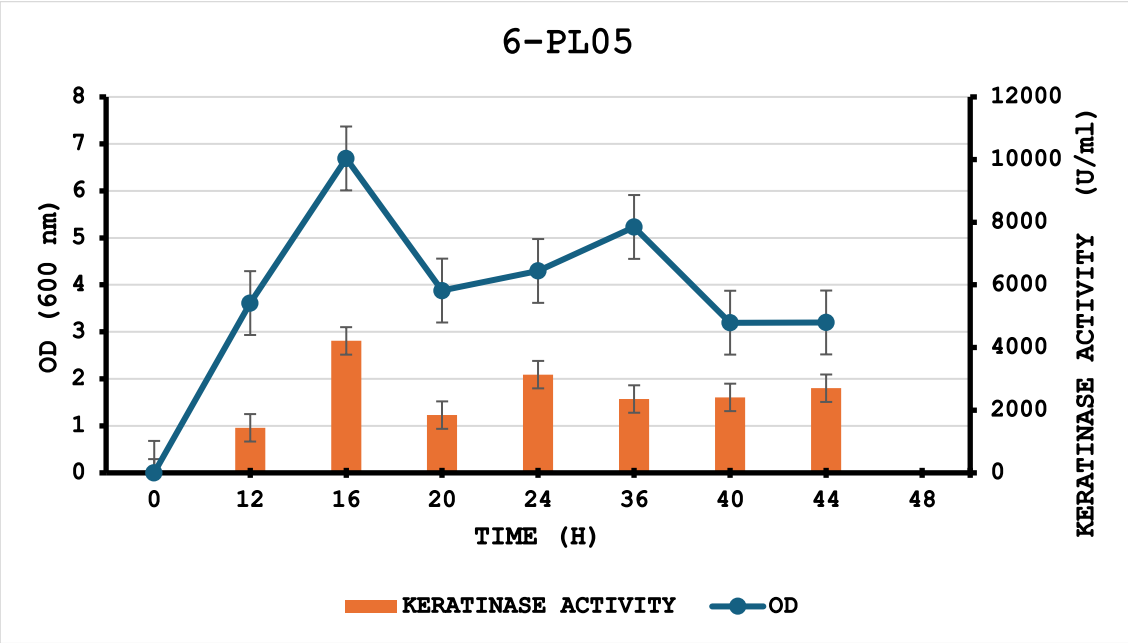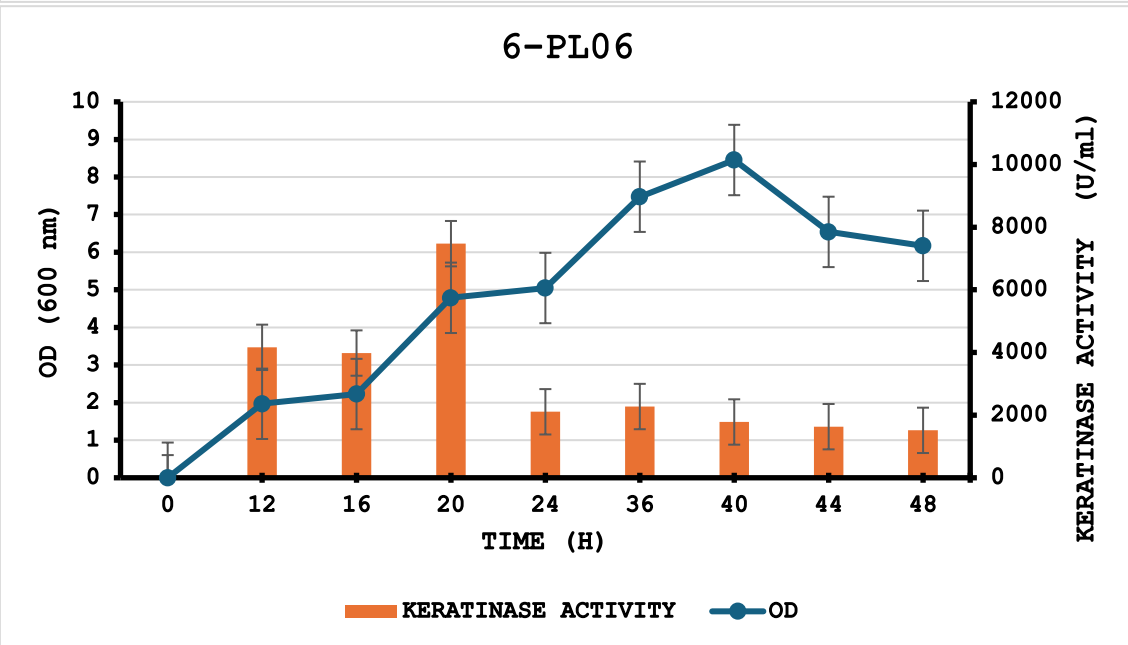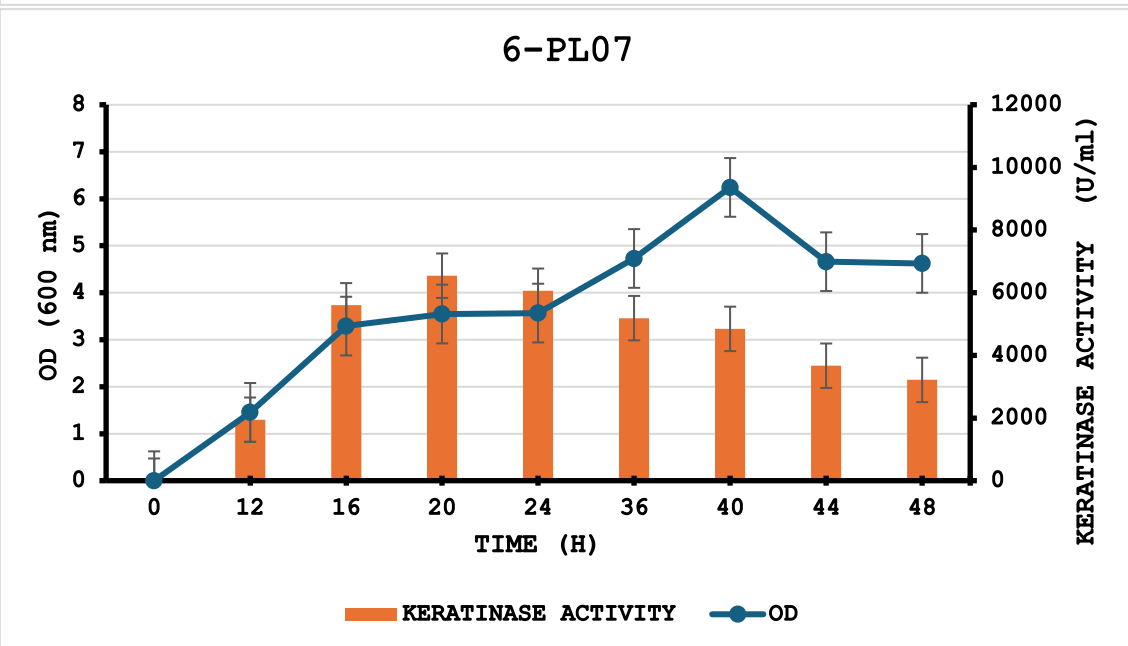

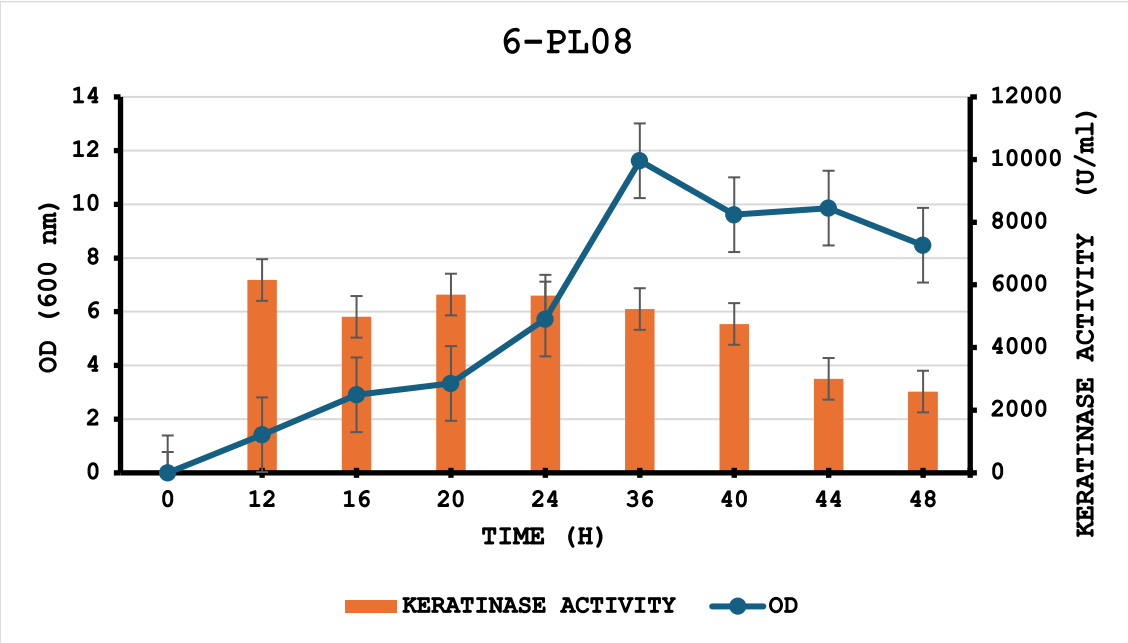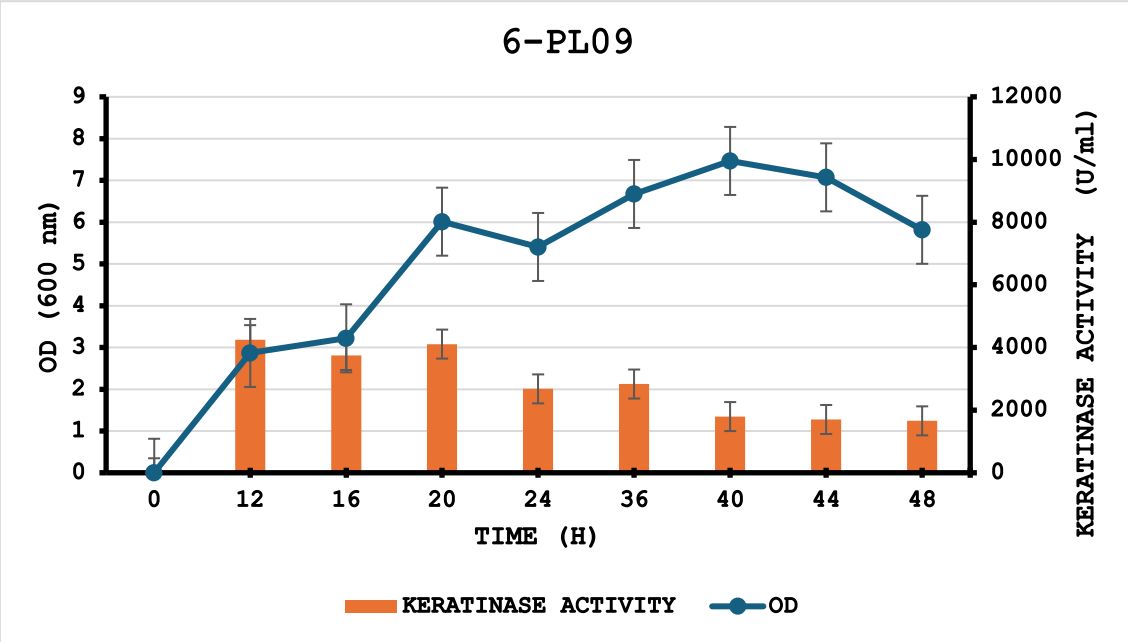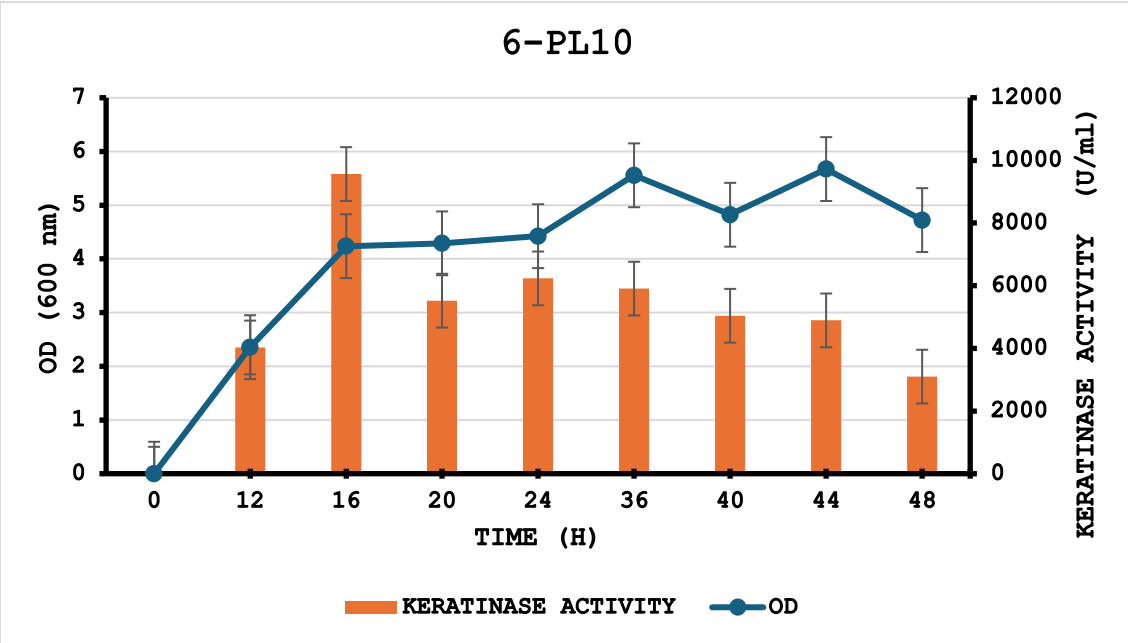

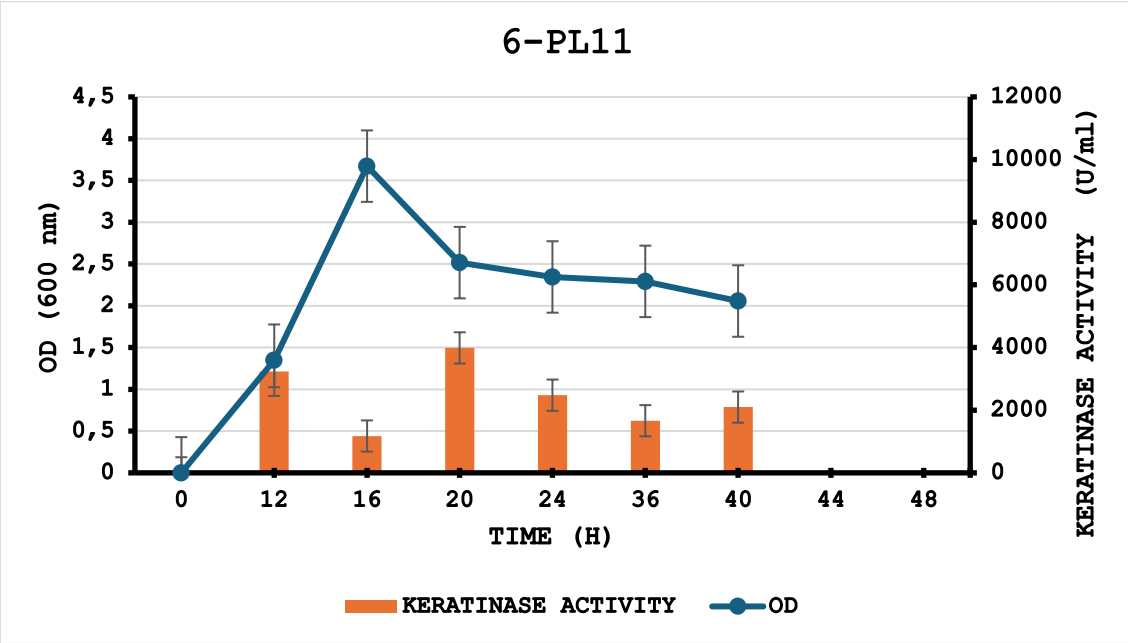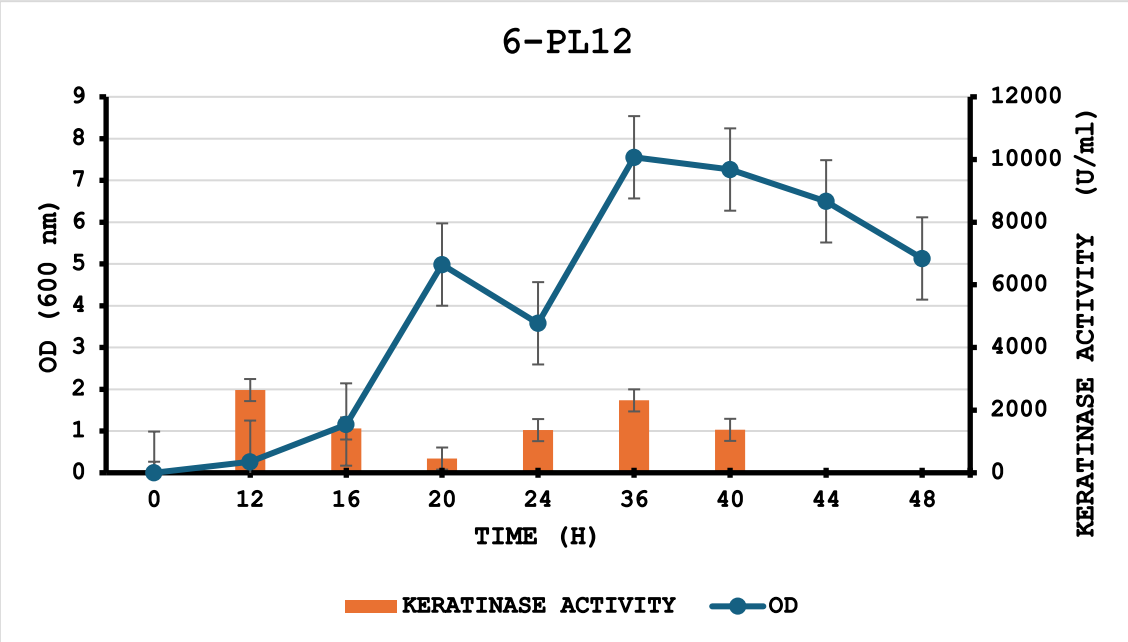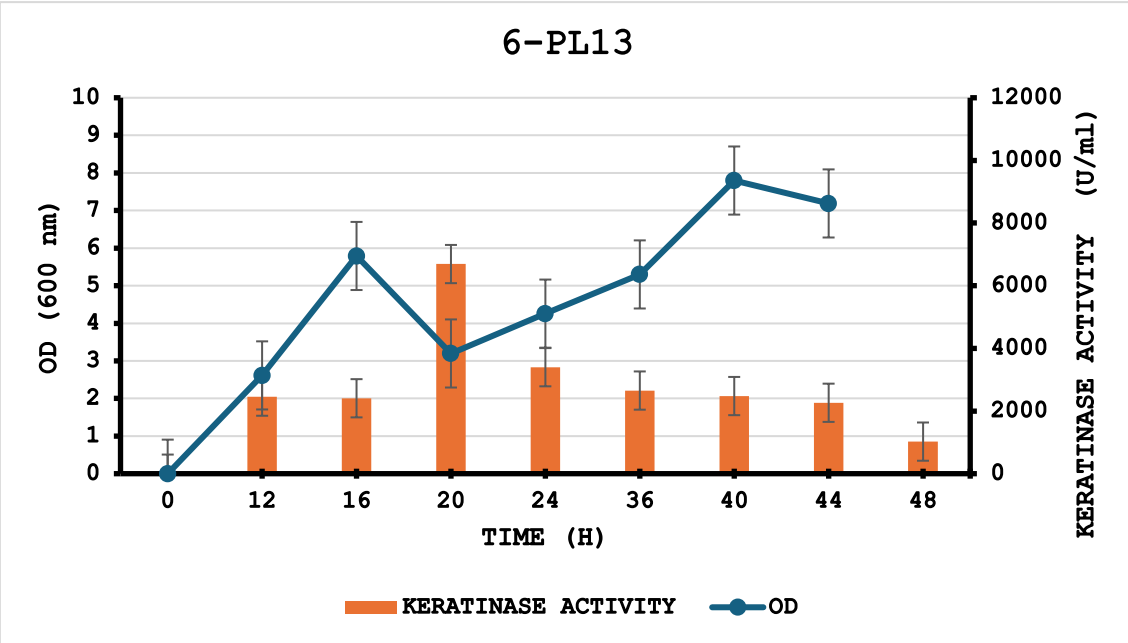

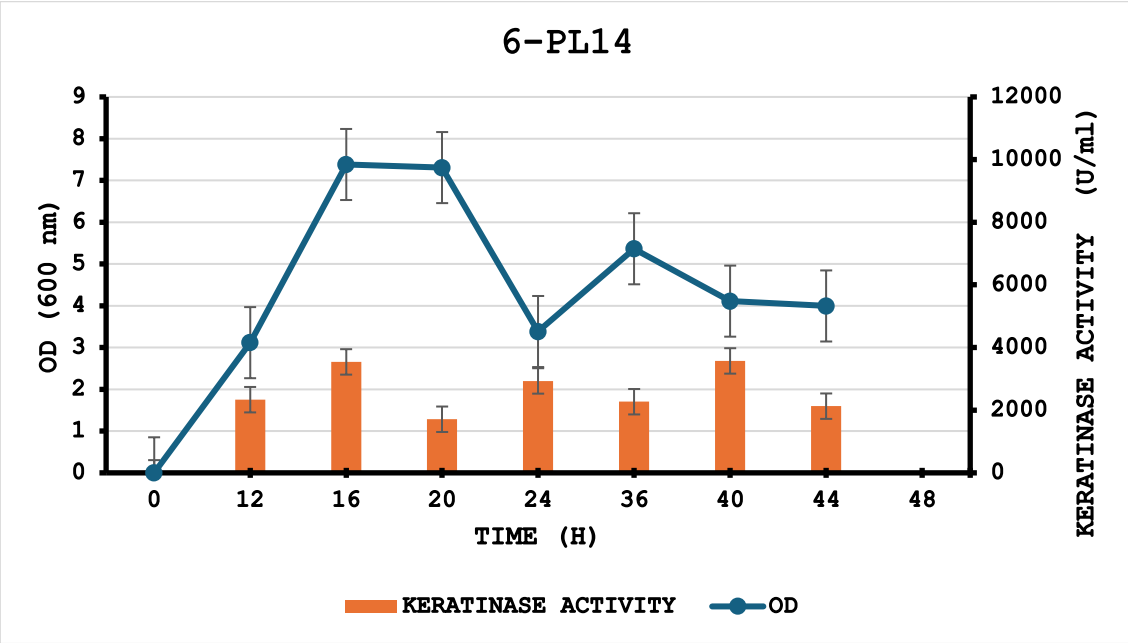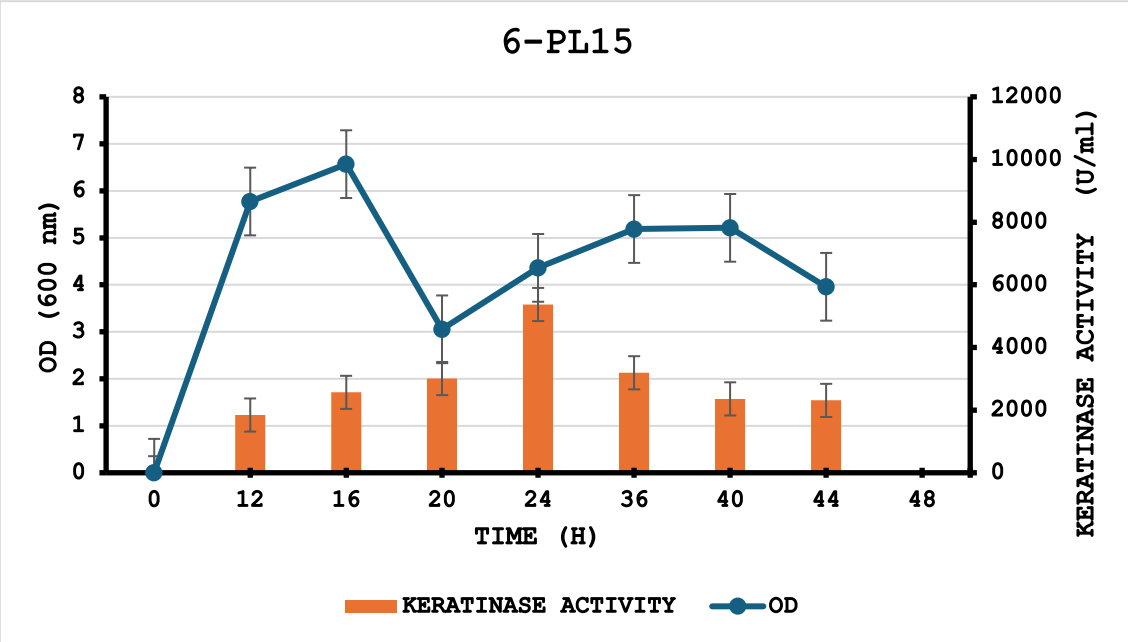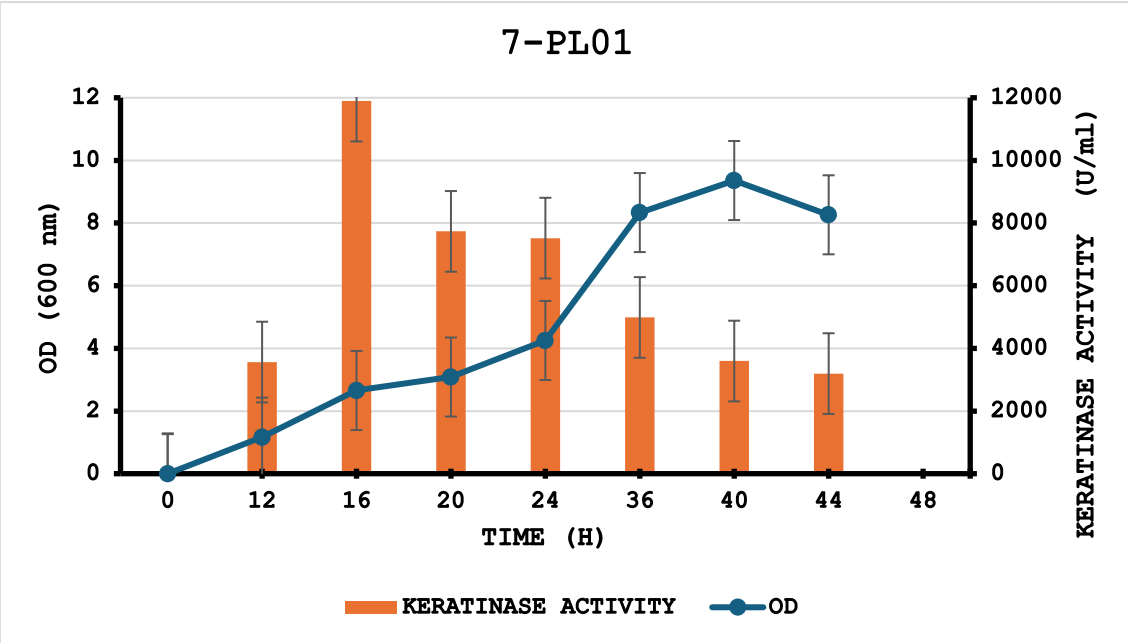

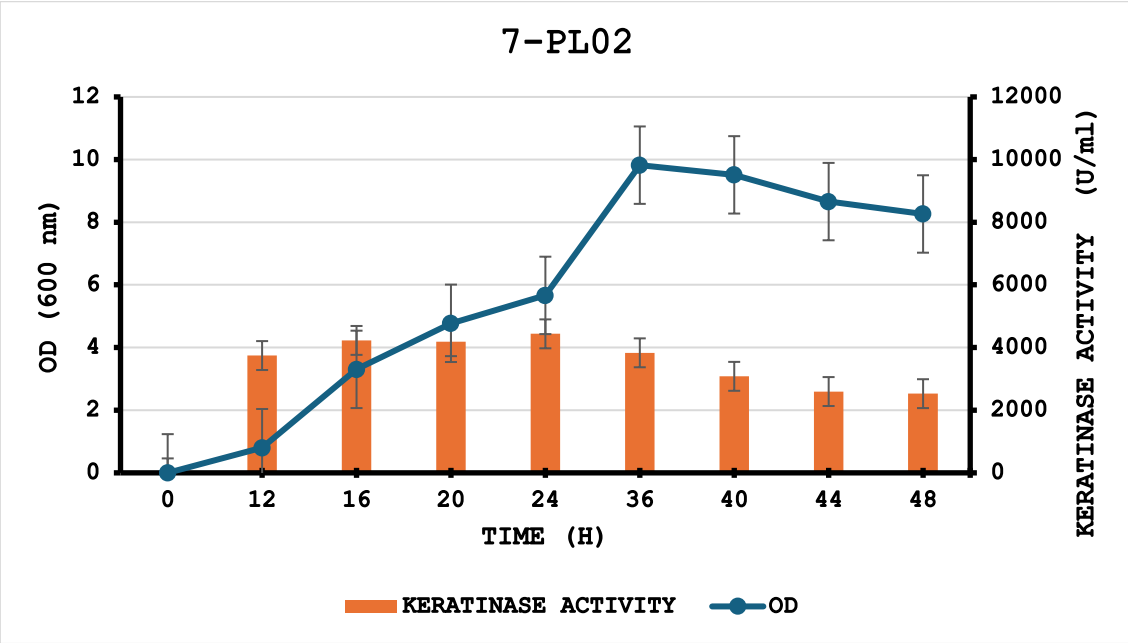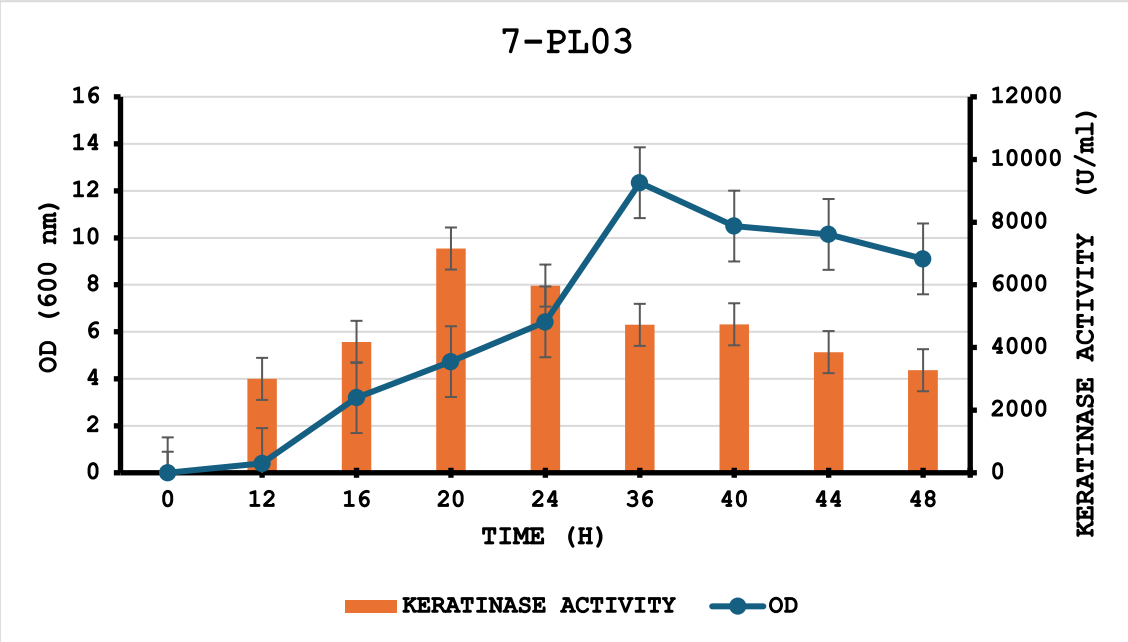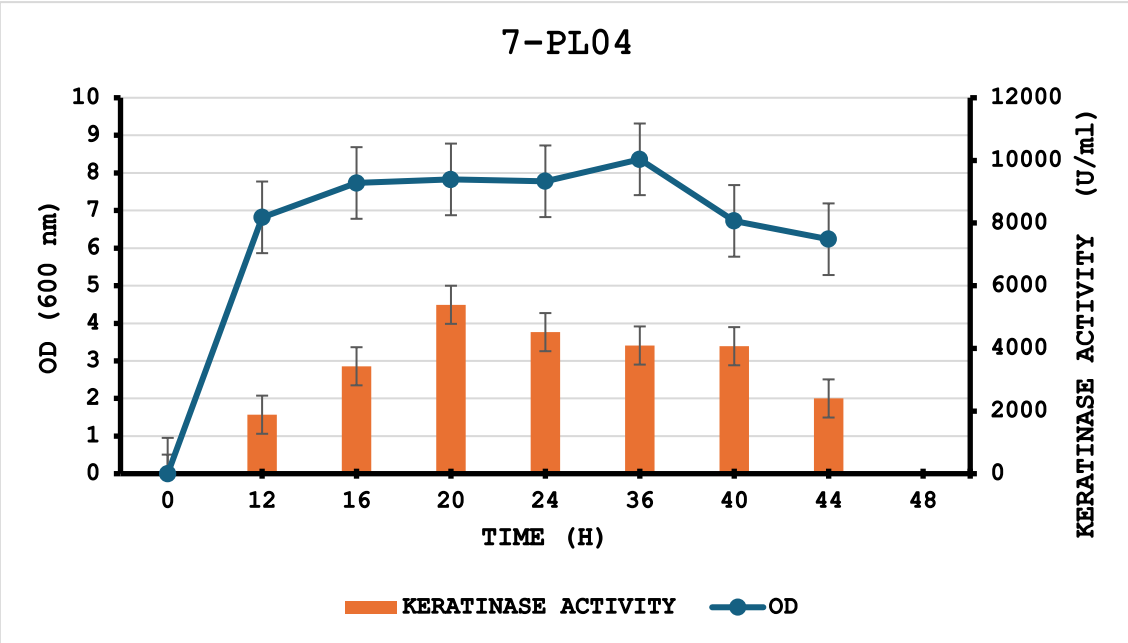

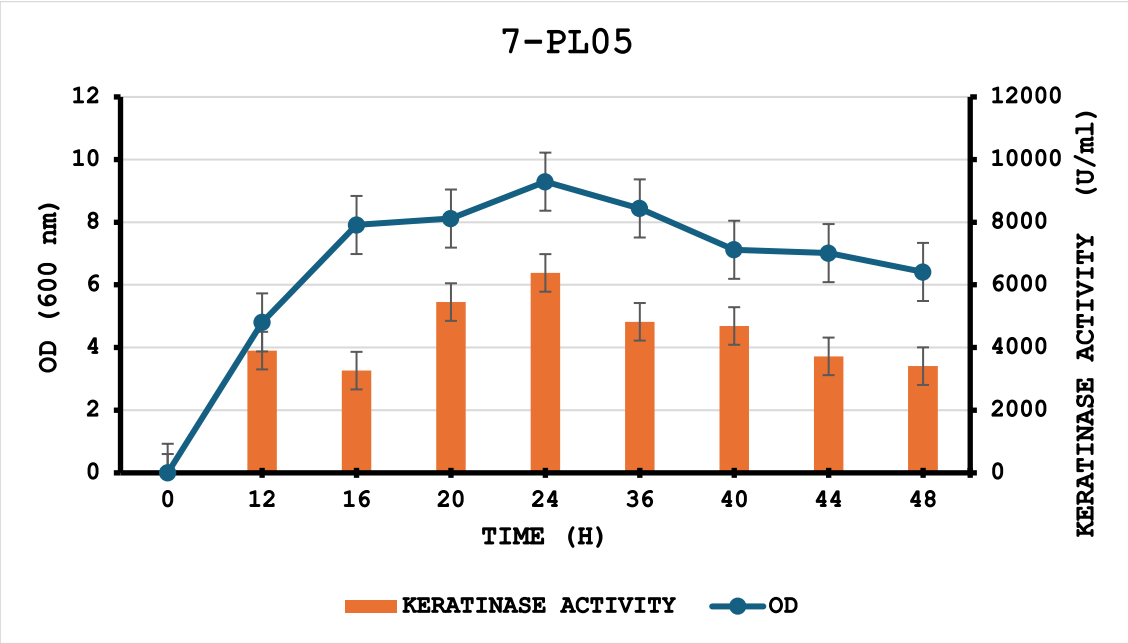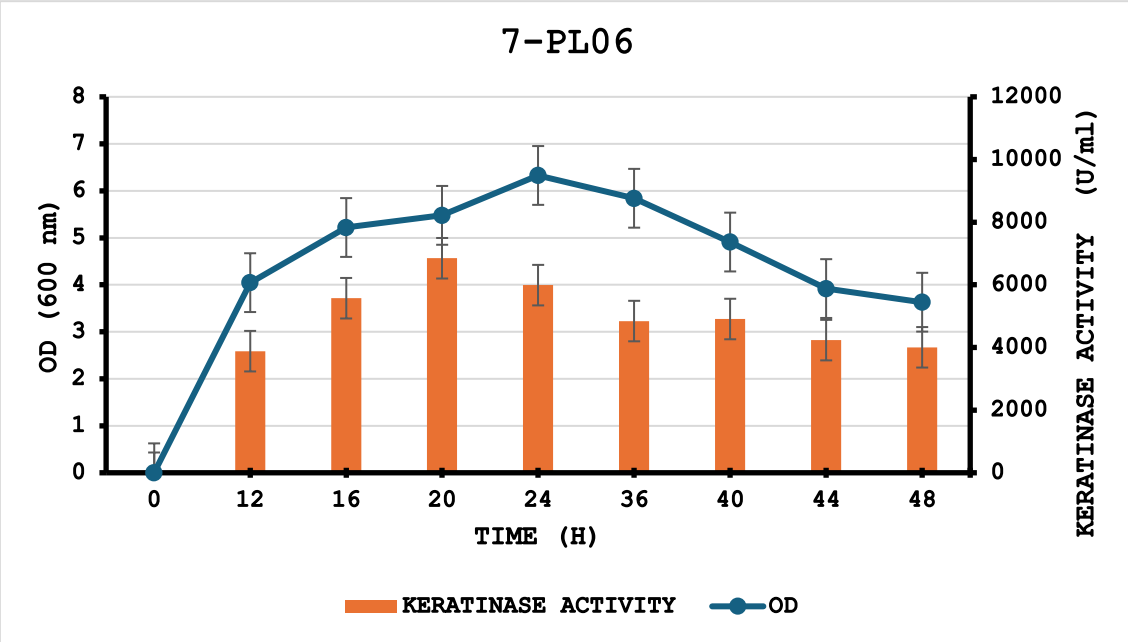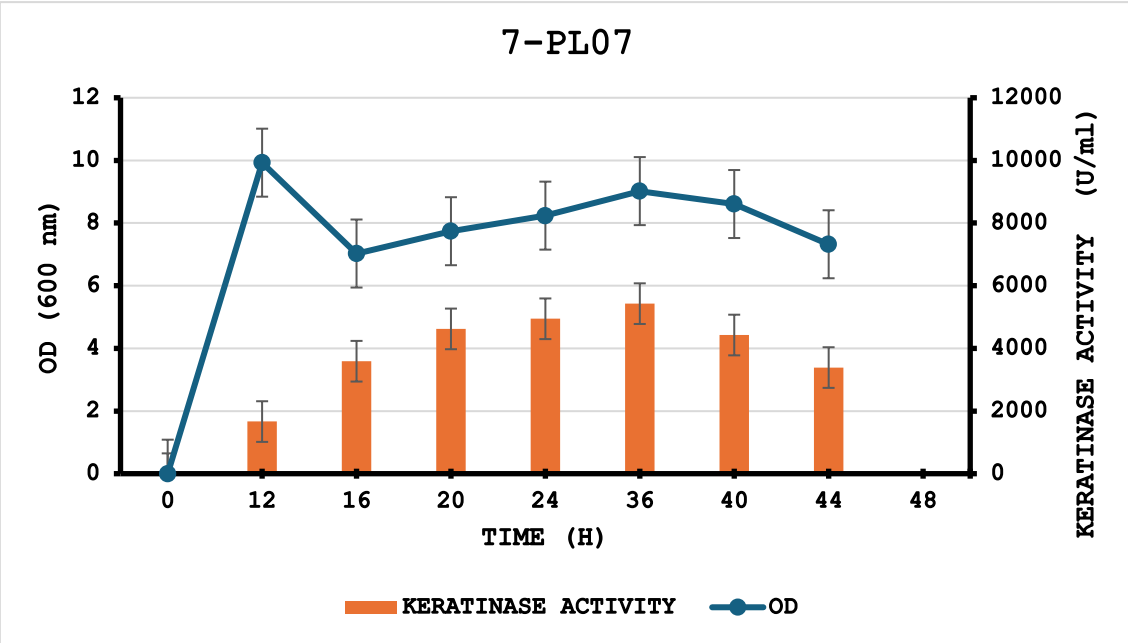

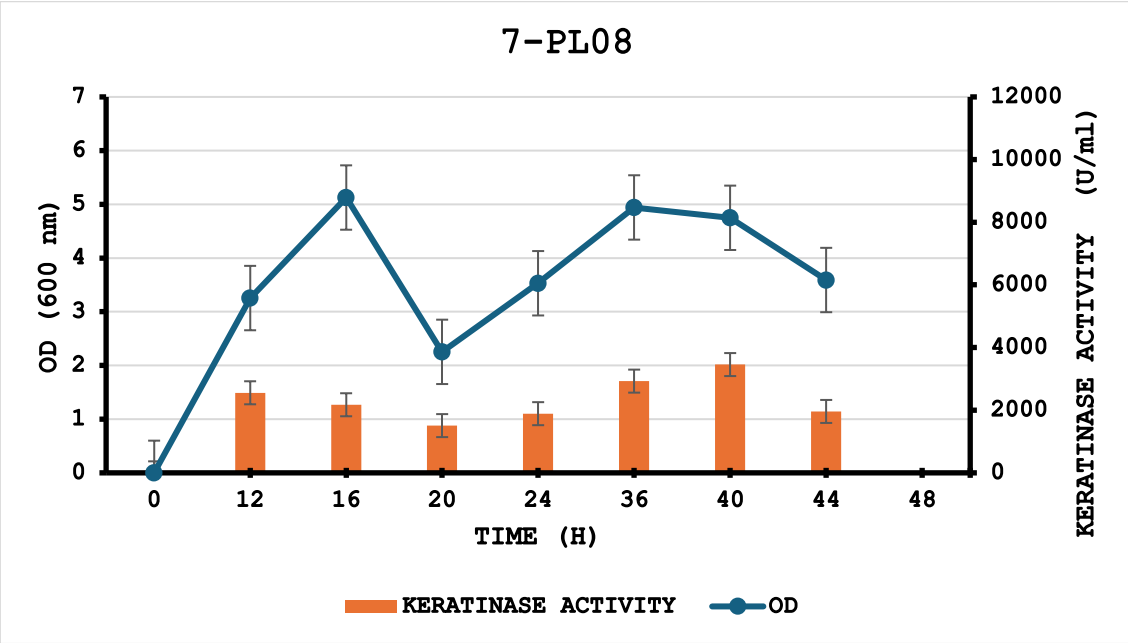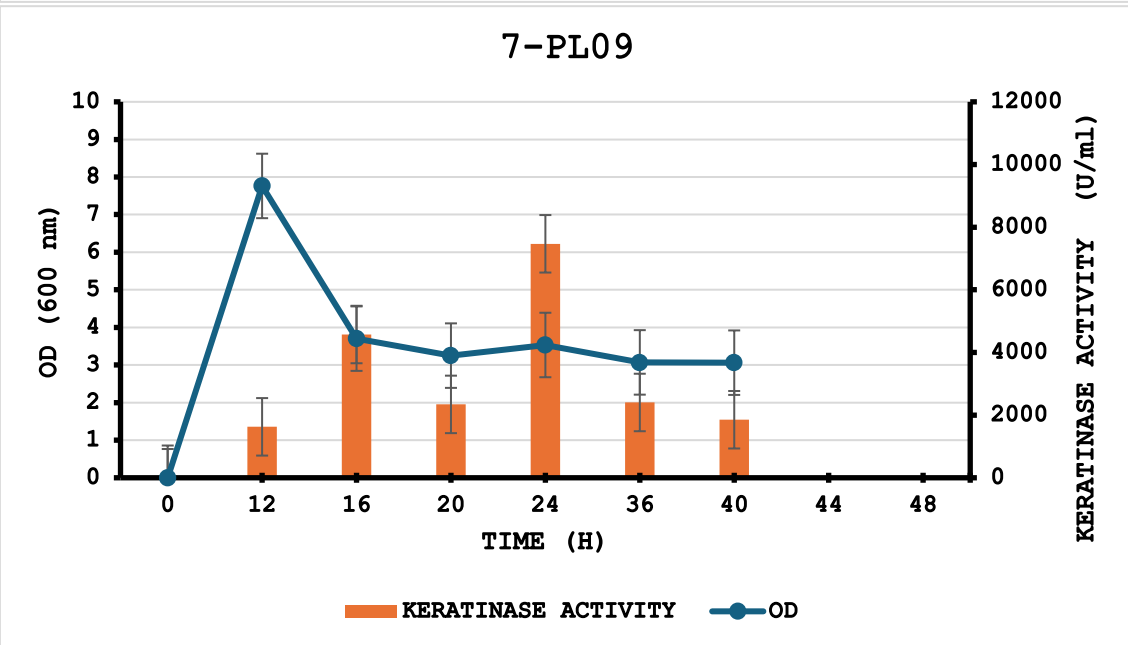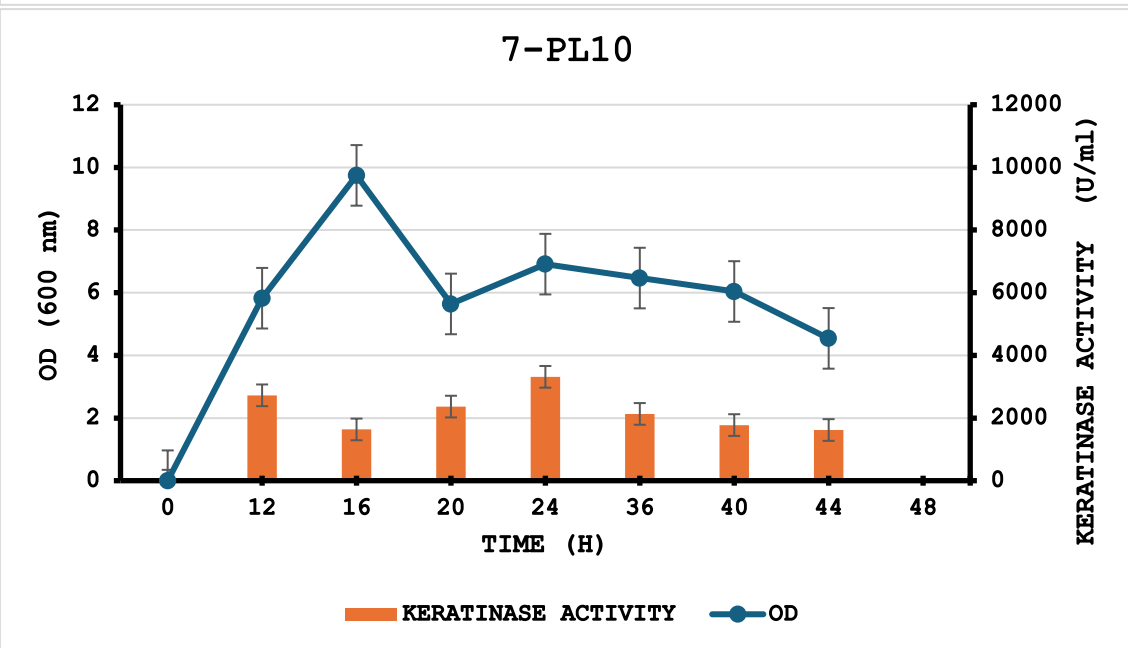

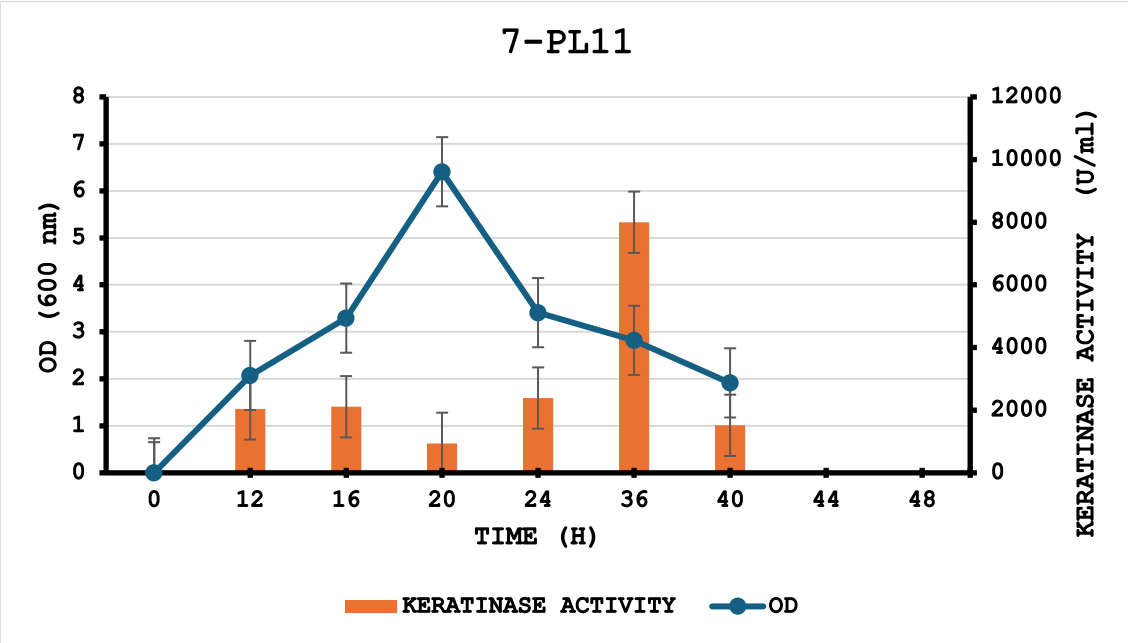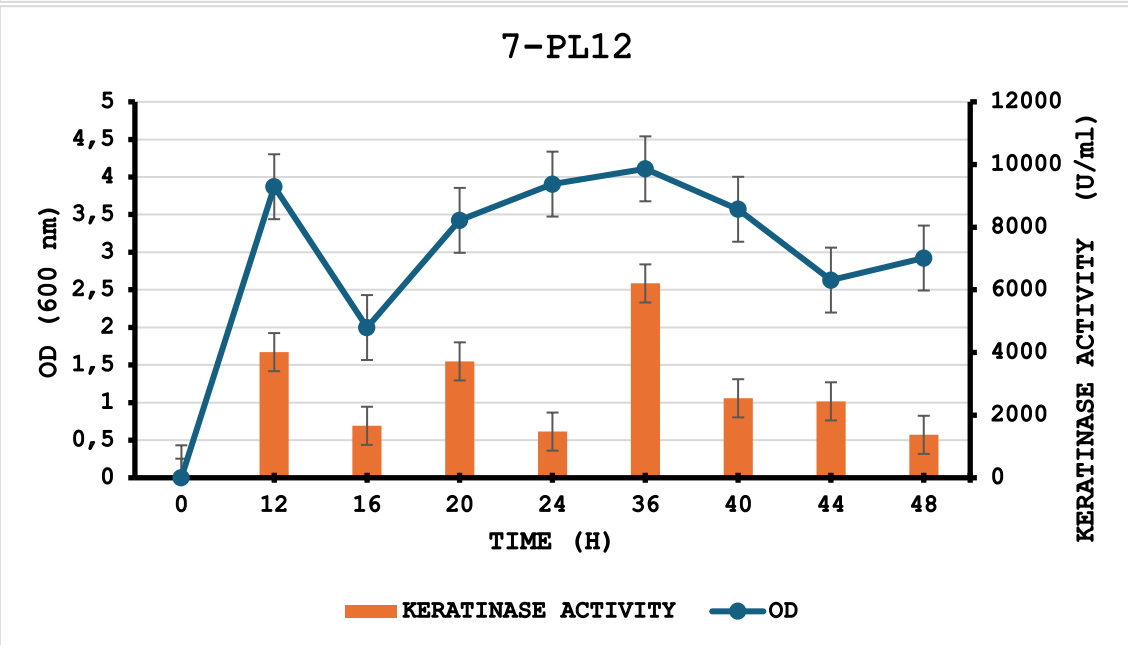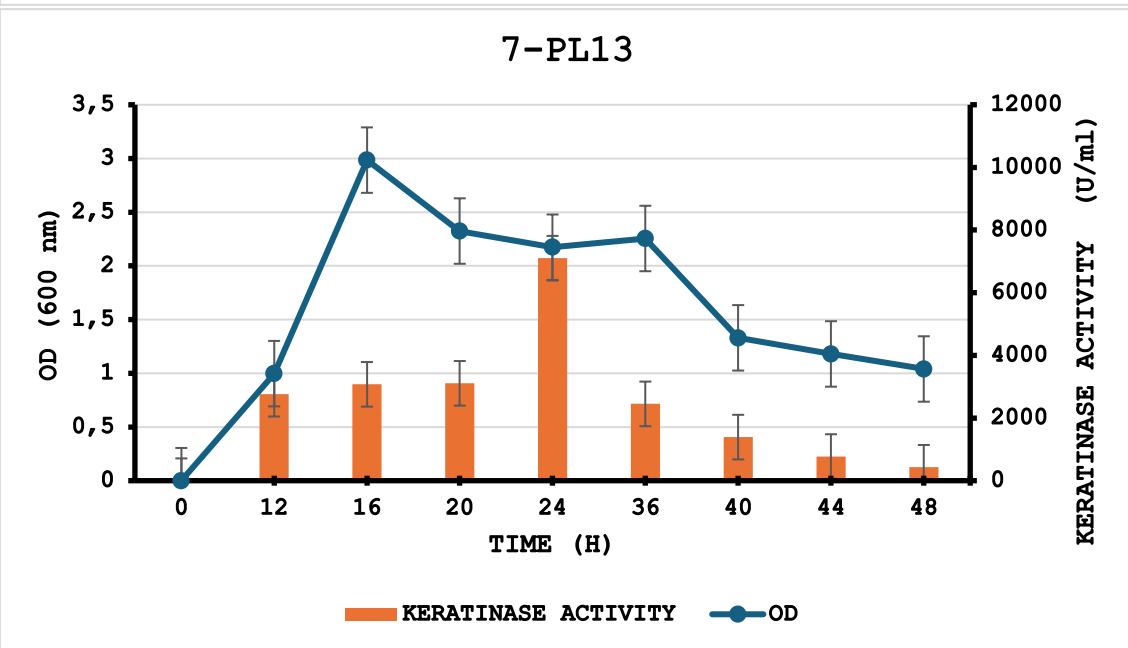

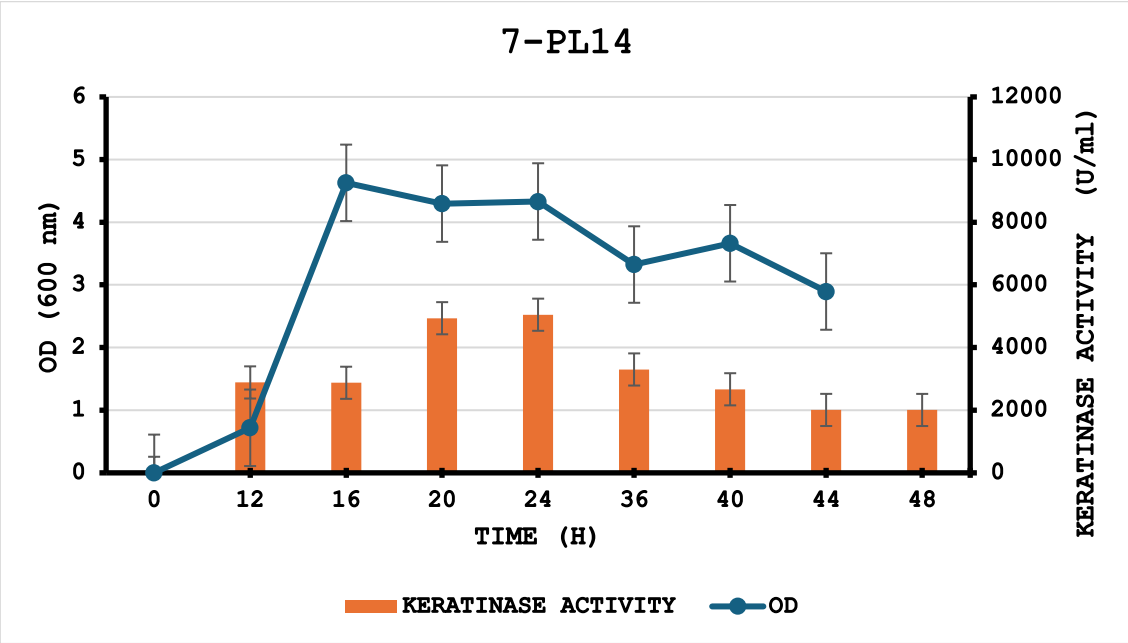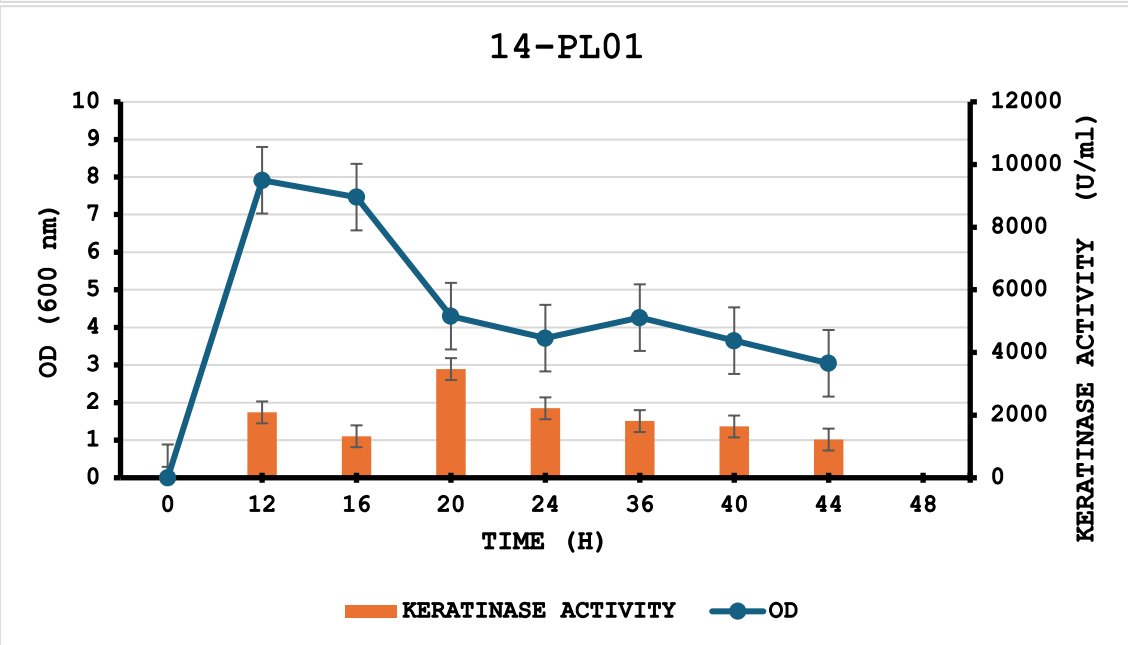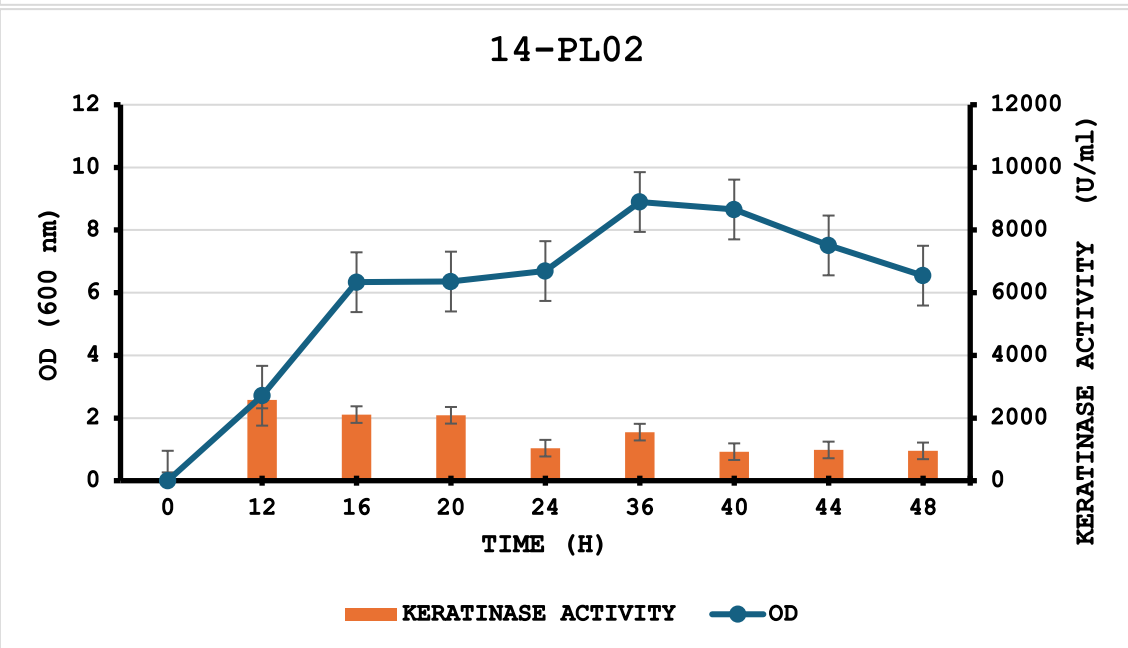

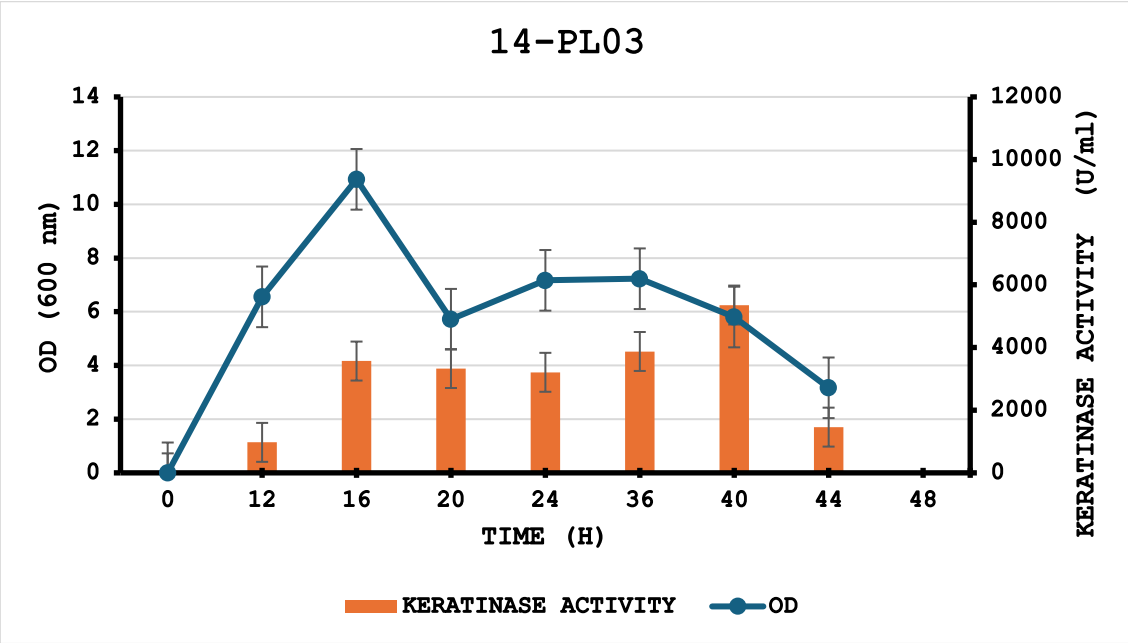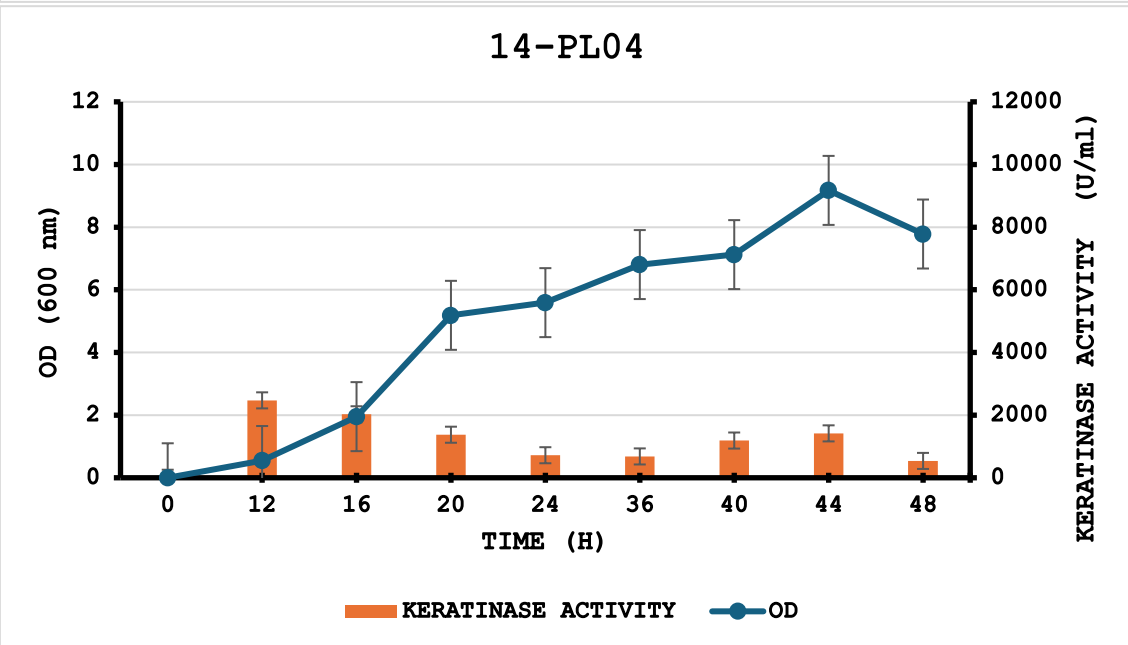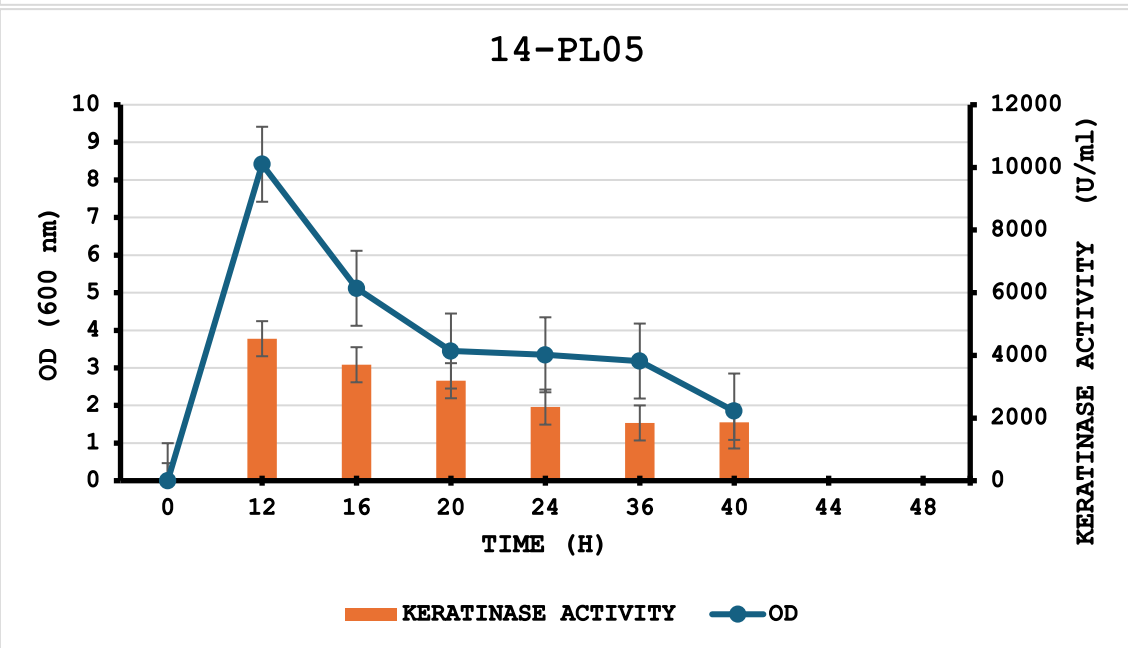

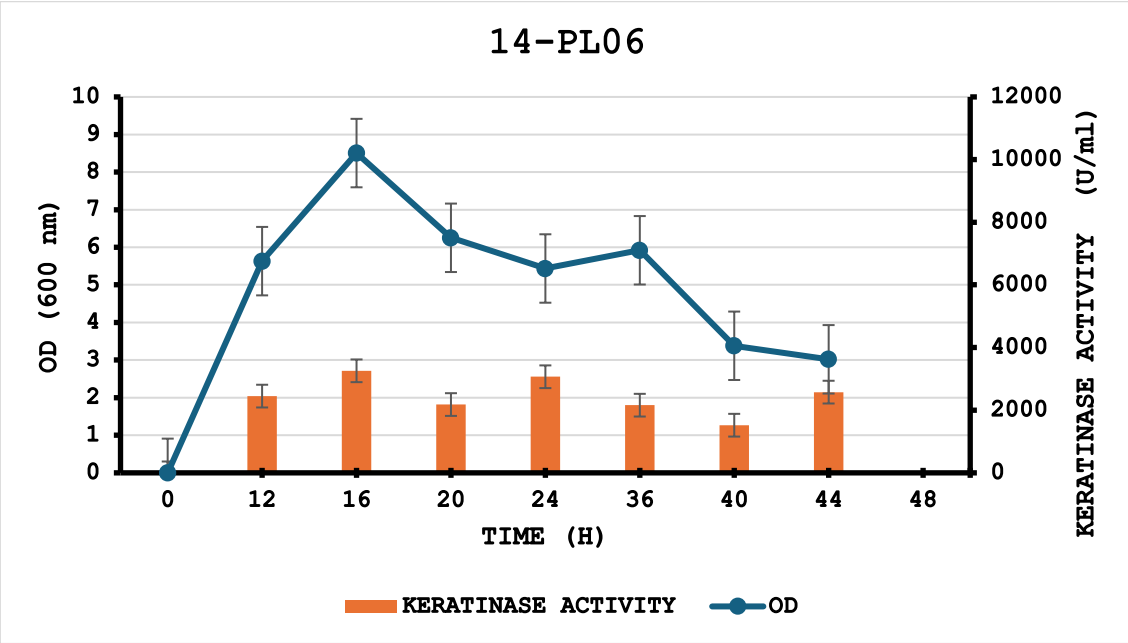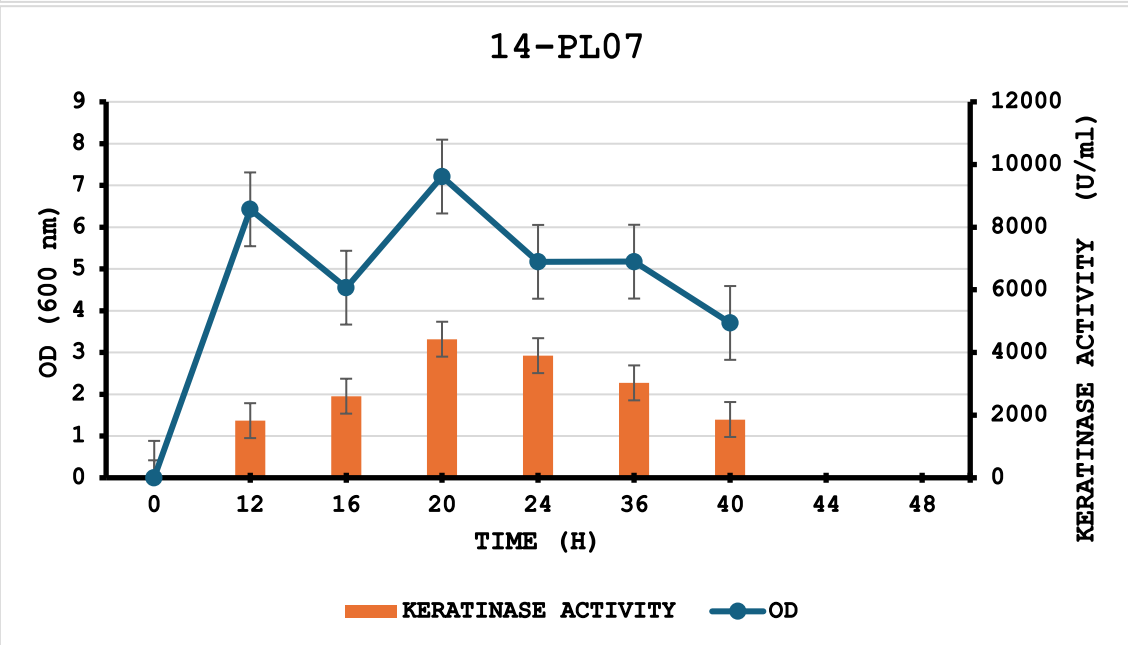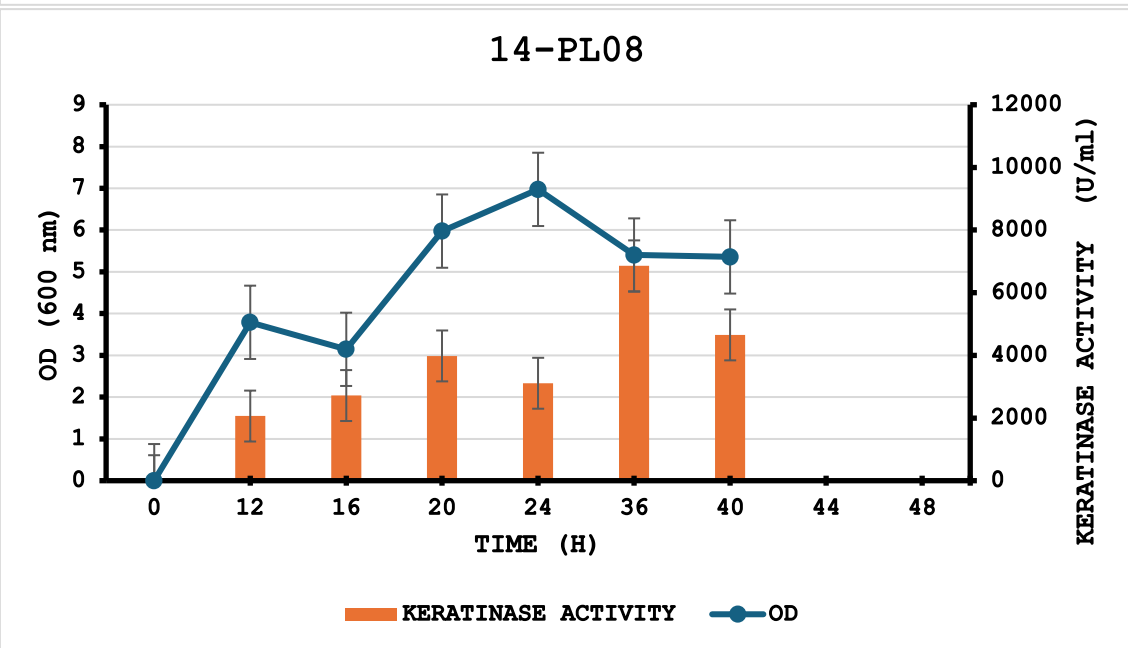

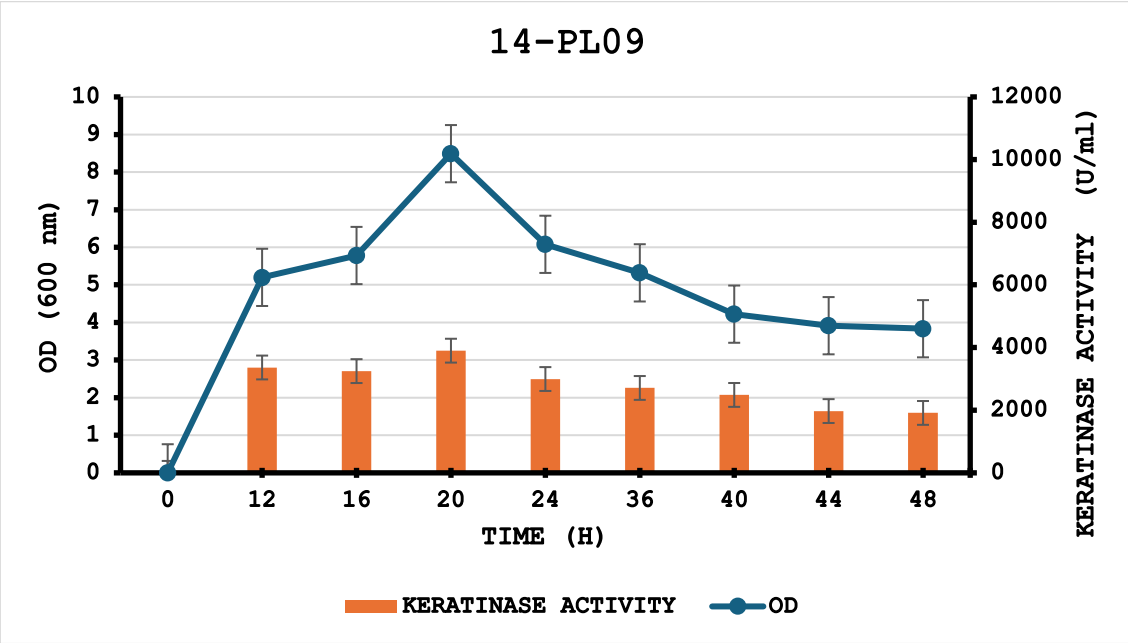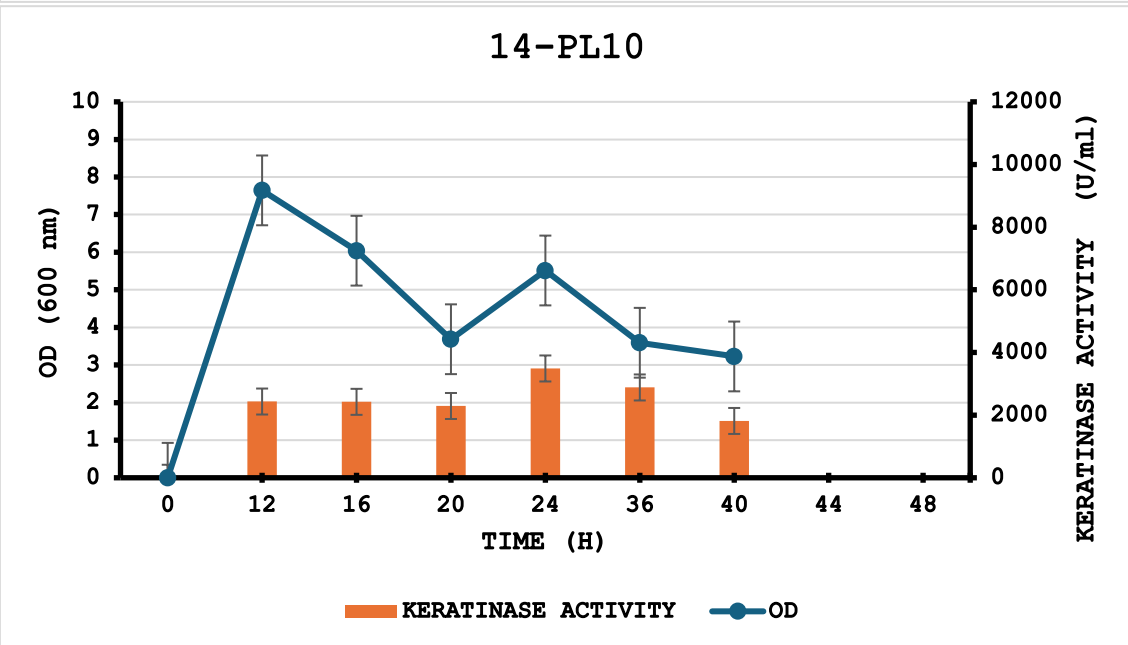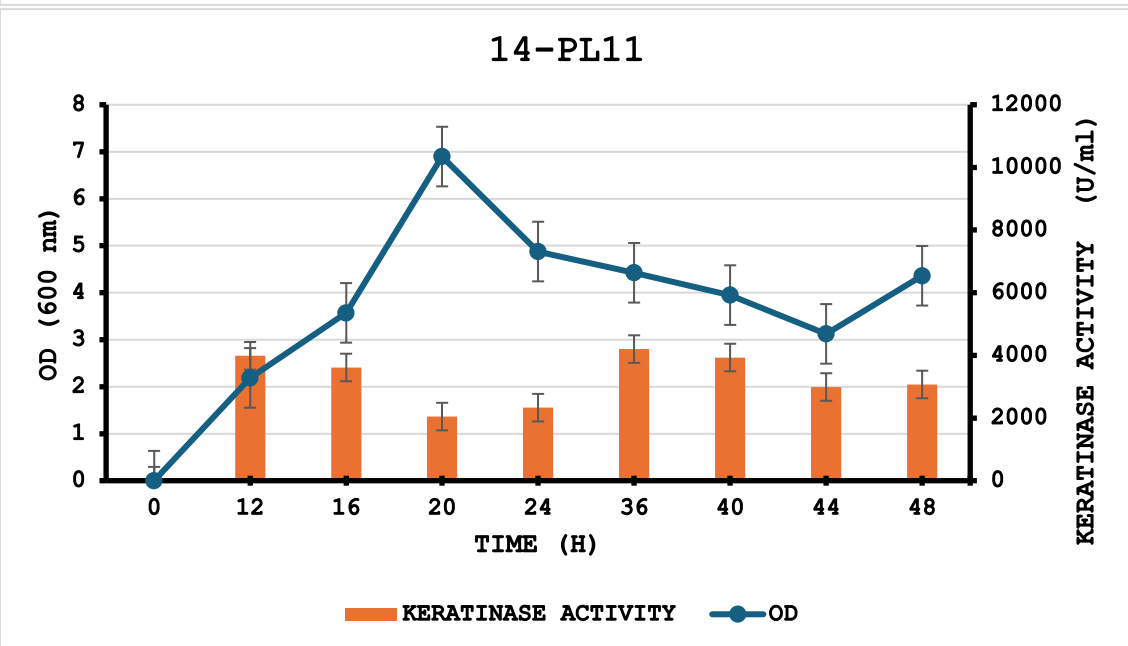

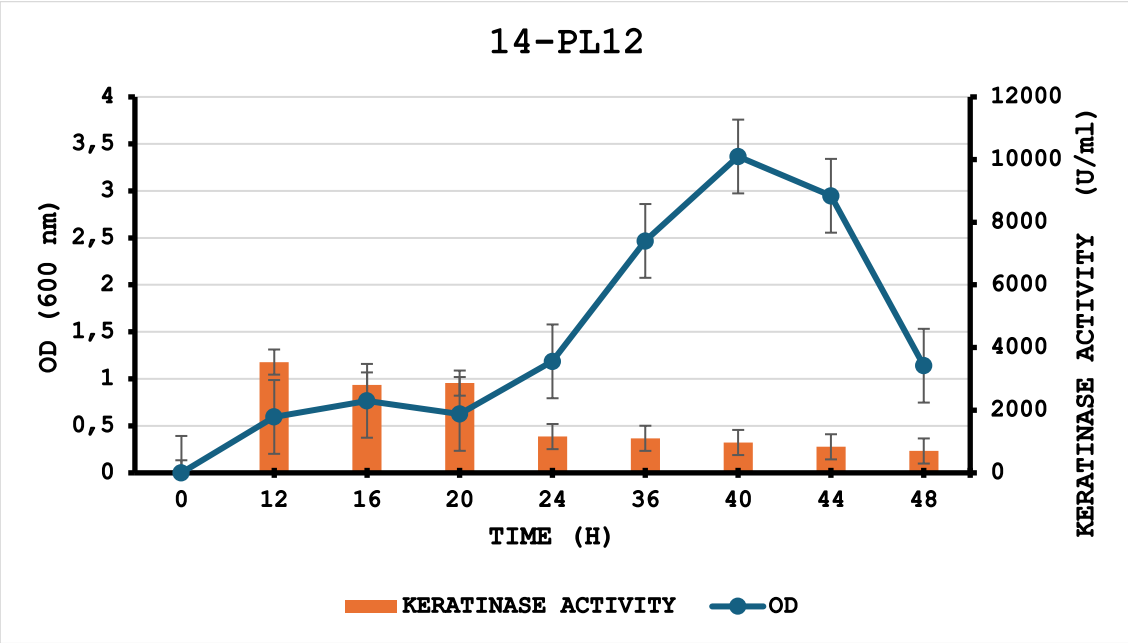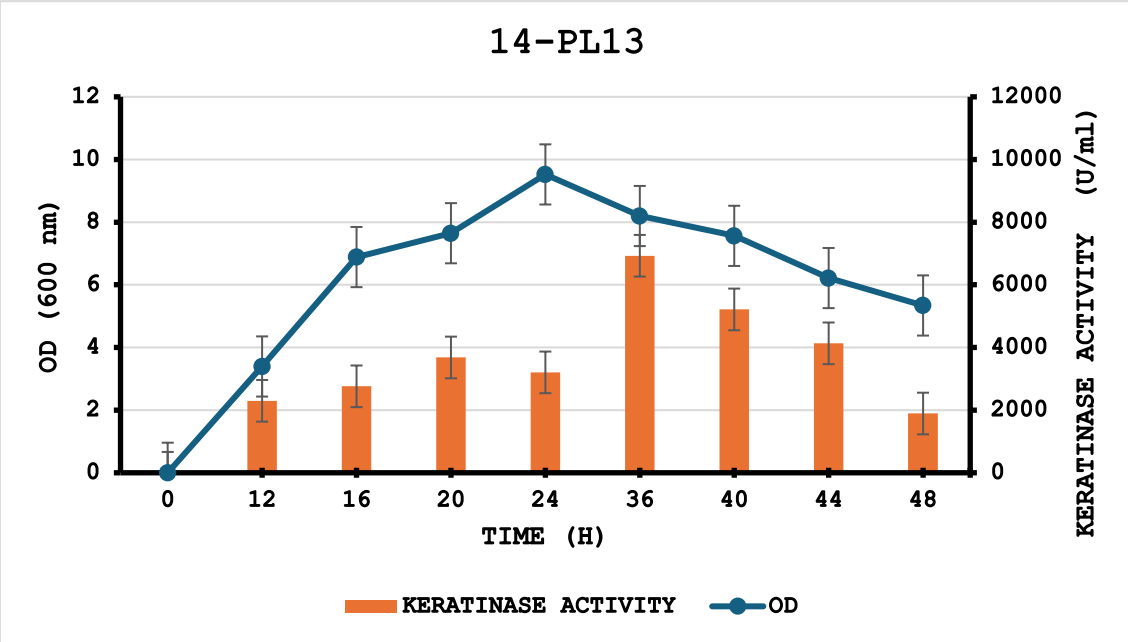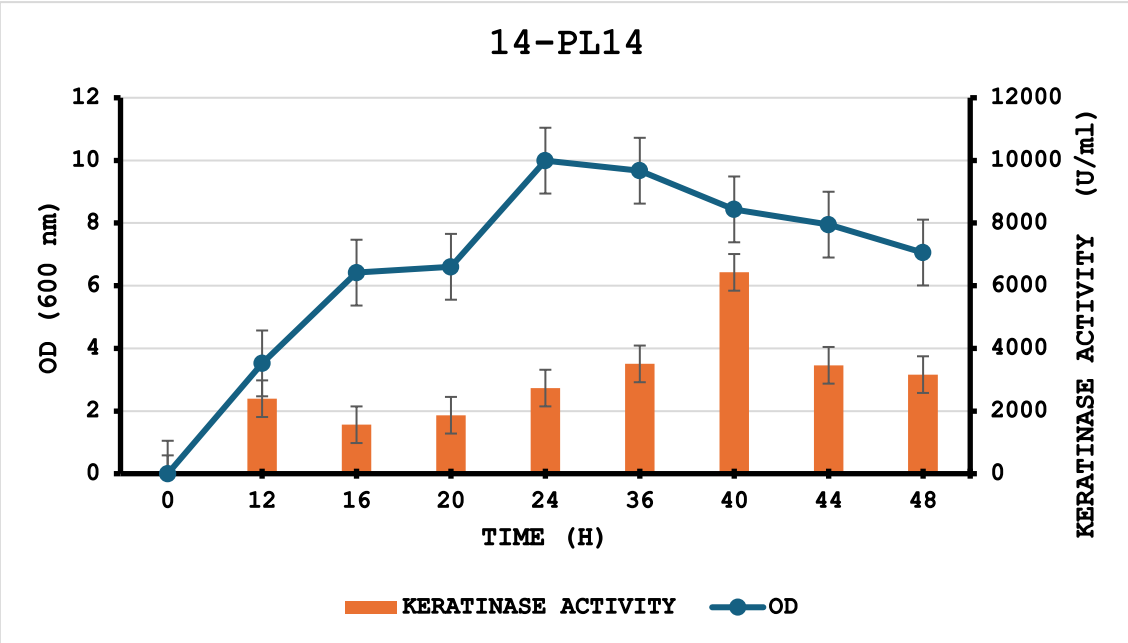

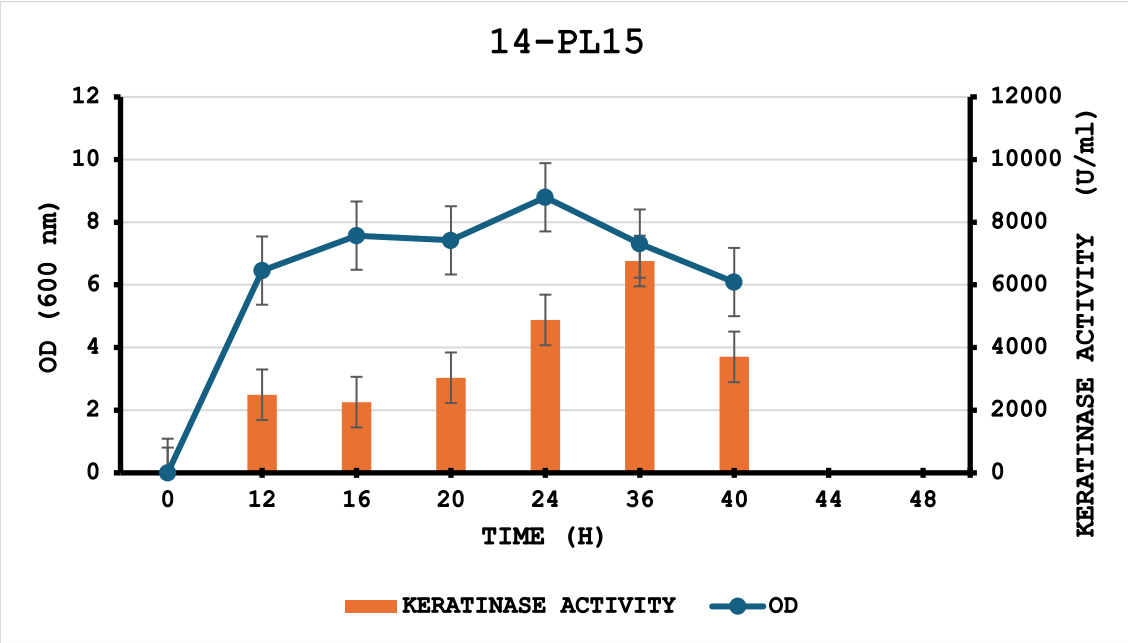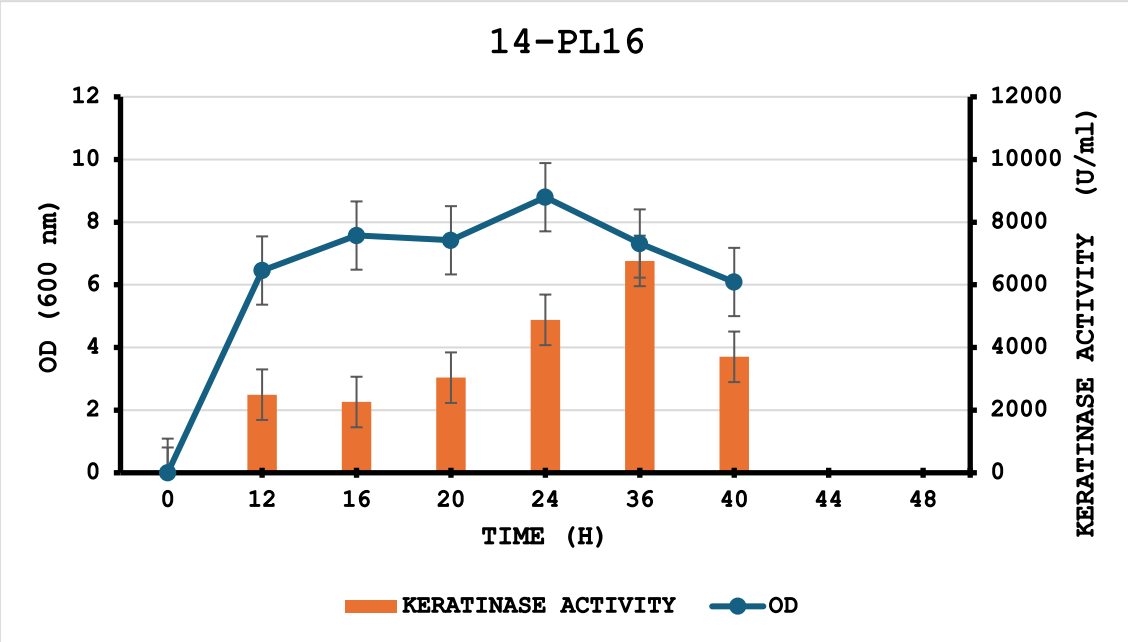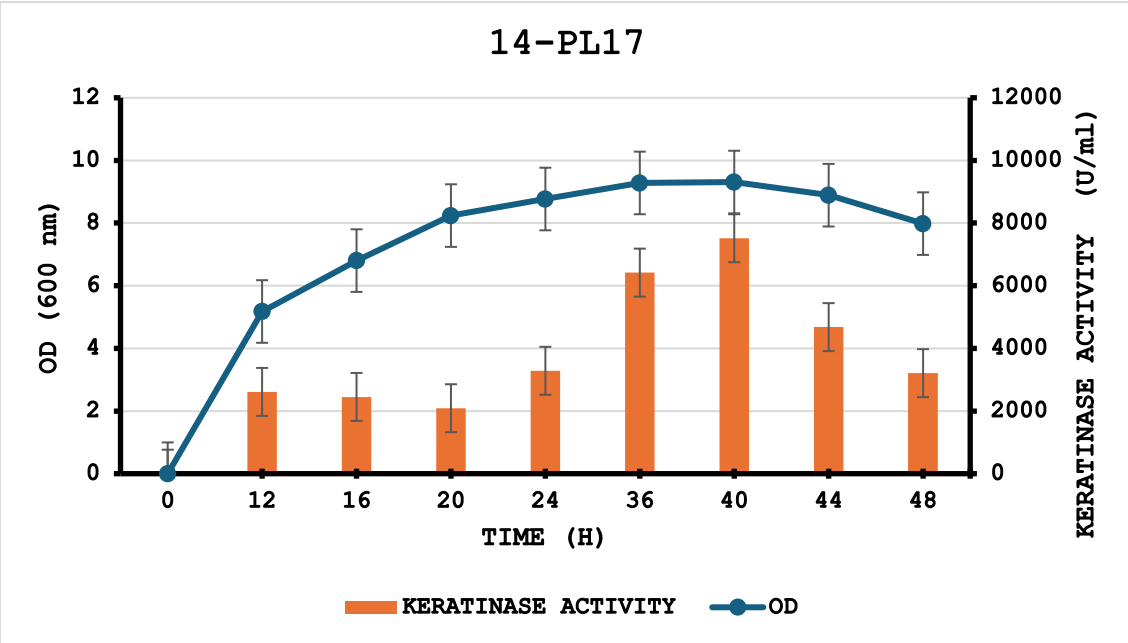

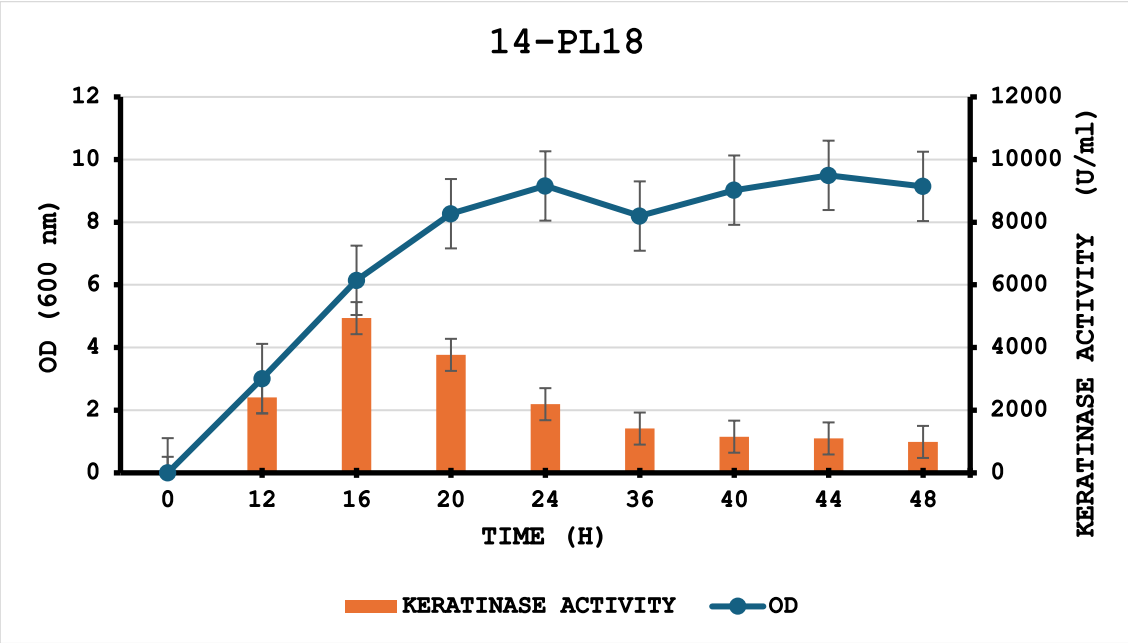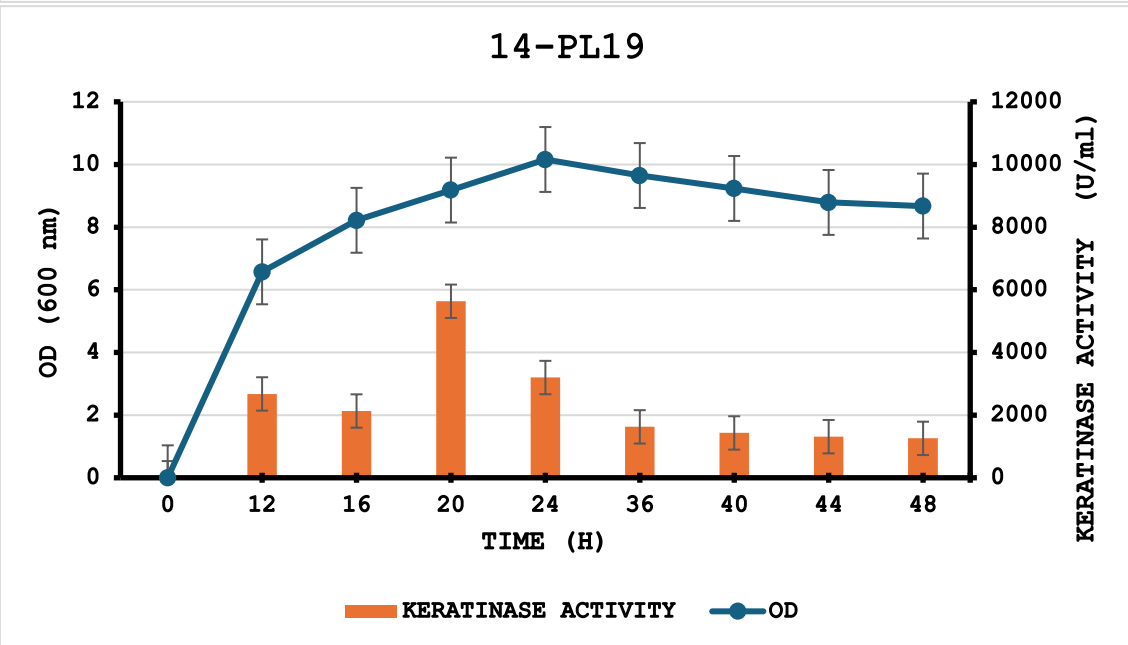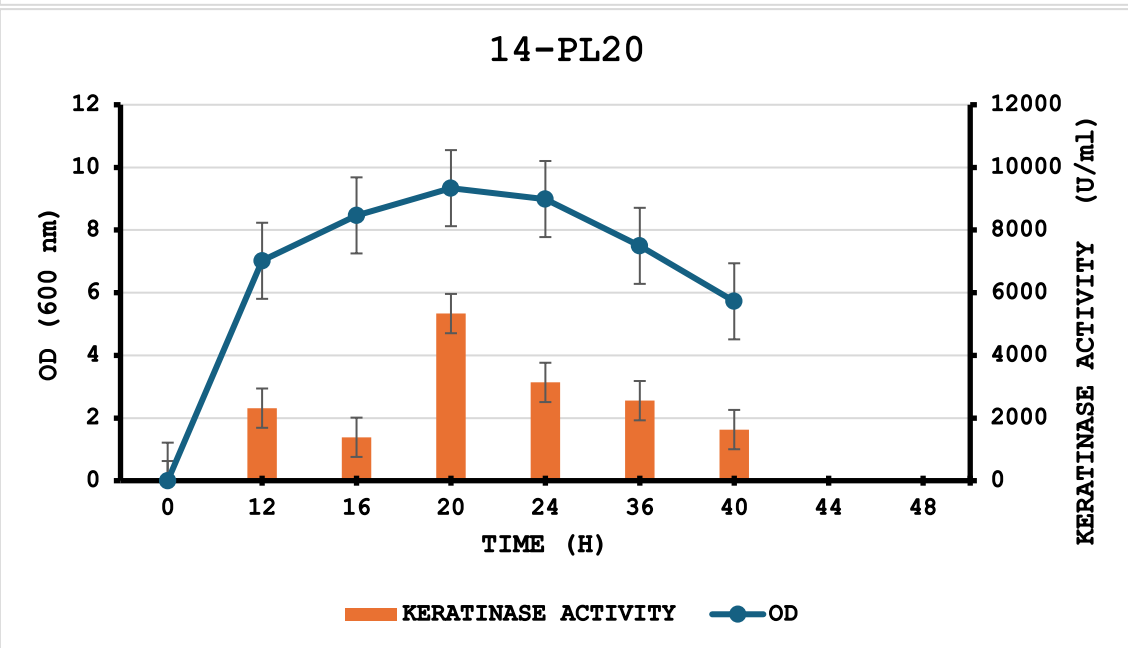

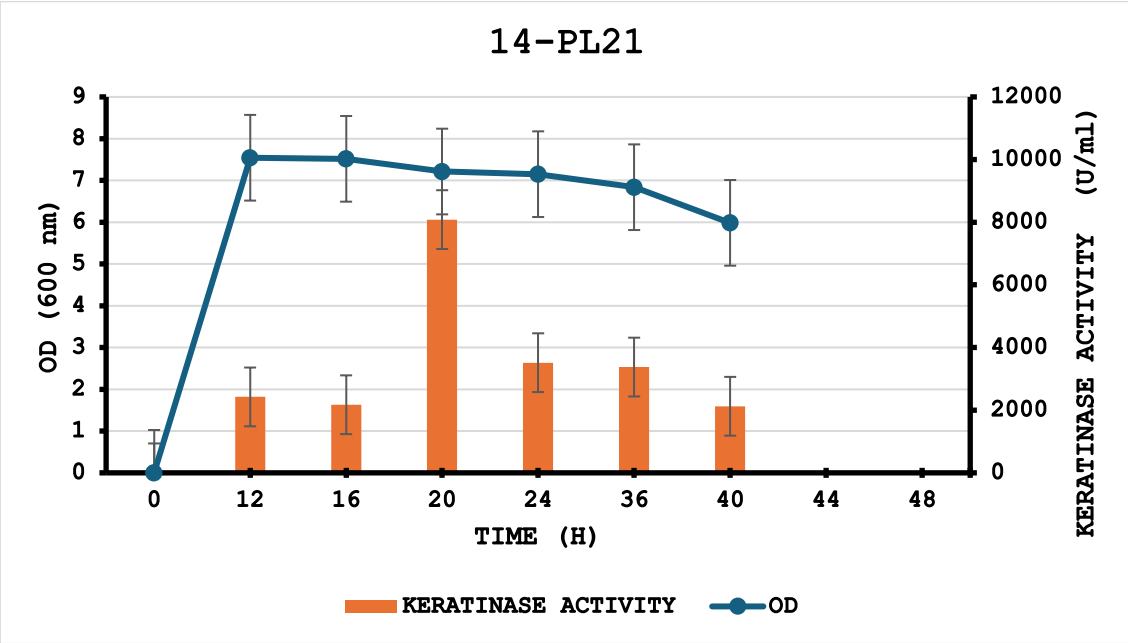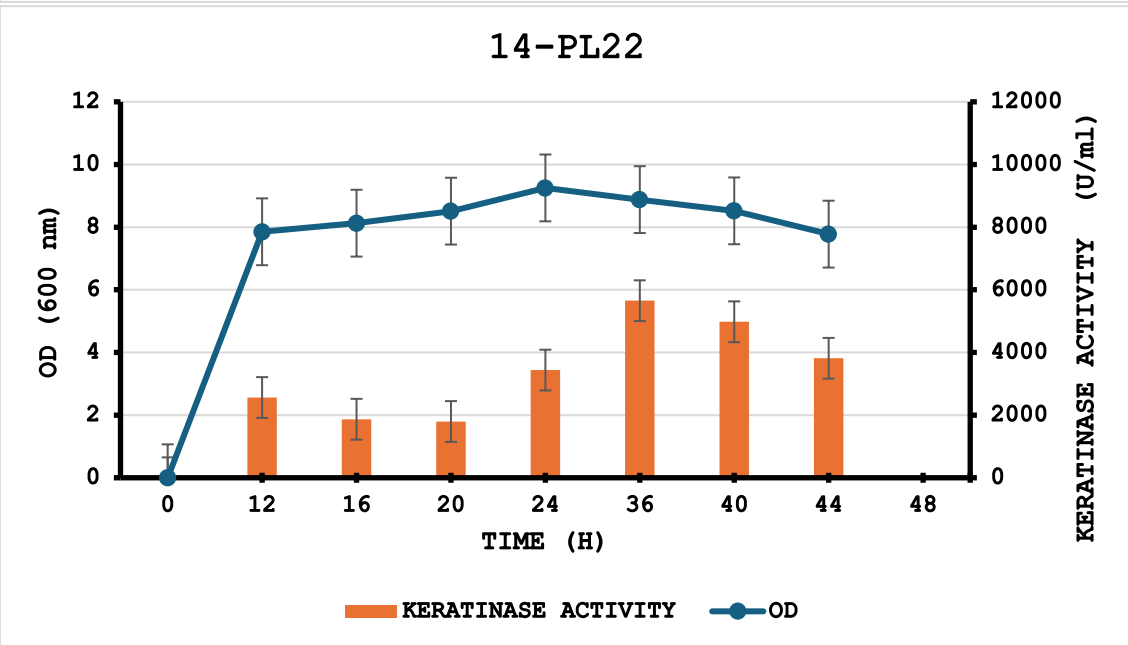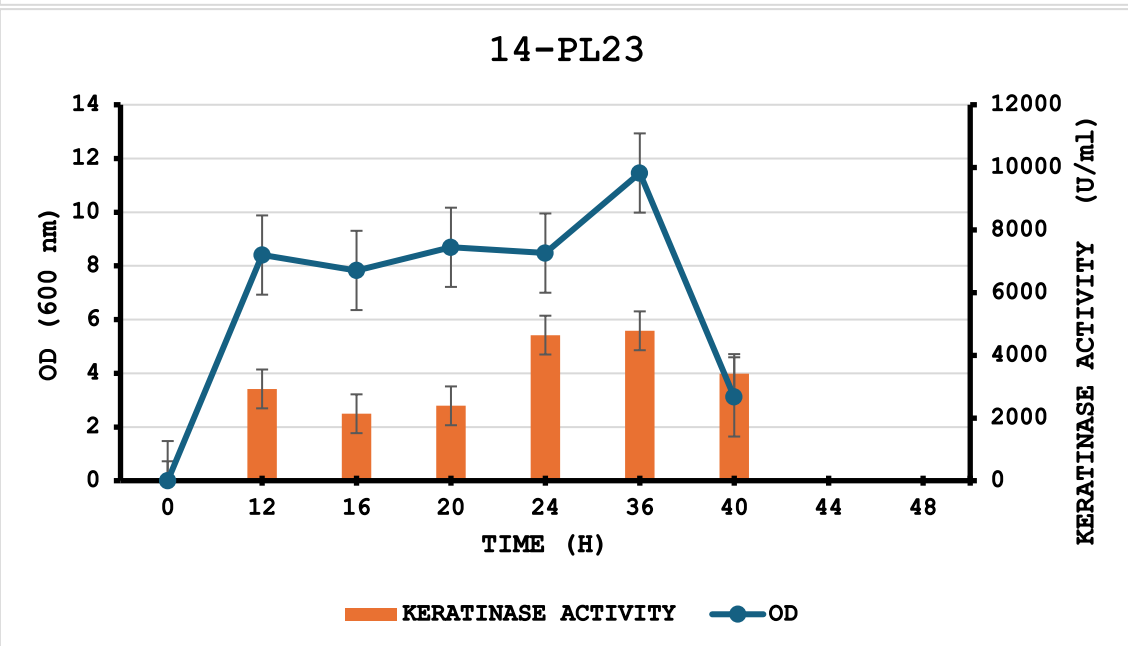

Supplement: Supplementary file 11 — Supplementary Material 6. Supplementary Figure S6: Growth and keratinase activity of the 132 bacterial isolates recovered during the fourteen-week experimental period. Error bars represent standard deviations calculated from three independent biological replicates. [file 11274_2026_4976_MOESM6_ESM.pdf]
