# Supplementary material for: Pilot-scale inoculum-free valorization of raw chicken feathers: ammonium recovery, keratinase production and community dynamics
Source: World J Microbiol Biotechnol. 2026 May 25;42(6):295. doi: 10.1007/s11274-026-04976-0 (PMC13201326; doi:10.1007/s11274-026-04976-0)
Supplement: Supplementary file 12 — Supplementary Material 7. Supplementary Table S1: Comprehensive list of the 132 bacterial isolates recovered over the fourteen-week operational period, including sampling time, selective agar medium, dilution factor, strain code and maximum keratinase activity measured in vitro. [file 11274_2026_4976_MOESM7_ESM.pdf]

Isolated strains from the reactor during the 100 days of the experiment

Nº isolated	Time (days)	Isolation agar	Dilution	Nomenclature	Keratinase activity (U/mL)
1	1	LB	1 × 10-6	1-PL01	<5000
2	1	LB	1 × 10-7	1-PL02	<5000
3	1	LB	1 × 10-8	1-PL03	<5000
4	1	LB	1 × 10-8	1-PL04	<5000
5	1	LB	1 × 10-8	1-PL05	<5000
6	1	LB	1 × 10-8	1-PL06	<5000
7	1	TSB	1 × 10-8	1-PL07	>5000
8	1	TSB	1 × 10-8	1-PL08	>5000
9	1	TSB	1 × 10-8	1-PL09	<5000
10	1	LB	1 × 10-8	1-PL10	<5000
11	1	LB	1 × 10-7	1-PL11	<5000
12	1	LB	1 × 10-6	1-PL12	<5000
13	1	LB	1 × 10-6	1-PL13	<5000
14	1	LB	1 × 10-6	1-PL14	<5000
15	1	LB	1 × 10-6	1-PL15	<5000
16	1	LB	1 × 10-8	1-PL16	<5000
17	1	LB	1 × 10-7	1-PL17	<5000
18	1	LB	1 × 10-7	1-PL18	<5000
19	1	LB	1 × 10-7	1-PL19	<5000
20	1	LB	1 × 10-7	1-PL20	<5000
21	8	LB	1 × 10-8	2-PL01	<5000
22	8	LB	1 × 10-8	2-PL02	<5000
23	8	LB	1 × 10-8	2-PL03	<5000
24	8	LB	1 × 10-8	2-PL04	<5000
25	8	LB	1 × 10-8	2-PL05	<5000
26	8	LB	1 × 10-8	2-PL06	<5000
27	8	LB	1 × 10-8	2-PL07	<5000
28	8	LB	1 × 10-7	2-PL08	<5000
29	8	LB	1 × 10-7	2-PL09	<5000
30	8	LB	1 × 10-7	2-PL10	<5000
31	8	LB	1 × 10-9	2-PL11	<5000
32	8	LB	1 × 10-8	2-PL12	<5000
33	8	LB	1 × 10-8	2-PL13	<5000
34	8	TSB	1 × 10-8	2-PL14	>5000
35	8	TSB	1 × 10-9	2-PL15	<5000
36	8	TSB	1 × 10-7	2-PL16	>5000
37	8	TSB	1 × 10-7	2-PL17	<5000
38	8	TSB	1 × 10-7	2-PL18	>5000
39	8	TSB	1 × 10-7	2-PL19	>5000
40	8	TSB	1 × 10-9	2-PL20	>5000
41	8	TSB	1 × 10-6	2-PL21	<5000
42	8	TSB	1 × 10-6	2-PL22	<5000
43	8	TSB	1 × 10-6	2-PL23	<5000
44	8	TSB	1 × 10-6	2-PL24	<5000
45	8	TSB	1 × 10-6	2-PL25	>5000
46	15	LB	1 × 10-4	3-PL01	<5000
47	15	LB	1 × 10-4	3-PL02	<5000

48	15	LB	1 × 10-3	3-PL03	<5000
49	15	TSB	1 × 10-6	3-PL04	>5000
50	15	TSB	1 × 10-4	3-PL05	<5000
51	15	TSB	1 × 10-4	3-PL06	<5000
52	15	TSB	1 × 10-4	3-PL07	<5000
53	15	TSB	1 × 10-3	3-PL08	<5000
54	15	TSB	1 × 10-4	3-PL09	<5000
55	22	LB	1 × 10-3	4-PL01	<5000
56	22	LB	1 × 10-3	4-PL02	<5000
57	22	LB	1 × 10-3	4-PL03	<5000
58	22	LB	1 × 10-3	4-PL04	<5000
59	22	LB	1 × 10-3	4-PL05	<5000
60	22	LB	1 × 10-4	4-PL06	<5000
61	22	LB	1 × 10-5	4-PL07	<5000
62	22	TSB	1 × 10-5	4-PL08	Not detected
63	22	TSB	1 × 10-5	4-PL09	>5000
64	22	TSB	1 × 10-4	4-PL10	>5000
65	22	TSB	1 × 10-4	4-PL11	<5000
66	22	TSB	1 × 10-4	4-PL12	<5000
67	22	TSB	1 × 10-4	4-PL13	>5000
68	22	TSB	1 × 10-4	4-PL14	>5000
69	22	TSB	1 × 10-3	4-PL15	<5000
70	29	LB	1 × 10-3	5-PL01	<5000
71	29	LB	1 × 10-3	5-PL02	<5000
72	29	LB	1 × 10-3	5-PL03	<5000
73	29	LB	1 × 10-5	5-PL04	>5000
74	29	LB	1 × 10-5	5-PL05	>5000
75	29	TSB	1 × 10-3	5-PL06	>5000
76	29	TSB	1 × 10-3	5-PL07	>5000
77	29	TSB	1 × 10-3	5-PL08	<5000
78	29	TSB	1 × 10-3	5-PL09	>5000
79	29	TSB	1 × 10-5	5-PL10	Not detected
80	29	TSB	1 × 10-5	5-PL11	<5000
81	36	LB	1 × 10-3	6-PL01	<5000
82	36	LB	1 × 10-3	6-PL02	<5000
83	36	LB	1 × 10-4	6-PL03	<5000
84	36	LB	1 × 10-4	6-PL04	>5000
85	36	LB	1 × 10-5	6-PL05	<5000
86	36	TSB	1 × 10-5	6-PL06	>5000
87	36	TSB	1 × 10-4	6-PL07	>5000
88	36	TSB	1 × 10-6	6-PL08	>5000
89	36	TSB	1 × 10-3	6-PL09	<5000
90	36	TSB	1 × 10-3	6-PL10	>5000
91	36	LB	1 × 10-5	6-PL11	<5000
92	36	LB	1 × 10-5	6-PL12	<5000
93	36	LB	1 × 10-5	6-PL13	>5000
94	36	LB	1 × 10-3	6-PL14	<5000
95	36	LB	1 × 10-3	6-PL15	>5000
96	43	TSB	1 × 10-5	7-PL01	>5000
97	43	TSB	1 × 10-5	7-PL02	<5000

98	43	TSB	1 × 10-5	7-PL03	>5000
99	43	TSB	1 × 10-4	7-PL04	>5000
100	43	TSB	1 × 10-5	7-PL05	>5000
101	43	TSB	1 × 10-3	7-PL06	>5000
102	43	TSB	1 × 10-3	7-PL07	>5000
103	43	LB	1 × 10-5	7-PL08	<5000
104	43	LB	1 × 10-5	7-PL09	>5000
105	43	LB	1 × 10-4	7-PL10	<5000
106	43	LB	1 × 10-4	7-PL11	>5000
107	43	LB	1 × 10-4	7-PL12	>5000
108	43	LB	1 × 10-4	7-PL13	>5000
109	43	LB	1 × 10-4	7-PL14	>5000
110	100	LB	1 × 10-4	14-PL01	<5000
111	100	LB	1 × 10-4	14-PL02	<5000
112	100	LB	1 × 10-4	14-PL03	>5000
113	100	LB	1 × 10-3	14-PL04	<5000
114	100	LB	1 × 10-3	14-PL05	<5000
115	100	LB	1 × 10-3	14-PL06	<5000
116	100	LB	1 × 10-3	14-PL07	<5000
117	100	LB	1 × 10-3	14-PL08	>5000
118	100	LB	1 × 10-3	14-PL09	<5000
119	100	LB	1 × 10-3	14-PL10	<5000
120	100	LB	1 × 10-3	14-PL11	<5000
121	100	LB	1 × 10-3	14-PL12	<5000
122	100	TSB	1 × 10-5	14-PL13	>5000
123	100	TSB	1 × 10-4	14-PL14	>5000
124	100	TSB	1 × 10-4	14-PL15	>5000
125	100	TSB	1 × 10-4	14-PL16	>5000
126	100	TSB	1 × 10-4	14-PL17	>5000
127	100	TSB	1 × 10-4	14-PL18	<5000
128	100	TSB	1 × 10-3	14-PL19	>5000
129	100	TSB	1 × 10-3	14-PL20	>5000
130	100	TSB	1 × 10-3	14-PL21	>5000
131	100	TSB	1 × 10-3	14-PL22	>5000
132	100	TSB	1 × 10-3	14-PL23	<5000
